# Systems-level quantification of division timing reveals a common genetic architecture controlling asynchrony and fate asymmetry

**DOI:** 10.15252/msb.20145857

**Published:** 2015-06-15

**Authors:** Vincy Wing Sze Ho, Ming-Kin Wong, Xiaomeng An, Daogang Guan, Jiaofang Shao, Hon Chun Kaoru Ng, Xiaoliang Ren, Kan He, Jinyue Liao, Yingjin Ang, Long Chen, Xiaotai Huang, Bin Yan, Yiji Xia, Leanne Lai Hang Chan, King Lau Chow, Hong Yan, Zhongying Zhao

**Affiliations:** 1 Department of Biology, Hong Kong Baptist UniversityHong Kong, China; 2 Center for Stem Cell and Translational Medicine, School of Life Sciences, Anhui UniversityHefei, China; 3 Division of Life Science and Division of Biomedical Engineering, The Hong Kong University of Science and TechnologyHong Kong, China; 4 Department of Electronic Engineering, City University of Hong KongHong Kong, China; 5 State Key Laboratory of Environmental and Biological Analysis, Hong Kong Baptist UniversityHong Kong, China

**Keywords:** asynchrony of cell division, automated lineaging, *C. elegans*, cell cycle length, cell division timing

## Abstract

Coordination of cell division timing is crucial for proper cell fate specification and tissue growth. However, the differential regulation of cell division timing across or within cell types during metazoan development remains poorly understood. To elucidate the systems-level genetic architecture coordinating division timing, we performed a high-content screening for genes whose depletion produced a significant reduction in the asynchrony of division between sister cells (ADS) compared to that of wild-type during *Caenorhabditis elegans* embryogenesis. We quantified division timing using 3D time-lapse imaging followed by computer-aided lineage analysis. A total of 822 genes were selected for perturbation based on their conservation and known roles in development. Surprisingly, we find that cell fate determinants are not only essential for establishing fate asymmetry, but also are imperative for setting the ADS regardless of cellular context, indicating a common genetic architecture used by both cellular processes. The fate determinants demonstrate either coupled or separate regulation between the two processes. The temporal coordination appears to facilitate cell migration during fate specification or tissue growth. Our quantitative dataset with cellular resolution provides a resource for future analyses of the genetic control of spatial and temporal coordination during metazoan development.

## Introduction

The development of metazoans with various cell types requires a tight control over cell division timing to accommodate cell fate specification and tissue growth. Failure in this control may result in detrimental effects such as tumorous growth and abnormal cell death. However, how cell division timing is regulated *in vivo* at the cellular level to ensure proper cell fate specification or tissue growth is poorly understood, especially during the proliferative stage of embryogenesis when cells undergo rapid divisions. Presumably, differential control of cell division timing between sister cells will lead to cell-specific division pace, which is defined as the duration of a given cell throughout the development of an organism and is used interchangeably with cell cycle length. Studies on single-cell organisms or cultured mammalian cells have contributed substantially to our knowledge of basic cell cycle control (Hartwell *et al*, 1974; Bloom & Cross, 2007; Coudreuse & Nurse, 2010), but have provided little information on the regulatory mechanisms of temporal coordination between cell divisions because such cells tend to divide independently of one another. Therefore, a model of metazoan development is required in order to examine the regulatory mechanisms of temporal coordination during the rapid cell divisions that give rise to different or the same cell type(s), referred to as cell fate specification and tissue growth, respectively. Cell fate specification is commonly achieved through the asymmetric segregation of fate determinants and/or the regulation of the division axis and site (Zhong, 2008; Munro & Bowerman, 2009; Li, 2013; Noatynska *et al*, 2013), whereas tissue growth involves a step-wise differentiation during cell division without the asymmetric segregation of cell fate determinants. In the context of this study, we refer to tissue growth as clonal development based on the expression of a tissue-specific marker and we define the cell fate determinant as any regulatory gene whose perturbation produces a defective fate specification.

**Figure 1:**
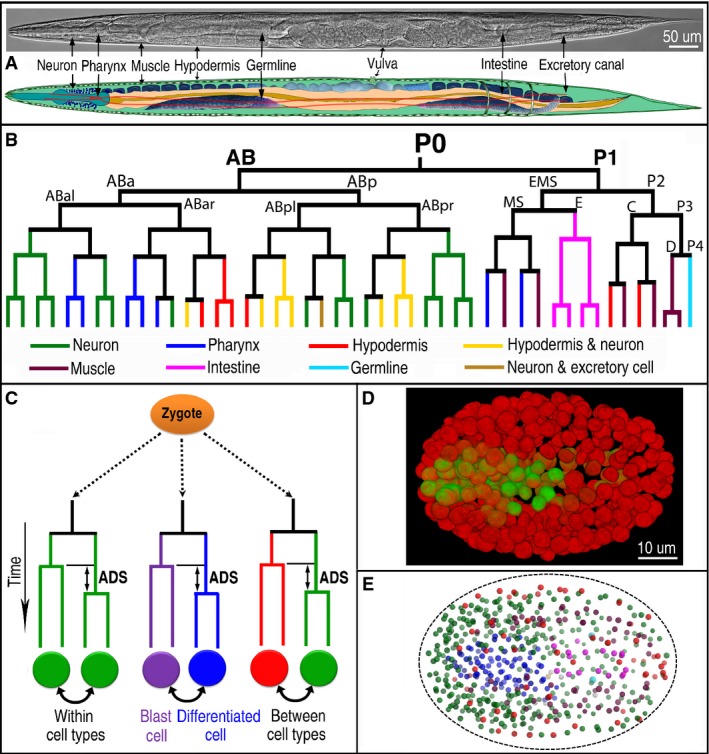
Overview of *Caenorhabditis elegans* cell fate map and division asynchrony

Previous studies of metazoan development have suggested an intertwined relationship between cell division and cell fate specification. For example, hypomorphic alleles of *cdk-1*, which encodes one of the major cyclin-dependent kinases (CDKs) that drive cell cycle progression, produce extra intestinal cells from blastomere C at the expense of hypodermis and muscle cells during *C. elegans* embryogenesis (Shirayama *et al*, 2006; Ishidate *et al*, 2014). Mutant and epistasis analyses have demonstrated that the proliferative fate of *C. elegans* germline stem cells depends on CDK-2/CYE-1 (Fox *et al*, 2011), two canonical components in cell cycle control. Genetic analysis has revealed that the Polycomb repressive complex 2 controls oocyte fate specification by regulating the activities of cyclin E and the CDK inhibitor *Dacapo* in *Drosophila* (Iovino *et al*, 2013). A kinome-wide RNA interference (RNAi) screen has showed that phosphorylation of the Wnt signaling receptor LPR5/6 requires CDK L63 in *Drosophila* (Davidson *et al*, 2009). So far, studies of metazoan division timing have mainly focused on a specific tissue. For example, a gain-of-function mutation in *cdc-25.1*, which encodes a cell cycle prompting phosphatase, or a loss-of-function mutation in a GATA type transcription factor, END-3, has produced an elevated division pace in the *C. elegans* E lineage that exclusively develops into the intestine (Clucas *et al*, 2002; Kostic & Roy, 2002; Boeck *et al*, 2011). Recent work on the cell cycle regulator, WEE-1.1, has demonstrated that the extended cell cycle lengths in Ea and Ep are dependent on the P2-EMS signal. However, a reduction in the cell cycle lengths of Ea and Ep caused by a mutation in *wee-1.1* was not associated with their fate specification during *C. elegans* embryogenesis, indicating that regulation of cell division timing can also be uncoupled from fate specification (Robertson *et al*, 2014). Importantly, coordination of division timing is frequently manifested as the asynchrony of divisions between sister cells (ADS), which is widely observed not only between the sister cells giving rise to different fates, but also between those developing into the same fate (Fig1). This indicates that another dimension of regulation coordinates division pace, which is linked to or independent of fate specification during metazoan development. Interestingly, a slowdown of the overall development rate of *C. elegans* embryo by altering the temperature or by introducing mutations resulted in a global decrease in division pace, but the relative timings between cells were well maintained (Schnabel *et al*, 1997; Nair *et al*, 2013), suggesting that the regulation of the overall development pace is independent of the control over the asynchrony between individual cells. Nevertheless, it remains largely unknown how the ADS is genetically controlled during fate specification or tissue growth, especially during the proliferative stage of embryogenesis.

A Nomarski micrograph (top) and a cartoon diagram (bottom) of a hermaphrodite adult showing major tissue types as indicated. Neuron, body-wall muscle, hypodermis, and excretory cell canal are not obvious in the Nomarski micrograph but are indicated based on their approximate positions.

A lineage tree of an early *C. elegans* embryo (47 cells) showing various cell fates (differentially color coded) derived from different lineal origins.

Schematic representation of ADS (asynchrony in cell division timing between sisters cells), which give rise to different or same cell type(s) as differentially color coded. Also shown is the comparison of asynchrony between the sister cells, one of which develops into a blast cell (purple) while the other becomes a terminally differentiated cell (blue) during embryogenesis.

3D projection of a *C. elegans* embryo of approximately 350-cell stage rendered with the fluorescence micrographs showing the expression of two lineaging markers, that is, pie-1::H2B::mCherry and H3.3::mCherry (red) and a pharynx-specific marker, PHA-4::GFP (green). See also Supplementary Movie S1 for expression dynamics and cell migrations.

A reconstructed space-filling model of nuclei within a wild-type embryo of approximately 350-cell stage based on the output of automated lineaging. Nuclei are differentially color-coded based on their fates in the same way as that in (B). Dash line marks the approximate boundary of the embryo.

**Figure 2:**
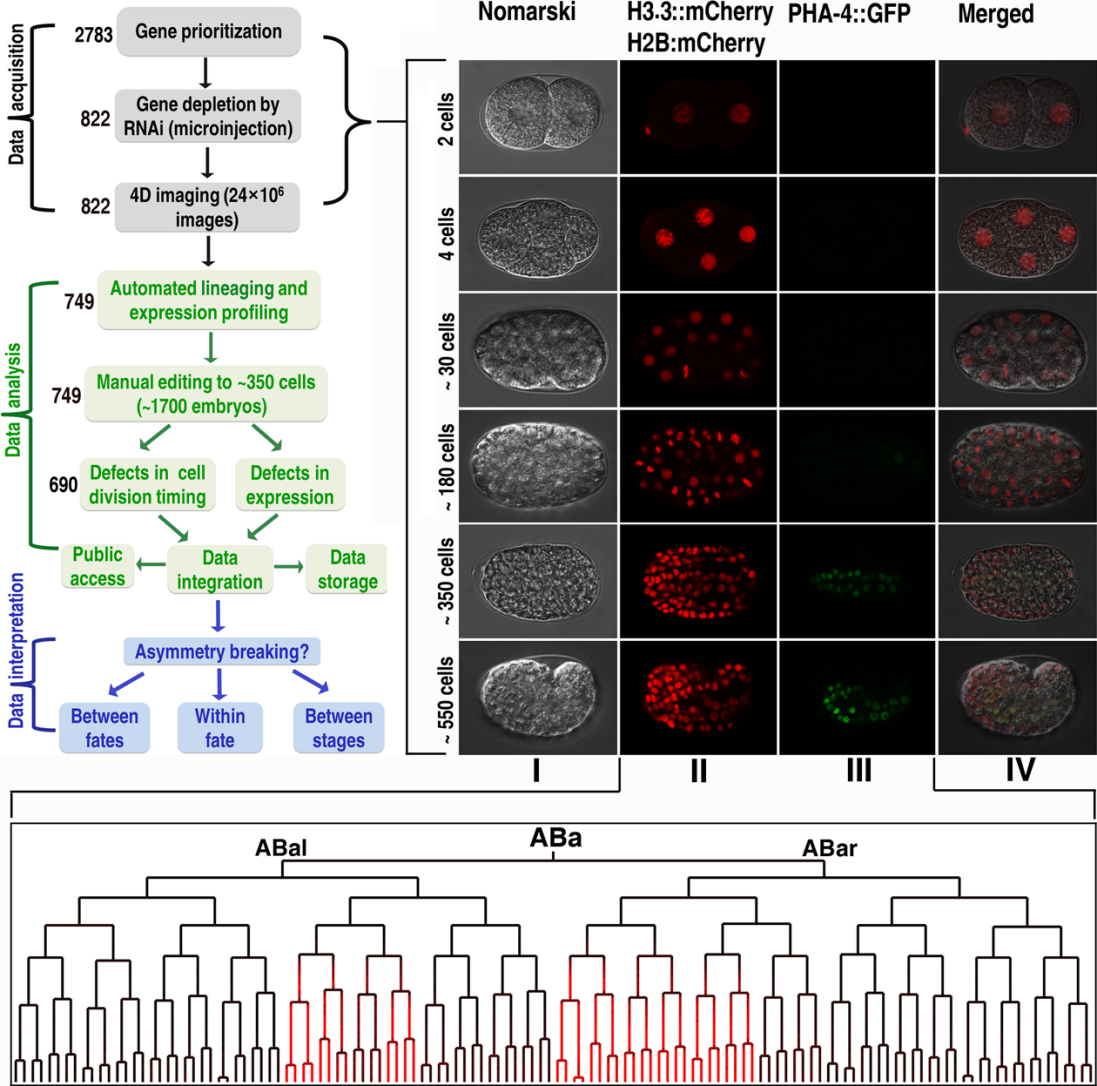
Experimental design and pipeline Top left, flowchart of this study (see also Supplementary Fig S3). Number of genes that go through each step is indicated. Top right, micrographs of *Caenorhabditis elegans* embryos at different developmental stages as indicated on the left. I, Nomarski micrographs of wild-type embryos at different stages; II and III, fluorescence micrographs showing the expression of lineaging and tissue markers (PHA-4) in the same embryos as those in I; IV, merged I, II and III. Bottom panel, a cell lineage tree of “ABa” along with the lineal expression of PHA-4 (colored in red) derived from fluorescence images of the lineaging and tissue markers using automated lineaging.

*Caenorhabditis elegans* is an excellent model to study the developmental control of cell division timing mainly because of its invariant development and widespread asynchronies in cell division during embryogenesis, which allows the unambiguous tracing of cell divisions from a one-celled fertilized egg to an adult worm (Figs1 and 2) (Sulston *et al*, 1983). Coordination of division pace between cells is particularly relevant during the proliferative stage of metazoan development, since precise timing of cell divisions is essential for ensuring proper cell migration and subsequent tissue formation, morphogenesis, and organogenesis (Supplementary Movie S1). Previous studies of division timings have mostly focused on heterochronic genes during postembryonic development in *C. elegans* (Gleason & Eisenmann, 2010; Ren & Zhang, 2010). However, developmental control of cell division timing appears to involve different mechanisms between embryonic and post-embryonic stages. For instance, loss-of-function mutations in hetero-chronic genes change the patterns of cell cycle progression during larval development but does not result in similar changes in a developing embryo (Ambros, 2001). Therefore, the identification of genes involved in the differential control of division pace during metazoan embryogenesis is critical for understanding the genetic regulation of temporal coordination.

One of the major challenges in the analysis of temporal regulation is to systematically and quantitatively document division timings with high spatiotemporal resolution during metazoan development. Therefore, studies on the *in vivo* control of cell division have mainly focused on the early stages of embryogenesis when an embryo contains only a handful of cells. Such studies have successfully defined the regulatory pathways controlling cell division asynchrony or polarity (Brauchle *et al*, 2003; Colombo *et al*, 2003; Budirahardja & Gonczy, 2008; Galli & van den Heuvel, 2008; Li, 2013), chromosome segregation (van der Voet *et al*, 2009), cytokinesis (Sonnichsen *et al*, 2005; Chartier *et al*, 2011), and centrosome assembly (Greenan *et al*, 2010; Narasimhachar *et al*, 2012). As hundreds of cells accumulate within a developing embryo, it becomes extremely difficult to systematically document the cellular and molecular events through direct observation. To facilitate a systems-level documentation of cellular events within a developing embryo, a series of tools have recently been developed, which allow the manual or automated tracing of cell lineage and the profiling of gene expression during *C. elegans* embryogenesis (Schnabel *et al*, 1997; Bao *et al*, 2006; Murray *et al*, 2008). In particular, the availability of automated lineaging tools permits the systematic quantification of cell cycle lengths and marker gene expression up to the 350-cell stage during *C. elegans* embryogenesis with limited human intervention. This has recently been successfully used for large-scale profiling of transcription-factor expression with single-cell resolution (Murray *et al*, 2012) and for *de novo* inference of a systems-level regulatory network of *C. elegans* founder cell specification (Du *et al*, 2014). The technique has also been used for functional characterization of individual genes or pathways (Zhao *et al*, 2010; Shao *et al*, 2013). To dissect the systems-level molecular architecture of temporal regulation during *C. elegans* embryogenesis, we have carried out a genetic screen using a combination of RNAi and automated lineaging, aiming at the identification of genes controlling the asynchrony of division between sister cells during cell fate specification or tissue growth.

## Results

### A pipeline for systems-level profiling of cell division timing during *C. elegans* embryogenesis

To identify genes that regulate ADS, we have established a pipeline consisting of gene perturbation with RNAi by microinjection, acquisition of time-lapse three-dimensional (4D) images of *C. elegans* embryogenesis, and automated lineaging (Fig2; Supplementary Fig S1). Given the limited resources available for the imaging and manual curation of the output of automated lineaging, we prioritized the genes to be included in the pipeline based on their degree of conservation and the reported phenotypes upon perturbation (Supplementary Fig S1A; see Materials and Methods). We applied the framework described above to a total of 822 genes functioning in various pathways and screened for defects in ADS that produce a single or different cell type(s) (Supplementary Tables S1 and S2).

We performed the RNAi and 4D imaging in a manner similar to that described previously (Sonnichsen *et al*, 2005; Murray *et al*, 2006; Green *et al*, 2011). We collected three replicate movies per imaging session with a single tissue marker for each perturbed gene. We acquired images of perturbed embryos mostly with the strain RW10425, which expresses PHA-4 as a tissue marker with a few exceptions, including HLH-1 or NHR-25 as a tissue marker (Supplementary Tables S1 and S3). Automated lineaging and expression profiling were performed as described (Murray *et al*, 2013; Shao *et al*, 2013). Although a wild-type embryo produces over 550 cells during embryogenesis (Sulston *et al*, 1983), it takes a well-trained technician approximately 0.5–2 h and 8–16 h to manually curate the image data of an embryo up to 350 and 550 cells, respectively, depending on the image quality (Richards *et al*, 2013). Therefore, we chose to routinely curate all the images up to approximately 350 cells for all the wild-type and perturbed embryos. For a subset of the perturbed embryos, which either demonstrated early arrest before 350-cell stage or produced zygotic depletion of lineaging marker expression upon perturbation, thus preventing effective editing further into embryogenesis, we curated the images up to the last editable time point.

Given the arbitrary cutoff time point for the image curation, many cells may not have reached the dividing time point at the cutoff, resulting in some ambiguity in its division timings. To account for this, we excluded all division timings for the cells that did not divide up to the cutoff time point for both wild-type and perturbed embryos. We started imaging from two- to four-cell embryos to permit automated naming (Bao *et al*, 2006). Therefore, the timings for the first two rounds of division, that is, those of AB, P1, ABa, ABp, EMS, and P2, were also excluded in our analysis. As such, we first quantified division timings for a total of 351 cells in wild-type embryos (Supplementary Table S4). We next quantified the expression of a single tissue marker for each embryo. We finally computed the ADS and intensity of marker expression for all the perturbed embryos. In total, we have inactivated 822 genes and collected nearly 2,700 movies, approximately 1,700 of which were curated up to 350-cell stage or to the last editable time point (Fig2; Supplementary Table S1). A total of 749 of the 822 genes went through the complete pipeline (meaning a combination of RNAi, imaging and automated lineaging with at least two manually curated embryos). Eleven out of the remaining 73 genes went through the pipeline but with a single replicate because all other replicates demonstrated early embryonic arrest that was frequently associated with defective cytokinesis or nuclear separation, thus preventing effective editing. The remaining 62 genes went through the RNAi and imaging steps but not through the step of automated lineaging due to either early arrest (most of these perturbed embryos arrest before 50-cell stage) or failure in producing any embryos by the injected animals (Supplementary Table S1). The lineaging data of the 11 genes and the final phenotypes of the perturbed embryos for the 62 genes can be found in our online database called “Phenics” (http://phenics.icts.hkbu.edu.hk/). Our lineaging analysis provides systematic and quantitative information on cell division timing, tissue-marker expression, and cell migration at single-cell resolution with 1.5-min intervals for both wild-type and perturbed *C. elegans* embryos.

### Validation of the pipeline

**Figure 3:**
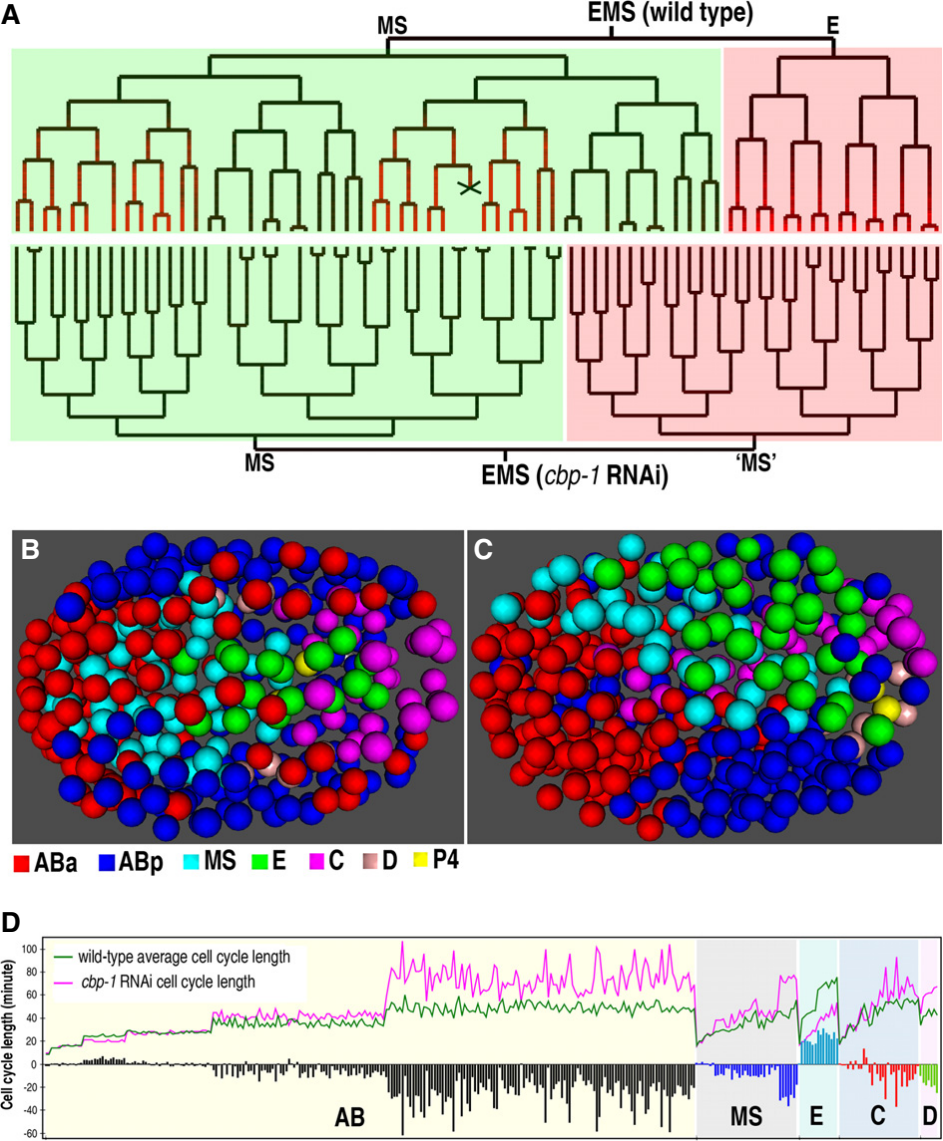
Validation of experimental pipeline by depletion of CBP-1, which is previously known to produce extra cell divisions (see also Supplementary Fig S3)

To test whether our pipeline was capable of detecting known embryonic defects resulting from the depletion of previously characterized genes, we examined the cell lineage and tissue-marker expression after depletion of *cbp-1*, *pop-1*, *lit-1*, and *nhr-25*. *cbp-1* encodes a homolog of the mammalian transcription co-factor, CREB-binding protein (CBP), which has histone acetyltransferase activity that is essential for its roles in regulating transcription and cell fate specification (Victor *et al*, 2002; Eastburn & Han, 2005). Inhibition of CBP-1 activity produced extra cell divisions in *C. elegans* embryos (Shi & Mello, 1998). Consistent with this, we found that depletion of CBP-1 led to a homeotic transformation from “E-” to an “MS”-like fate and a failure in gastrulation as judged by the defects in both cell lineage and cell migrations (Fig3A–C). The fate transformation from “E” with a smaller number of daughters to “MS” with a higher number of daughters likely explains the extra cell divisions observed previously. A side-by-side comparison of the division timings upon perturbation against those of the wild type revealed an E-lineage-specific acceleration but an otherwise ubiquitous deceleration in division in the remaining lineages (Fig3D). In addition, CBP-1 inactivation led to a loss of PHA-4 expression (Fig3A). Intriguingly, cell cycle lengths in *cbp-1* RNAi embryos were not only greater in magnitude, but also showed a much higher variability compared with the wild type, suggesting that the gene is possibly functioning as a phenotypic buffer or capacitor for achieving phenotypic robustness of temporal control of cell division (Levy & Siegal, 2012). Notably, CBP-1 depletion also led to a loss of tissue-marker expression (Fig3A), indicating defective fate specification. It remains to be determined whether the increased variability in cell cycle length is directly related to the observed defects in fate specification. To systematically identify all genes that produce a similar buffering effect, we computed the significant dispersion in cell cycle lengths of perturbed embryos from that of wild-type average for all the “AB” daughters (see Materials and Methods). A total of 66 genes show similar a buffering effect as that of *cbp-1* (Supplementary Fig S2). Follow-up analyses are required to evaluate whether these genes function as capacitors for temporal control of cell division. The application of our pipeline to *pop-1* and *lit-1* successfully recapitulated previously reported phenotypes; that is, inactivation of *pop-1* led to a homeotic fate transformation of “MS”- to “E”-like fate while inactivation of *lit-1* produced the opposite transformation (Supplementary Fig S3A–C) (Lin *et al*, 1995; Rocheleau *et al*, 1997; Thorpe *et al*, 1997). In addition, inactivation of POP-1 and LIT-1 led to an apparent defect in cell migration. For example, the positions of “ABa” and “ABp” daughters are symmetric in a wild-type embryo (Supplementary Fig S3E), but became asymmetric in POP-1- (Supplementary Fig S3F) or LIT-1 (Supplementary Fig S3G)-depleted embryos. The fate transformation that was observed in our lineaging analysis is consistent with those reported previously (Supplementary Table S5). To further validate the specificity of the RNAi, we injected the dsRNA against one of the tissue markers, *nhr-25*, into the animals expressing both NHR-25 and the lineaging markers (Supplementary Table S3). NHR-25 is a nuclear hormone receptor that is specifically expressed in the hypodermis and is required for hypodermis specification and molting by regulating its downstream targets (Chen *et al*, 2004; Shao *et al*, 1998). Lineaging analysis revealed that the RNAi abolished the expression of NHR-25 in all lineages and produced apparent defects in the cell division timings of Caapp compared to the wild type (Supplementary Fig S3D), but led to few cell-migration pattern defects (Supplementary Fig S3H). Taken together, our pipeline was capable of specifically capturing previously reported defects in cell division, fate transformation, and cell migration, allowing systematic screening of defects in cell division timings during *C. elegans* embryogenesis.

A Shown are “EMS” cell lineage trees of a wild-type (top) and a *cbp-1* RNAi (bottom) embryo, respectively. Lineal expression of PHA-4::GFP is shown in red, cell death is indicated with an “X”, and “E” and “MS” lineage are shaded in red and green, respectively, in the wild-type embryo. Note that “E” became an “MS”-like fate in the CBP-1-depleted embryo.

B, C Space-filling models of cell nuclei from a wild-type embryo (approximately 350-cell stage) and a *cbp-1* RNAi embryo (terminal stage when the embryo died presumably due to a gastrulation failure) showing cell positions. Shown are the ventral views of the embryos with anterior to the left. Cells are differentially color-coded according to their blastomere cell origins.

D A pairwise comparison of cell cycle lengths of each cell between wild-type average (green) and *cbp-1* RNAi embryos (pink). Horizontal axis denotes the progeny of founder cells (differentially color coded), which are ordered first by the round of division then by the cell name alphabetically. Vertical axis indicates the cell cycle lengths in minutes. The net differences in cell cycle lengths between RNAi and wild-type embryos are plotted and color coded based on their lineal origins. Note that division pace is faster in E progeny but is mostly slower in the progeny of remaining lineages after the perturbation.

### A framework for screening genes regulating ADS

**Figure 4:**
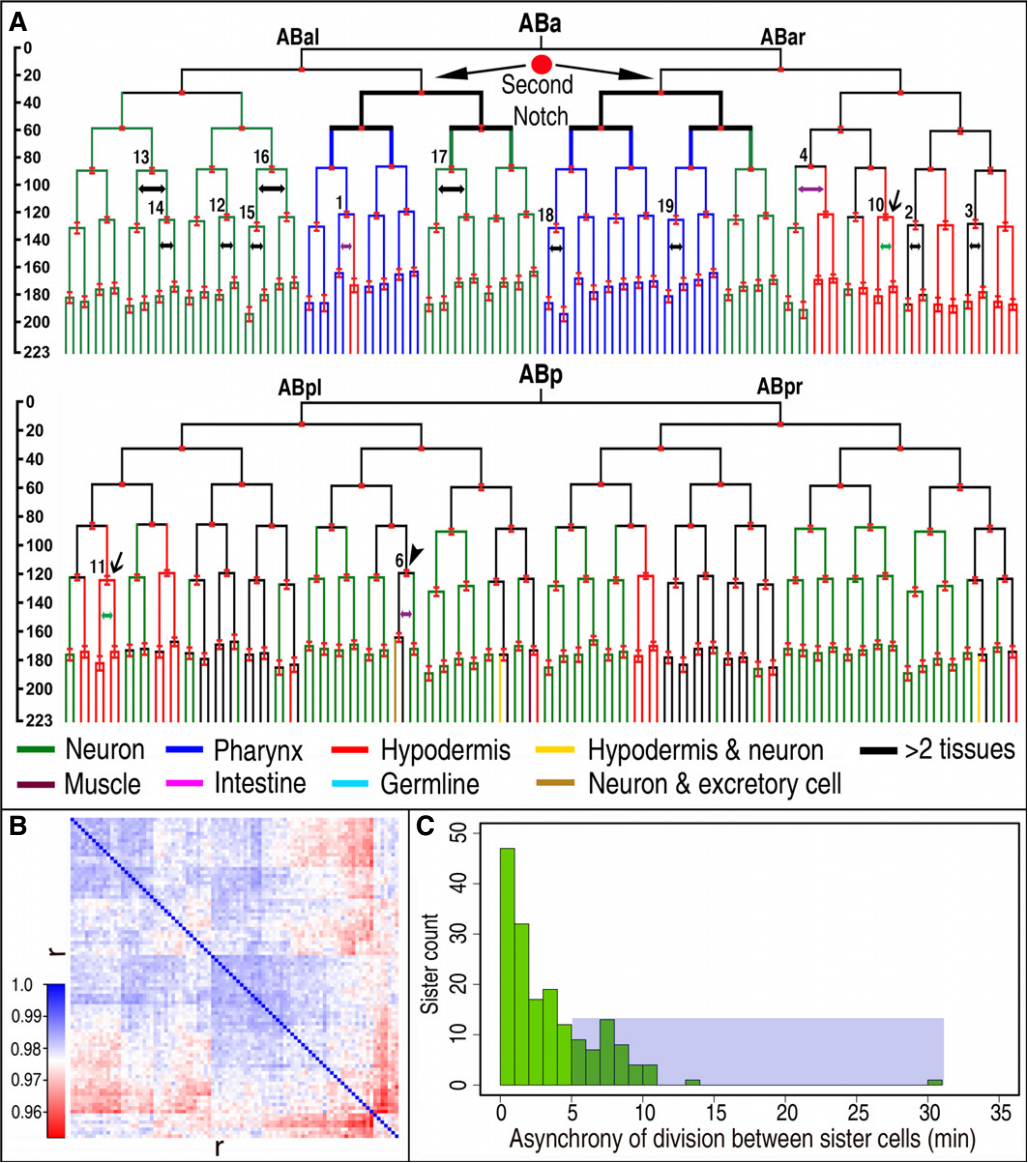
Reproducibility of cell cycle lengths

To build a framework for the systematic quantification of ADS during *C. elegans* embryogenesis, we performed automated lineaging for a total of 93 wild-type embryos expressing various tissue markers (Supplementary Table S3). Two embryos arrested with few cell divisions during imaging and were excluded for subsequent analyses. We extracted the cell cycle lengths for a total of 351 parental cells from each of the remaining 91 embryos and evaluated their variations between one another (Supplementary Table S4, Fig4B). The overall cell cycle lengths were highly reproducible among wild-type embryos, which bear a correlation coefficient (*r*) of at least 0.987 when compared with previous results (Moore *et al*, 2013; Richards *et al*, 2013) (Fig4A and B; Supplementary Fig S4A). Intriguingly, the standard deviations of cell cycle lengths increased with developing time during later generations (Supplementary Fig S4B), suggesting a tighter control over cell division timing in early embryonic stages than in later stages. It also remains possible that the increased variations in cell cycle length in the later generations could be a product of the cumulative variations inherited from earlier generations.

**Figure 5:**
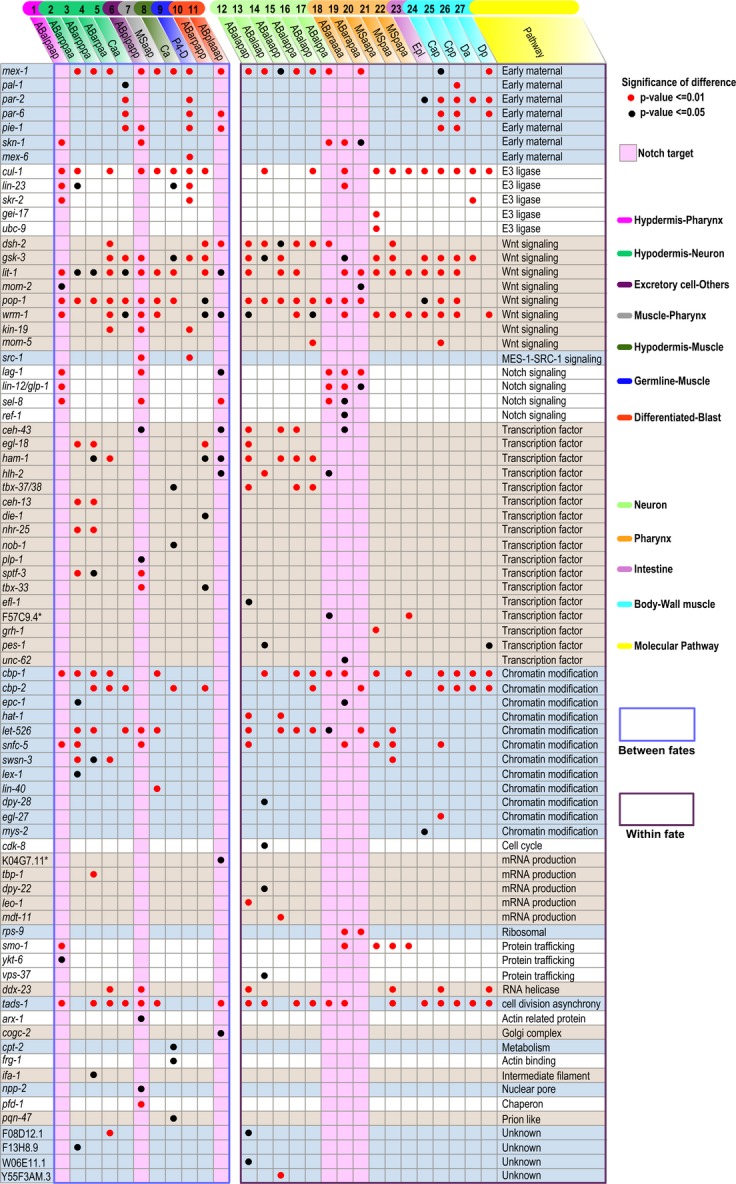
List of genes whose inactivation leads to a significant and over 50% reduction in ADS Left and right columns (separated by a white-space column) show two categories of genes whose perturbation affects division asynchrony between different fates (left) or within the same fate (right), respectively, in at least one sister pair. Genes are empirically grouped into discrete pathways on the rightmost column and are ordered first by shared or unique regulation between the two categories and second alphabetically within each pathway. Names of the parental cells for ADS calculation are shown on the top and color coded based on the fate(s) their daughters give rise to. The numerical code for each cell shown above the cell name corresponds to that in Fig4A and Supplementary Fig S4A. Red and black dots indicate a significant level of *P* < 0.01 and *P* < 0.05, respectively, upon perturbation, while the remaining empty spaces represent a gene perturbation that does not produce significant deviation in ADS from wild type for the cell (see also Supplementary Table S10). Pink columns indicate the cells that are known to receive Notch signal. *, inferred from sequence homology.

“ABa” (top) and “ABp” (bottom) cell lineage trees derived from average cell cycle lengths of 91 wild-type embryos of approximately 350-cell stage with standard deviations indicated as red bars on division nodes (see “P1” lineage tree in Supplementary Fig S4A). Cell fates of “ABa” or “ABp” descendants are differentially color coded in a way similar to that in Fig1 (note some fates are labeled with extra depth here that is not observed in Fig1). Developing time in minutes is shown on the left starting from the last time point of “ABa” to the cutoff time point of 350-cell stage. Sister pairs used in screening for asynchrony are indicated with two-headed arrows that are color-coded in black, purple, and green to denote the following three types of division, respectively, that is, those giving rise to the same or different cell type(s) or one daughter to terminally differentiated cell and the other to a postembryonic blast cell (see also Fig1C). Parent of the sister cells is labeled with a numerical code corresponding to that in Fig5 below. Sublineages receiving Notch signaling (red dot) are indicated with two arrows and are highlighted in bold. Precursor of the excretory cell (ABplpapp) is indicated with an arrowhead. Precursor whose one daughter becomes terminally differentiated while the other develops into postembryonic blast cell is indicated with an arrow.

A heat map of mutual Pearson correlation's coefficient (*r*) of cell cycle lengths between 91 individual wild-type embryos. Both horizontal and vertical axes denote the coefficient of an individual embryo against another.

Distribution of sister-pair count based on their asynchrony (in minutes) in wild-type 350-celled embryos. Horizontal axis denotes asynchrony, and the vertical axis represents cell count. Asynchronies between sister pair that is longer than 5 min are shaded.

ADS becomes apparent from the 5th and 4th round of divisions in the sublineages of “AB” and “P1”, respectively (Fig4A and B; Supplementary Fig S4A and C). A total of 46 pairs of sister cells demonstrated an average ADS that is over 5 min in the 91 wild-type embryos (Supplementary Table S6), which became the targets of our screening. To characterize the genetic control over ADS during tissue growth and cell fate differentiation, we screened for genes that produced a significant ADS reduction in perturbed embryos, compared to wild type. To increase the specificity of our screening, we only included in our analysis genes whose depletion produced at least a 50% reduction in the ADS compared with the average ADS of wild-type embryos in at least one pair of sister cells. Even though most of the replicate embryos showed reproducible phenotypes in division timing, there were some replicates that seemed to produce inconsistent results in a particular cell. We therefore required that the above criteria should be met in at least two replicates. To further increase the specificity of the screening, we required that the perturbed embryos developed at a relatively “normal” speed (see “Quantification of cell cycle lengths” in Materials and Methods). A total of 59 genes were excluded for further analysis due to their overall slowdown in developing speed (Supplementary Table S7). For example, *mex-5* was excluded from our data analysis because inactivation of the gene resulted in a homeotic cell fate transformation from “ABa” and “ABp” into two copies of “EMS”-like fate (Supplementary Fig S5), leading to a net loss of approximately 130 cells up to a time point comparable to that of a 350-celled wild-type embryo. This produces an embryo with spurious “slowdown” in developing speed, and therefore, the gene was excluded from the subsequent analyses. In sum, a total of 690 out of the 749 genes with two curated embryos were retained for the subsequent ADS screening.

### Coordination of cell division timing during cell fate specification

To screen for genes that control ADS during cell fate specification, we focused on the division asynchrony between two daughters of a subset of 11 cells of different lineal origins, which develop into two distinct cell types (Fig5, left panel). This subset was prioritized based on the following criteria. First, the average asynchrony is at least 5 min between their two daughters in 91 wild-type embryos. Second, the chosen cells represented as many sublineages and combinations of cell types as possible. Third, two cells, ABarpapp and ABplaaap, were included in this category though their terminal fates are the same, that is, hypodermis. This is because their anterior daughters become terminally differentiated into hypodermis during embryogenesis, whereas their posterior daughters develop into postembryonic blast cells, meaning that they stop dividing temporarily during embryogenesis and resume their division and cell fate specification only during the postembryonic stage (Sulston *et al*, 1983) (Figs4A and 5). Despite the same terminal fate of the two daughters, we included these two cells in the category of cell fate specification due to the distinct differentiation status of their two daughters during embryogenesis (Fig1C). Therefore, all of the blastomere lineages were included in this category besides the E sublineage, which exclusively develops into the intestine (Fig5; Supplementary Fig S4A).

We identified a total of 58 genes or gene pairs whose depletion reproducibly produced a significant and over 50% reduction in ADS of at least one of the 11 cells compared to that of the wild-type average (Fig5, left panel). We empirically classified the 58 genes into the following functional groups/pathways, including early maternal regulation, protein degradation/E3 ligase, signal transduction, transcriptional regulation/transcription factor, chromatin modification/chromatin modifier (defined as those involved in histone modification or chromatin remodeling), and various others based on their known or inferred functions (Fig5, right panel). Surprisingly, 41 out of the 58 genes (member of the first seven functional groups) are also known to mediate cell fate specification directly or indirectly (Supplementary Fig S6, Supplementary Tables S5 and S8), indicating that a similar genetic architecture is used for both asynchrony and fate specification. Out of the 41 genes, 7 are maternal factors, 3 are components of E3 ligase, 7, 1, and 3 are components of Wnt, MES-1SRC-1, and Notch signaling pathways, respectively, 12 are transcription factors, and 8 are chromatin modifiers. We refer to the components of the first seven pathways in Fig5 as cell fate determinants because they are frequently involved in cell fate determination (Supplementary Table S8). Fifteen out of the remaining 17 genes function in various pathways, including mRNA production, protein modification, and trafficking or as actin-related protein, and the remaining two (F08D12.1 and F13H8.9) have yet been functionally characterized. The asynchrony between the precursor of germline and body-wall muscle cells (between “P4” and “D”), the biggest ADS of all sister pairs, is primarily dictated by maternal factors, components of the E3 ligase and the Wnt/Src signaling pathways but involves few of the remaining pathways. The extreme ADS between “P4” and “D” is likely due to the delayed transcription initiation in “P4” because previous findings have indicated that initiation of zygotic transcription in the germline is much delayed as against that in somatic cells, including the daughters of blastomere “D” (Mello *et al*, 1992; Seydoux & Dunn, 1997), which is dependent on the maternal factor, PIE-1 (Seydoux *et al*, 1996).

Genes that are identified across cell types or lineages with relative high frequency include the components of Wnt signaling pathway, chromatin modifiers, and a few maternal factors and uncharacterized genes, for example *mex-1, cul-1* and *tads-1*, whereas the remaining genes appeared to be cell specific. To our surprise, the cell-specific roles of Notch signaling in temporal regulation demonstrate a precise correlation spatially with its reported roles in cell fate specification (Fig5, pink columns). For example, three cells, ABalpaap, ABplpapp, and ABplaaap, were reported to receive Notch signaling for cell fate specification in various experiments, including cell ablation, mutant and gene expression analysis (Hutter & Schnabel, 2013; Moskowitz & Rothman, 1996; Priess, 2005). The same three cells were also subjected to regulation by Notch signaling for temporal coordination. In addition, the roles of Notch signaling in asynchrony appeared to be AB specific, which is in agreement with its specific roles in cell fate specification of “AB” lineage (Neves & Priess, 2005). The correlation between Notch's roles in temporal and fate regulation at the cellular level indicates a coupled regulation of cell fate specification and division timing, an important aspect that is commonly ignored during the study of cell fate differentiation. Establishing the exact relationship between the temporal regulation and cell fate specification will require further biochemical and/or cellular analyses, which are beyond the scope of this study.

Interestingly, our data demonstrated the role of Notch signaling in controlling ADS of ABplaaap (to develop into the part of left head) but not that of ABarpapp (to develop into the part of right head) although the two cells develop into symmetric cell types (Figs4A and 5). Consistent with this, Notch signaling has previously been reported to be required for inducing the fate of ABplaaap but not that of ABarpapp (Hutter & Schnabel, 1995; Moskowitz & Rothman, 1996), suggesting a coupled regulation of division asynchrony and fate asymmetry in the former cell by the Notch signaling pathway. As described earlier, the anterior and posterior daughters of the two cells become terminally differentiated and postembryonic blast cells, respectively. Only a single putative chromatin modifier, *cbp-2*, was identified for the right head development, whereas no chromatin modifier was identified for the left head development even though chromatin modifiers were found to be frequently involved in the asynchrony of other cells (Fig5). In contrast, the Wnt signaling pathway and transcription factors were frequently involved in controlling the ADS of both ABplaaap and ABarpapp, suggesting that the maintenance of cell differentiation status as a blast or terminally differentiated cell seems to primarily depend on Wnt signaling and transcription factors rather than chromatin modifiers.

### Coordination of cell division timing during tissue growth

It is intriguing that asynchrony is not only observed in divisions that produce daughters of different cell types, but is also found in those that give rise to the same cell type, that is, during tissue growth (Figs1C and 4A; Supplementary Fig S4A) (Sulston *et al*, 1983). The presence of asynchronies during tissue growth indicates that regulation of ADS and fate asymmetry is separable during metazoan development. To screen for genes involved in the regulation of division asynchrony during tissue growth, we focused on the ADS of a subset of 16 cells that are derived from various sublineages, but each of them develops into a single-cell type (Figs4A and 5 (right panel), Supplementary Fig S4A). The subset was prioritized based on the criteria similar to those for cell fate specification except that as many as possible single-cell types rather than a combination of cell types were included.

A total of 54 genes/pairs were reproducibly identified whose depletion produced a significant and over 50% reduction in the ADS of at least one cell compared with that of the wild-type average. Strikingly, 32 out of the 54 genes (59.3%) are also known to mediate cell fate specification directly or indirectly (Supplementary Table S8) though they are found to function primarily on temporal regulation in the 16 cells. In addition, 33 (61.1%) out of the 54 genes play dual roles in regulating the ADS during both tissue growth and fate specification. The overall pathways of the genes involved in temporal control are also similar between the two cellular processes. Taken together, breaking of division asynchrony during the proliferative stage of *C. elegans* embryogenesis primarily depends on cell fate determinants regardless of the fate contexts they are operating on, indicating that cell fate determinants do not only play a central role in establishing fate asymmetry, but also in setting division asynchrony during both tissue growth and fate differentiation.

Genes that are identified with relatively high frequency during both tissue growth and fate specification include components of Wnt pathway, the maternal factor MEX-1, the E3 ligase component CUL-1 as well as a few chromatin modifiers, for example, *cbp-1, cbp-2*, and *let-526*. *cbp-1* is required for widespread histone acetylation of Wnt target loci upon Wnt signaling induction (Parker *et al*, 2008), while *cbp-2* encodes a protein with an unknown function but its high sequence homology to CBP-1, suggests a role in histone acetylation. *let-526* encodes a component of the SWI/SNF complex that is involved in nucleosome remodeling required for the asymmetric division of tail seam cells during *C. elegans* postembryonic development (Shibata *et al*, 2012). The remaining genes appeared to be individually recruited by different cells to control the asynchrony between their daughters. For example, Notch components only regulate ADS in AB-derived precursors of pharyngeal tissue.

### Differential coordination of cell division timing during tissue growth and fate specification

Despite the striking similarity of the genes involved in regulating ADS during both fate specification and tissue growth, there are quite a few genes that show differential regulation of each category. Overall, the genes showing the most prominent differential coordination are transcription factors (Fig5). Only 5 out of the 17 identified transcription factors have a shared regulation of ADS in both categories, whereas 5 and 7 transcription factors are unique for tissue growth and fate specification, respectively. This is not surprising considering that transcription factors often function in a tissue-specific manner. Intriguingly, two Hox genes, *ceh-13* and *nob-1*, are found to be involved in regulating ADS during fate specification but not during tissue growth, which is consistent with the fact that Hox genes control cell fate specification in a position rather than tissue-dependent manner. However, the involvement of *nhr-25* during fate specification, namely breaking of fate asymmetry between neuron and hypodermis, but not during tissue growth is unexpected considering its role as a hypodermis-specific transcription factor (Shao *et al*, 1998). This result suggests that *nhr-25* could play a role in defining the hypodermis fate identity by regulating the division asynchrony of its ancestor. Chromatin modifiers also demonstrate differential regulation on temporal coordination. For example, *lex-1* and *lin-40* appear to be unique for cell fate specification, while *dpy-28, hat-1*, and *mys-2* seem to be specific for tissue growth (Fig5). One should be aware of the limitations arising from the thresholds or the tissue markers used in the screening. The genes identified for the differential control of ADS may vary when different thresholds or tissue markers are used.

It is worth noting that most of the identified genes that do not appear to be cell fate determinant demonstrate a differential regulation of ADS between the two categories except for *tads-1*, *ddx-23*, and *smo-1*. The exact mechanism of how these three genes are involved in asynchrony breaking remains to be determined. Interestingly, transcription factors show minimal involvement in regulating the ADS within body-wall muscle. In contrast, they demonstrate frequent involvement in breaking the fate symmetry between hypodermis and neuron from their common ancestor. Nevertheless, conclusive statistical support for this finding cannot be provided by the data available, and therefore, the differential involvement of transcription factors in the different processes remains to be further examined. A differential regulation of ADS is not only observed between fate specification and tissue growth, but also between tissue growths in the context of distinct fates. The control of overall ADS within the same cell fate seems more likely to share regulators than the control over ADS of another fate. For example, the hypodermis-specific transcription factors are rarely responsible for temporal coordination in body-wall muscle. On the other hand, the muscle-specific maternal factors are barely used in temporal coordination in hypodermis. Interestingly, MS-derived pharyngeal cells are more likely to share regulators than AB-derived pharyngeal cells. A similar situation appears to be true between blastomere “D-” and “C-” derived muscle cells, demonstrating that lineal origin plays an important role in “selecting” the cohort of regulatory proteins for ADS control.

### Roles of the identified genes in cell fate specification

To examine whether the genes identified in our screen play further roles in addition to the regulation of asynchrony, we focused on a subset of six genes whose depletion disrupted the ADS of the precursor of excretory cell, a functional equivalent of the vertebrate kidney, namely *ceh-43*, *sptf-3*, *tbx-33*, *let-526*, *snfc-5*, and *arx-1*. Division of the precursor of the excretory cell, ABplpapp, shows both fate asymmetry and asynchrony (Figs4A and 5). All six genes encode a transcription factor or chromatin modifier except *arx-1*. We selected these genes based on the following criteria. First, they are potential cell fate determinants expected to produce defects in fate specification upon perturbation. Second, they tend not to produce dramatic defects in cell migration such as gastrulation failure upon inactivation. One gene, *arx-1*, that does not appear to encode a cell fate determinant, was included because of its functional relevance to excretory cell development in *C. elegans* (Sawa *et al*, 2003).

**Figure 6:**
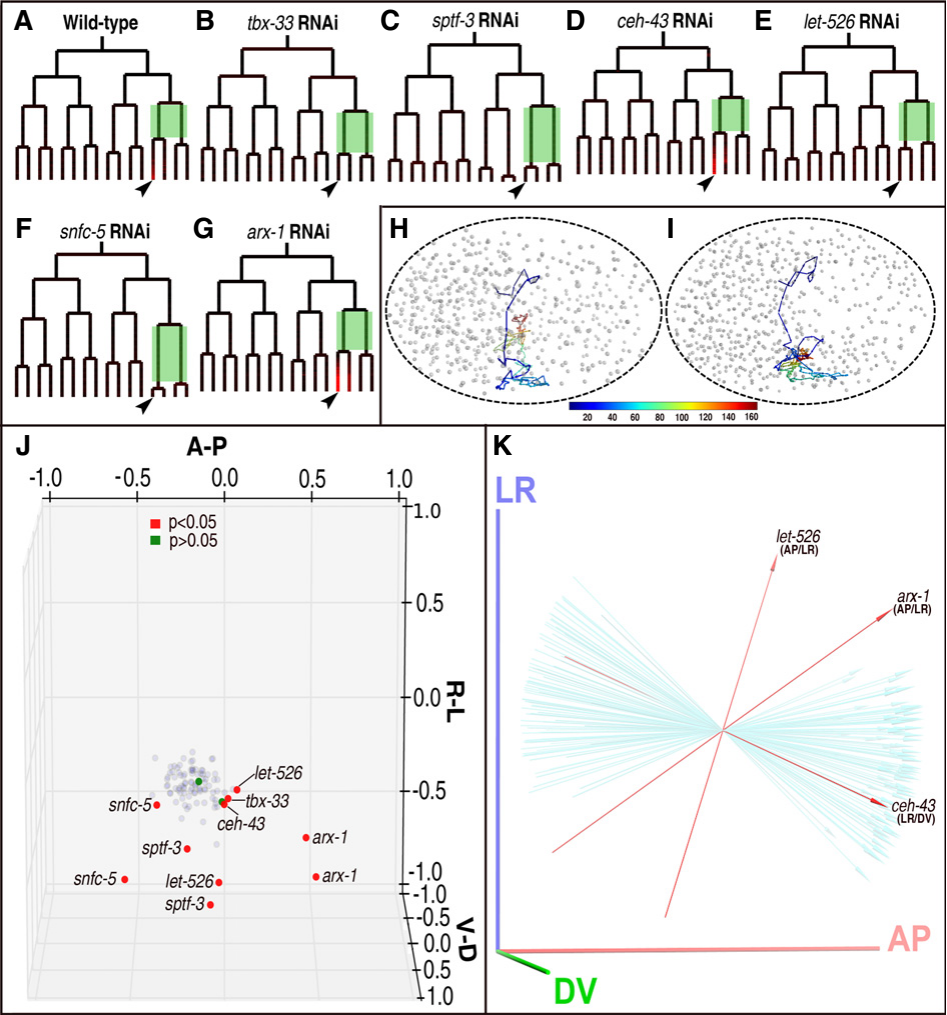
Roles of temporally relevant genes in regulation of fate specification and cell migration using excretory cell precursor as an example

To examine the role of the six genes in excretory cell fate specification, we analyzed the lineal expression of an excretory cell-specific marker, CEH-26, before and after inactivation of these genes during embryogenesis. CEH-26 is a vertebrate Prox1 homologue that is specifically expressed in the precursor of the excretory cell prior to 350-cell stage of embryogenesis (Supplementary Fig S7). It has been shown to regulate the expression of several factors that mediate the excretory lumen extension (Kolotuev *et al*, 2013). Inactivation of four (*tbx-33*, *sptf-3*, *let-526*, and *snfc-5*) out of the six genes not only significantly reduced the division asynchrony of ABplpapp, but also eliminated the CEH-26 expression (Fig6A–C, E and F), suggesting a coupled regulation of division timing and excretory cell fate specification. Inactivation of the remaining two genes significantly reduced the asynchrony but produced few changes in CEH-26 expression, suggesting separate regulation of the asynchrony and excretory cell fate specification. Further evidence of a coupled regulation of division asynchrony and cell fate specification comes from Notching pathway, which has previously been shown to be required for the excretory cell specification (Hutter & Schnabel, 1995; Moskowitz & Rothman, 1996). In addition to its role in regulating excretory cell fate specification, inactivation of two Notch components, LAG-1 or SEL-8, produced defective ADS of ABplpapp, indicating a role of the pathway in both temporal coordination and cell fate specification of the excretory cell. Examination of the lineal expression of another tissue marker, NHR-25 that is specific for hypodermis, also demonstrated a coupled regulation of both hypodermis fate specification and ADS in ABarpap by the Wnt signaling pathway and transcription factors (Fig5; Supplementary Fig S8). For example, depletion of two Wnt pathway components, POP-1 and LIT-1, eliminated NHR-25 expression, while a similar depletion of three transcription factors, EGL-18, HAM-1, and TBX-33, reduced the expression of NHR-25 in the daughters of ABarpap. Taken together, the regulation of division asynchrony seems to be either coupled or distinct from the regulation of fate specification.

A–G Lineal expression of CEH-26 (depicted in red) in wild-type (A) and RNAi (B–G) embryos with genotypes indicated on the top. All trees are rooted with ABplpa. The arrowheads indicate ABplpappaa, that is, the mother of excretory cell. Two sister cells used for calculation of ADS are shaded in green. Note the abolishment of CEH-26 expression by RNAi against *tbx-33*, *sptf-3*, *let-526*, and *snfc-5* but not by RNAi against *ceh-43* and *arx-1*.

H, I 4D trajectories showing migration of the excretory cell precursor starting from ABp up to its final differentiation into ABplpappaap at approximately 550-cell stage for a wild-type and *let-526* RNAi embryos, respectively. Shown are the ventral views of the embryos with anterior to the left. The cell migration path is depicted starting from the last time point of ABa so that it changes color with accumulative developing time. Color coding of the time scale in minutes is shown at the bottom.

J The relative positions of ABplpappa at its last time point (approximately 350-cell stage) for wild-type (gray dots) and perturbed embryos (green or red dots with gene name indicated) with two replicates each. Green and red dots denote deviations from wild-type distribution with *P* > 0.05 and *P* < 0.05, respectively (see Materials and Methods). Embryos are shown with anterior to the left and right to the top. A-P, L-R, and D-V depict anterior–posterior, left–right, and dorsal–ventral axes respectively. Dot is not drawn to scale in size.

K Shown are division angles of 91 wild-type (light blue) and three perturbed embryos with genes indicated (red). Division angles are calculated as that between the division orientation of ABplpappa relative to the three planes defined by axes between AP/LR, AP/DV, and LR/DV. Only division angles that are significantly deviated from wild type upon perturbation are shown. The reference plane for calculation of the angle deviation is indicated below gene name. Embryo axes are defined in the same way as that in (J).

### Roles of the identified genes in cell migration and tissue morphogenesis

The precursor of the excretory cell has undergone a complicated and long-range migration before its differentiation into the terminal fate (Fig6H; Supplementary Movie S2). Inactivating two of the six genes mentioned above, *let-526* and *ceh-43,* led to a defective migration of the excretory cell precursor (Fig6I; Supplementary Movie S3). To further evaluate the roles of the six genes in cell migration during embryogenesis, we examined the relative positions of ABplpappa, the grandmother of the excretory cell, at its last time point in the context of multiple wild-type embryos (see Materials and Methods). Inactivating four out of the six genes, that is, *sptf-3*, *let-526*, *snfc-5*, and *arx-1*, produced a significant and reproducible shift in its positions relative to those of the wild type (*P* < 0.05) (Fig6J). Inactivation of the remaining two genes, *tbx-33* and *ceh-43*, produced a significant shift in only one replicate but not in the remaining replicate embryo, probably due to incomplete penetrance of the RNAi. In addition, depletion *let-526*, *arx-1*, and *ceh-43* produced significant deviation in division angles relative to a plane defined by AP/LR, LR/DV, or AP/DV, respectively, from those of the wild-type embryos (Fig6K). Inactivation of the remaining three genes produced insignificant deviation in division angles though the relative positions of ABplpappa in most perturbed embryos were significantly shifted compared to the wild-type embryos (Fig6J; Supplementary Fig S9A–H). The results suggested that the control over asynchrony might serve at least partially to coordinate cell migrations for proper tissue growth.

Perturbation of the temporal coordination during an early developmental stage could be manifested as a defect in morphogenesis at a later developmental stage. To test this hypothesis, we evaluated the roles of the six genes during excretory morphogenesis post-embryonically. Specifically, we injected the dsRNA derived from each gene into the worm expressing a chromosomally integrated marker, that is, *pgp-12*::GFP, which is exclusively expressed in the excretory cell post-embryonically (Zhao *et al*, 2005). Examining the morphological defects in excretory canals revealed that all of the surviving L1 larvae showed various defects in the excretory canals (Supplementary Fig S9I–P). RNAi against *snfc-5* also produced an extra nucleus, suggesting a defect in fate specification of the excretory cell. RNAi against *tbx-33* and *ceh-43* produced canals with a “thorn”-like protrusion. Inactivation of *let-526* and *arx-1* led to incomplete canals while that of *sptf-3* seemed to affect tube morphogenesis. RNAi against most of these genes (i.e. *sptf-3*) induced a high penetrance of embryonic lethality. Therefore, the morphological changes in the excretory canal should be treated as a hypomorphic rather than a null phenotype. The results demonstrate that a defect in asynchrony during an early developmental stage could be manifested as a defect in morphogenesis during a later developmental stage.

## Discussion

**Figure 7:**
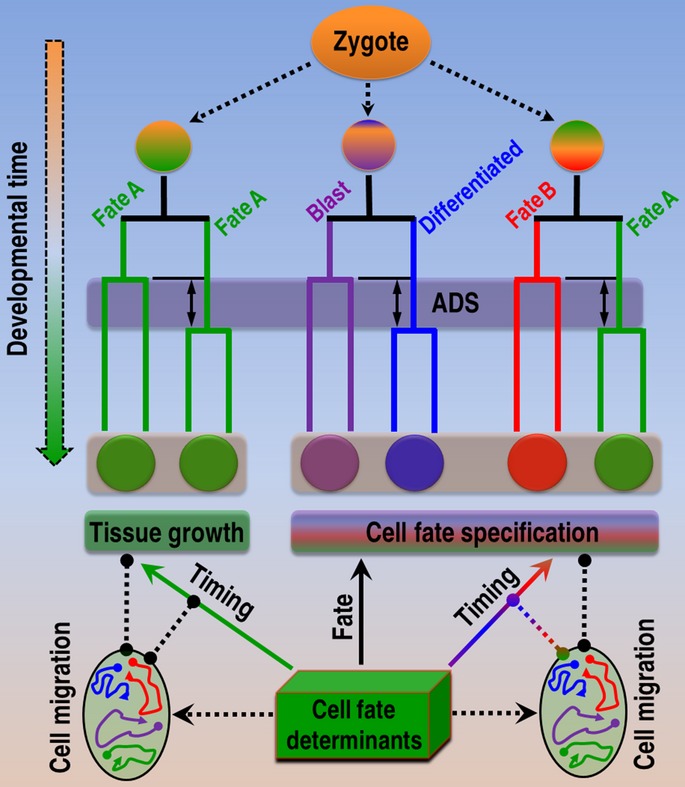
A model of temporal coordination during metazoan development The division asynchrony is present during both tissue growth when all daughters develop into a single-cell type (green branches) and cell fate specification when two daughters develop into distinct cell types (differentially color-coded branches). Cell fate determinants play a major role not only in establishing fate asymmetry (black arrow), but also in achieving division asynchrony in association with (rainbow arrow) or independent of (green arrow) fate specification. Differential control over division pace appears to coordinate cell migrations (dashed black arrows) to facilitate both fate specification and tissue growth. Possible genetic interactions between cellular processes with uncertain directionality are indicated with dot-ended dash lines.

During the early stages of metazoan embryogenesis, temporal coordination of cell division is crucial for proper cell fate specification and tissue growth, and it has remained largely unknown how this coordination is genetically regulated *in vivo* at cellular resolution. Here, we systematically characterized the genetic control over the asynchrony of cell division during *C. elegans* embryogenesis using a combination of RNAi and automated lineaging. We found that most of the genes involved in the temporal regulation are those that are commonly required for cell fate specification, indicating that metazoan species use a common regulatory architecture for establishing both fate asymmetry and division asynchrony during the proliferative stage of embryogenesis (Fig7). Regulation of division asynchrony during tissue growth may partially serve to determine the total rounds of cell division to control tissue size, whereas the regulation of division asynchrony during fate specification may mostly serve to coordinate cell migration. The regulatory factors identified in our screen provide an entry point for establishing a mechanistic understanding of how fate asymmetry and division asynchrony are coordinated at cellular resolution during metazoan development. Given that numerous chromatin modifiers are found responsible for the division asynchrony between or within cell type(s) (Fig5), it is tempting to speculate that these factors may differentially regulate the chromatin status between two daughter cells, which could selectively activating the cell cycle components such as those involved in gap introduction or progression of DNA replication (Brauchle *et al*, 2003). Future studies on cell-specific expression and/or chromatin immunoprecipitation followed by next-generation sequencing (ChIP-seq) could elucidate the exact relationship between the regulation of division asynchrony and fate asymmetry.

Some of the genes identified in our screen may simultaneously regulate the asynchrony and fate asymmetry, whereas some others may function solely to establish the asynchrony in one cell type, but serve to break the fate symmetry in another cell type. For instance, we observed that a *Drosophila* GRainyHead homolog, *grh-1*, was required for the ADS of MSaapa, which develops into pharyngeal tissue (Fig5). However, the gene was also required for the synthesis of cuticle, a derivative of hypodermis (Venkatesan *et al*, 2003), demonstrating that the gene can be recruited for either fate specification or temporal coordination depending on its cellular context. A predominant role of cell fate determinants in regulating ADS during tissue growth is particularly intriguing. This is because an asymmetric segregation or expression of cell fate determinants is often responsible for a fate asymmetry, but the genetic control over division asynchrony during tissue growth of metazoan development remains largely unknown though it has long been observed. Apparently, metazoan development demands not only fate specification, but also division-pace coordination between dividing cells, which may function to facilitate cell migration (Fig7). For example, body-wall muscle cells are derived from different lineal origins, including blastomeres “MS”, “C,” and “D”, whose daughters are intermingled with one another to form a seamless muscle tissue (Supplementary Fig S10A). A coordination of division pace between different lineal origins would be essential for the accurate formation of the muscle tissue. Consistent with this, inactivation of TADS-1, a protein with unknown function, not only led to a disruption of ADS in muscle precursors, but also produced apparent defects in cell migration (Supplementary Fig S10B and C), which is likely to disrupt the spatial alignment of body-wall-muscle precursors derived from different blastomeres.

Our data demonstrate that cell fate determinants and cell cycle components appear to play an active and passive role, respectively, in cell-specific regulation of division asynchrony. Consistent with this, 31 out of the 822 genes were annotated as a putative component of cell cycle control based on their sequence homology or reported roles (Supplementary Table S9). However, only one of them, *cdk-8*, was found to be involved in the regulation of ADS during tissue growth (Fig5). Notably, the gene is orthologous to human CDK8 that is part of the mediator complex associated with RNA polymerase II holoenzyme (Tsai *et al*, 2013), suggesting its role in ADS could be achieved by mediating interaction between cell fate determinants and RNA polymerase II. As mentioned above, it remains possible that the regulatory factors identified in our screening may control the division asynchrony by regulating the differential expression of cell cycle components between sister cells. In agreement with this, inactivation of quite a few cell cycle components tends to affect global division pace. These genes are not expected to be picked up in our screening because we only screened for the defect in relative cell cycle length between the two sisters of the same embryo rather than the absolute change in cell cycle length between wild-type and perturbed embryos. For example, inactivation of five cyclin-encoding genes, *cyl-1, cyh-1, cye-1, cyb-1*, and *cyd-1*, caused an overall deceleration in development or early embryonic arrest, except for *cyd-1*, which is dispensable for early embryogenesis as reported previously (Supplementary Table S9, our online database) (Boxem & van den Heuvel, 2001; Yanowitz & Fire, 2005). Inactivation of several other cell cycle components, for example *cdk-1*, *chk-1* (a DNA check-point kinase), *wee-1.3* (a kinase of Wee 1 family), and *air-2* (a gene encoding an Aurora-like protein), led to an early embryonic arrest (Supplementary Table S1 and Supplementary Table S7), which prevents a thorough interpretation of their roles in establishing cell fate asymmetry and division asynchrony. A more detailed analysis is required to elucidate the role of these cell cycle components in temporal regulation, for example, by conditional or cell-specific mutation or partial knockdown.

In addition to the identification of the genetic components required for cell-specific division asynchrony, our data also holds potential for providing insight into the biochemical function of uncharacterized genes or for inferring cell type-specific regulatory pathways. For example, inactivation of *repo-1* led to a systematic depletion of lineaging marker expression (Supplementary Fig S11), supporting its role as a putative splicing factor as inferred from sequence homology. A subset of genes involved in the regulation of cell-specific division asynchrony (Fig5) could be explored for inferring the gene regulatory pathway controlling fate specification or tissue growth of the cell. For instance, a putative such pathway for excretory cell specification could be constructed based on a combination of the genes identified in our study with the existing functional data defined in STRING (version 9.1) (Supplementary Fig S12), which establishes gene interaction with known and predicted evidences (Franceschini *et al*, 2013). This may serve as an entry point for validating the relevant genes in excretory cell specification because the functional interactions between these genes reported in the STRING database may not hold if applied at cellular resolution for the excretory cell. A similar network could be constructed for any other cell type using the cell-specific regulatory factors identified in this study. One of the major challenges in cancer biology is the difficulty in diagnosing the cell-specific origin of an observed malignancy. Given the conservation of the identified genes, various regulatory factors identified in our screening could provide insights into the tissue-specific origin of a cancerous cell. The genes that frequently regulate ADS during fate specification or tissue growth could become a promising target for anti-cancer therapy.

To facilitate public access to the quantitative data generated in this study, we have developed a web-accessible database called “Phenics” (phenotyping of *C. elegans* embryo with single-cell resolution), which is available at http://phenics.icts.hkbu.edu.hk/. It hosts the readouts from our screening pipeline, including a pairwise comparison of cell cycle lengths between a wild-type embryo and a perturbed embryo in different formats, for example, as a dot plot, a histogram or a bar graph. It also contains a pairwise comparison of developmental speed (calculated as the total cell count over time) between a wild-type embryo and a perturbed embryo as well as a lineage tree with superimposed tissue-marker expression for each perturbed embryo. The database provides a downloadable 3D time-lapse movie reconstructed from space-filling model for each perturbation, which provides a qualitative view of cell migrations. The phenotypes on division timing can be queried by RNAi-targeting gene name, edited cell number or last edited time point or ranked by various tabs on the top of each page. Cell-specific absolute cell cycle length or ADS can be queried through a tab called “Single-Cell Division timing” or “Sister Cell Division timing,” respectively.

## Materials and Methods

### Worm strains and growth

All the animals were maintained on NGM plate seeded with OP50 at room temperature. The genotypes of the strains used in lineaging are listed in Supplementary Table S3. In addition to the lineaging strains, the transgene carrying a promoter fusion, *pgp-12*::GFP, in the strain BC10210 was used as a postembryonic marker for the excretory cell with genotype of *dpy-5*(e907) I; *sIs*10089 [rCes *pgp-12*p::GFP + pCeh361].

### Gene prioritization for RNAi

The following filters were used to prioritize genes to be included in our screening. First, they were annotated to produce embryonic lethality or larval arrest upon perturbation by RNAi or genetic mutation in Wormbase (Yook *et al*, 2012). Second, they were required to bear an unambiguous human ortholog as annotated in OrthoList (Shaye & Greenwald, 2011). Applying a combination of the two filters gave rise to 1,948 unique protein-coding genes (Supplementary Fig S1A). Third, to increase the likelihood of capturing embryo-nic phenotypes, only those whose transcripts had been shown to be enriched by at least two folds in embryo relative to larval and adult stages (Gerstein *et al*, 2010) were retained. Finally, the genes reported to produce early embryonic arrest at a stage of a handful of cells or to be defective in cytokinesis upon perturbation were manually removed. As a result, 822 genes were chosen for the subsequent screening (Supplementary Table S1).

### dsRNA production and RNAi

Gene knockdown was performed by RNAi through microinjection. Primers for amplification of dsRNA template were selected based on the similar criteria as described previously (Sonnichsen *et al*, 2005; Green *et al*, 2011) with the following modifications (Supplementary Fig S1B). First, in order to reduce potential cross inhibition, the sequences to be amplified are required to have less than 80% identity in any of its alignment of 50 bps or longer in size against exonic regions of the targeting gene; second, the primers were picked such that their TM values centered around 55°C and the predicted amplicons contained as much coding region of a target gene as possible. The amplicon size was demanded to be bigger than 200 bps but smaller than 1.0 kbps to facilitate annealing of dsRNAs in 96-well format. For dsRNA production, T7 promoter with the sequence TTTCCAGGTTGGGATCTCGGTGTTG was included at the 5′ ends of both forward and reverse primers. PCR was performed using *C. elegans* N2 genomic DNA as a template in 20 μl volume with the following cycling conditions: 95°C for 5 min, 95°C for 30 s, 55°C for 30 s, and 72°C for 90 s for 30 cycles, which was followed by a 7-min incubation at 72°C using *ExTaq* DNA polymerase (Clontech). PCR product was examined on a 1% agarose gel before its use in dsRNA production. Primers that failed in producing an amplicon with the expected size were repeated one time for PCR amplification. If the amplification failed again, the primers were discarded and an alternative primer pair was selected. One μl of the PCR product was used as a template for dsRNA production with NEB HiScribe T7 Quick High Yield RNA Synthesis kit according to the manufacturer's description. For annealing of dsRNAs, the reaction mixture was incubated at 75°C for 15 min in a water bath followed by turning off the heating power and incubating overnight in the same water bath. The annealed dsRNAs were examined on a 1% agarose gel for estimation of its concentration and size. Only those with expected sizes were used for preparation of injection mixture as follows. The dsRNA was diluted to a concentration of 100 ng/μl in TE buffer for microinjection. Control RNAi experiment was performed by injecting TE buffer only into the lineaging strain. A total of 20 embryos from the control RNAi were subjected to automated lineaging. No significant differences were observed compared to wild-type un-injected embryos in term of cell division timing and marker expression. Most RNAi experiments were performed for one gene a time with a few exceptions, including *tbx-8/tbx-9, tbx-37/tbx-38,* and *lin-12/glp-1*, for which their functional redundancies were well known and dsRNAs were mixed together at the same concentration for each gene as that for individual dsRNA before injection. Despite our efforts to maximize the specificity for the RNAi, cross inhibition may be unavoidable in some cases. On the other hand, because of the intrinsic incomplete penetrance of the RNAi technique, some of the genes may function in regulating ADS but could be missed in our screening due to either incomplete or irreproducible penetrance.

### Imaging for automated lineaging

One- to four-celled embryos were retrieved from the adults that had been subjected to injection for at least 12 h but no longer than 24 h. Embryos were mounted for imaging as described (Murray *et al*, 2012). Imaging was performed with an inverted Leica SP5 confocal microscope equipped with two hybrid detectors at a constant ambient temperature of 20°C. Images were consecutively collected for both GFP and mCherry channels with a frame size of 712X512 pixels and scanning speed of 800 Hz using a water immersion objective. The excitation laser beams used for GFP and mCherry are 488 and 594 nm, respectively. mCherry was used as a lineaging marker, whereas PHA-4::GFP as a tissue marker except for a few cases, where other tissue markers were used (Supplementary Table S1 and Supplementary Table S3). Fluorescence images from 41 focal planes were collected consecutively for three embryos per imaging session with a Z resolution of 0.71 μm from top to bottom of the embryo for every time point, which was one-and-a-half minutes. Images were continuously collected for a total of 240 time points during which the cell count would reach approximately 550 in a wild-type embryo. The entire imaging duration was divided into four time blocks by time point, that is, 1–90, 51–130, 131–200, and 201–240. Z axis compensation was 0.5–3% for the 488 laser and 20–95% for the 594 laser. The pinhole sizes for the four blocks were 1.6, 1.4, 1.0, and 0.8 AU (area unit), respectively.

### Automated lineaging, profiling of tissue-marker expression, and manual curation of the automated output

For automated lineaging, a custom Java script was written to convert and rename the raw images produced by Leica SP5 into StarryNite compatible formats. Automated lineaging and gene expression profiling were performed as described (Murray *et al*, 2012, 2012). Specifically, raw fluorescence TIFF images acquired for lineaging marker were used as an input for lineaging algorithms (Bao *et al*, 2006; Santella *et al*, 2010) implemented on Linux operating system to automatically reconstruct cell lineage. Subsequently, raw fluorescence TIFF images acquired for tissue marker were used as an input for computing the intensity of the tissue-marker expression for each cell with time using Acebatch as described (Murray *et al*, 2013). The output of the automated lineaging was manually curated to correct StarryNite errors in a graphical interface, AceTree (Boyle *et al*, 2006). At least two embryos were curated for each gene up to approximately 350-cell stage or up to the last time point that an embryo was editable in a perturbed embryo. Automated expression profiling was conducted again after the manual curation was completed. To generate lineal expression of lineaging marker (Supplementary Fig S11), instead of the raw TIFF images acquired from tissue marker, those acquired from the lineaging marker (H2B::mCherry, H3.3::mCherry) were used as an input for Acebatch.

### Gene ontology (GO) analysis of the screened genes

Functional enrichment analysis was performed using DAVID v6.7 (da Huang *et al*, 2009) with default parameters using the 822 genes screened in our pipeline as an input. Functional group with significant enrichment (*P* < 0.01) was retained for downstream analysis. Redundant categories of GO terms were manually removed.

### Quantification of cell division timings

Cell cycle lengths for all cells were computed from the output of lineaging pipeline and converted into minute. Since an arbitrary cutoff time point was applied to each embryo, cell cycle length for the cells that divided after the cutoff time point was excluded in the subsequent analysis. ADS of all the remaining cells (except that of ABa, ABp, EMS and P2) was computed for 91 wild-type and all the perturbed embryos. To evaluate the reproducibility of cell cycle lengths between the 91 wild-type embryos, mutual Pearson correlation coefficient (r) of all cell cycle lengths between individual embryos was computed with R (http://www.r-project.org/). Clustering analysis was performed with “heatmap.2” function in “gplots” package by using the complete “Manhattan” distance measure (Fig4B).

To identify the genes responsible for significant reduction in ADS between perturbed and wild-type embryos, embryos that did not satisfy the following criteria were excluded in the downstream analysis, namely the perturbed embryos that were not able to develop into 300 cells at its last editable time point or could not reach 350 cells up to 240 time point after curation. These embryos were assumed to develop in an “abnormal” or overall slowing speed. To identify genes involved in the regulation of ADS, genes whose depletion produced a significant reduction in the asynchrony in at least one pair of sister cells between the wild-type and perturbed embryos (*P* < 0.05) were retained for the subsequent analysis. To further increase the specificity of the screening, ADS of the perturbed embryo was demanded to be decreased by at least 50% than that of the wild-type embryos. In addition, only the genes with at least two replicates that simultaneously satisfied the above criteria were included as the genes that control division asynchrony (Fig5). To examine the statistical significance of the difference in ADS between wild-type and perturbed embryos with a relatively small sample size in perturbed embryos (usually two curated embryos per gene), D'Agostino's K-squared test was performed as described previously (Moore *et al*, 2013) to evaluate the distribution of ADS of individual cells between 91 wild-type embryos. At least 75.8% of all examined ADS values passed the normality test with an alpha value of 0.05, which allowed us to assign the probability of ADS of a perturbed embryo outside the 95% and 99% confidence interval of the distribution of wild-type ADS as the *P*-value, that is, *P* < 0.05 and *P* < 0.01, respectively. A *P*-value of 0.01 or 0.05 was assigned for Fig5 if the ADS of at least two perturbed embryos was significantly smaller than that of the wild-type embryos with *P* < 0.01 and *P* < 0.05, respectively (see Supplementary Table S10).

To identify the genes with the similar buffering effect in division timing as *cbp-1*, we first calculated the dispersion in cell cycle length of “AB” progeny for both wild-type and perturbed embryos, which is defined as the absolute deviation in cell cycle length of an individual embryo from the wild-type average cell cycle lengths in the same generation for either individual wild-type embryo or perturbed embryo. We next evaluated the significance level in difference of the dispersion between the perturbed and wild-type embryos by comparing the dispersions of all perturbed and wild-type embryos for a given generation using two-sample *F*-test. A gene with *P* < 0.01 was retained (Supplementary Fig S2).

### Quantification of cell migrations

3D space-filling model was generated using the StarryNite output as an input. To aid visual comparison, the axes of embryos were normalized so that the midpoint between “ABa” and “P2” at the last time point of the four-cell stage was used to define A-P (anterior–posterior) axis and that between “ABp” and “EMS” to define L-R (left-right) axis. The remaining dimension was used to define the D-V (dorsal–ventral) axis. All the 3D graphs and the projection movies were normalized to the same orientation, that is, anterior to the left and right to the top. Projection movies were generated as follows. The curated coordinates of cell nuclei were first converted into a text file that was compatible with UCSF Chimera's. bld format (Pettersen *et al*, 2004), which was then rendered into TIFF images using Persistence of Vision Raytracer (POV-ray, www.povray.org). Finally, the generated images were converted into movies using FFmpeg (www.ffmpeg.org). The founder cells in movie were differentially color-coded as follows unless stated otherwise: “ABa”: red, “ABp”: blue, “C”: magenta, “D”: pink, “E”: green, “MS”: cyan, and “P2”: yellow.

To facilitate statistical comparison of cell positions across embryos, all the embryos were aligned temporally at the last time point of four-cell stage. In addition, embryos were normalized to the same sizes ranging from −1.0 to 1.0 in all dimensions. To evaluate the variability of the spatial migration, normality test was conducted on the spatial distribution of the cell of interest across 91 wild-type embryos using the D'Agostino's K-squared test. A total of 72.2% of the values passed the test when an alpha value of 0.05 was used. The centroid of the wild-type distribution was computed. Significant deviation from wild-type distribution for the cell migration of a perturbed embryo (defined as distance to the wild-type centroid) was assigned in the similar way as that for ADS, that is, the probability that fall outside the 95% confidence interval of the wild-type distribution.

The trajectory was drawn by first tracing the lineal origin of a cell from its “root,” which is one of “ABa”, “ABp”, “P2,” and “EMS”. For each time point, an arrow was drawn from the starting to the end coordinate of the traced nuclei. The result was a trajectory composed of many small arrows colored over time, with the trajectory tail in blue depicting the first and the trajectory head in red showing the last curated time point of the cell of interest, respectively. Therefore, the size of the arrow corresponded to cell migration pace. Trajectory movie was generated in the same way as that for 3D projection movie.

Statistical analysis of division angles was performed as follows. Division angle of a single cell is defined as that between the line formed by the two daughters immediately after division against three reference planes, that is, AP-LR, AP-DV, and LR-DV, respectively. A close examination on their distributions across wild-type embryos revealed random deviations from normal distribution, in particular for the cells which are located near the egg-shell. To accommodate this issue, we performed statistical transformation on the wild-type division angles using Box–Cox transformation (Box & Cox, 1964). The power parameter λ was determined using maximum-likelihood estimation (MLE). Finally, the transformed data were further de-noised by removing outliers, which was defined as the instances that were at least 3 standard deviations from the mean. We tested the transformed data for normality using D'agostino's K-squared test. With a cutoff of α = 0.05, the ratio of normally distributed angles were 93.6, 84.2, and 75.1% against planes AP-LR, LR-DV, and AP-DV, respectively. The means and standard deviations were computed for the distribution of the angles of wild-type embryos. Significant deviation (*P*-value) from wild type was computed in the similar way as that for the positions.

### Data availability

All the supplementary figures, tables and movies are provided as supplementary files. More detailed information on cell division timing, gene expression, cell migration, and RNAi can be found in the online database “Phenics,” which is available at: http://phenics.icts.hkbu.edu.hk/.

## Supporting Information

Supplementary Movie S3

Review Process File
