# Supplementary material for: Systems-level quantification of division timing reveals a common genetic architecture controlling asynchrony and fate asymmetry
Source: Mol Syst Biol. 2015 Jun 15;11(6):814. doi: 10.15252/msb.20145857 (PMC4501849; doi:10.15252/msb.20145857)
Supplement: Supplementary file 1 [file msb0011-0814-sd1.pdf]

Systems-level quantification of division timing reveals a common genetic architecture controlling asynchrony and fate asymmetry

Vincy Wing Sze Ho[1*], Ming-Kin Wong[1*], Xiaomeng An[1*], Daogang Guan[1*], Jiaofang Shao[1], Hon Chun Kaoru Ng[1], Xiaoliang Ren[1], Kan He[1,2], Jinyue Liao[3], Yingjin Ang[3], Long Chen[4], Xiaotai Huang[4], Bin Yan[1], Yiji Xia[1], Leanne Lai Hang Chan[4], King Lau Chow[3], Hong Yan[4] & Zhongying Zhao[1,5,#]

[1]Department of Biology, Hong Kong Baptist University, Hong Kong; [2]Center for Stem Cell and Translational Medicine, School of Life Sciences, Anhui University, Hefei, China; [3] Division of Life Science and Division of Biomedical Engineering, the Hong Kong University of Science and Technology, Hong Kong; [4]Department of Electronic Engineering, City University of Hong Kong, Hong Kong; [5]State Key Laboratory of Environmental and Biological Analysis, Hong Kong Baptist University, Hong Kong, China

[*]These authors contributed equally to this work

[#]Corresponding author

Supplementary movie legends

Supplementary Movie 1. A 3D projection movie (ventral view with anterior to the left) produced with raw 4D images showing embryogenesis of a wild-type developing embryo from 4-cell to comma stage. The red 3D projection was generated with fluorescence micrographs of the lineaging markers while the green 3D projection was built from those of PHA-4::GFP.

Supplementary Movie 2. A trajectory movie showing migration of the excretory cell precursor in a developing wild-type embryo. The trajectory starts from "ABp" and ends around 550 cells. Embryo orientation and trajectory color scheme is the same as that in Fig 6H respectively.

Supplementary Movie 3. A trajectory movie similar to the movie 2 showing the migration of the excretory cell precursor in a developing embryo treated with *ceh-43* RNAi.

Supplementary tables

Supplementary Table S1. List of the genes that were included in our pipeline (attached as a separate Excel file)

Supplementary Table S2. Gene ontology analysis of the genes that were screened in our pipeline

GO Term	Gene count	%*	p value	Fold Enrichment
Larval development	459	53.6	6.92E-111	2.51
Regulation of growth	413	48.2	9.91E-63	2.02
Reproductive developmental process	245	28.6	9.36E-61	2.84
Sex differentiation	233	27.2	1.38E-59	2.91
Regulation of transcription	141	16.5	2.42E-10	1.66
Multicellular organism reproduction	106	12.4	3.59E-16	2.24
Tissue morphogenesis	103	12.0	1.93E-24	2.88
Regulation of multicellular organism growth	85	9.9	5.73E-10	1.97
Cell cycle	75	8.8	4.27E-11	2.20
Reproductive behavior	70	8.2	3.10E-11	2.29
RNA processing	62	7.2	3.85E-20	3.60
Embryonic morphogenesis	60	7.0	3.11E-23	4.16
Protein localization	60	7.0	1.47E-05	1.76
Regulation of vulval development	59	6.9	3.97E-20	3.74
Sexual reproduction	57	6.7	2.41E-10	2.43
Molting cycle	52	6.1	1.13E-06	2.01
Aging	45	5.3	1.43E-04	1.79
Protein transport	45	5.3	2.09E-04	1.76
Gastrulation	42	4.9	7.40E-18	4.50
Gamete generation	42	4.9	2.87E-07	2.33
Chromosome organization	38	4.4	5.85E-08	2.60
Gonad development	37	4.3	1.25E-12	3.72
Cytoskeleton organization	37	4.3	1.01E-05	2.16
Regulation of transcription from RNA polymerase II promoter	33	3.9	2.00E-11	3.74
Organelle localization	27	3.2	2.81E-07	3.03
RNA splicing	23	2.7	2.10E-11	5.16
Cell migration	23	2.7	2.45E-06	3.04
ncRNA metabolic process	23	2.7	0.004	1.88
Spindle organization	22	2.6	7.36E-06	2.95
Ribosome biogenesis	21	2.5	3.71E-08	4.05
Cuticle development	21	2.5	8.38E-04	2.22

GO: gene ontology. Only functional class that contains over 20 genes and shows significant enrichment with $p<0.01$ is shown**.** ***** Percentage of the genes in current GO category out of the total number of input genes

Supplementary Table S3. List of lineaging strains and its genotypes

Strain name	Genotype	Tissue marker	Tissue of interest
RW10425	*unc-119(ed3)* III; *stIs10116 [his-72(promoter)::his-24::mCherry::let-858 3'UTR + unc-119(+)]; stIs37 [pie-1(promoter)::mCherry::H2B::pie-1 3'UTR + unc-119(+)]; stIs10389 [PHA-4::TGF(3E3)::GFP::TY1::3xFLAG]*	PHA-4	Pharynx and intestine
RW10481	*unc-119(ed3)* III*; stIs10116[his-72(promoter)::his-24::mCherry::let-858 3'UTR + unc-119(+)]; itIs37 [pie-1p::mCherry::H2B::pie-1 3'UTR + unc-119(+)]. stIs10436 [hlh-1::TGF(6.2B4)::GFP::TY1::3xFLAG]*	HLH-1	Body wall muscle
RW10348	*unc-119(ed3)* III*; stIs10116 [his-72(promoter)::his-24::mCherry::let-858 3'UTR + unc-119(+)]; stIs37 [pie-1(promoter)::mCherry::H2B::pie-1 3'UTR + unc-119(+)]; stIs10318 [NHR-25::TGF(3H4)::GFP::TY1::3xFLAG]*	NHR-25	Hypodermis
RW10913	*unc-119(ed3)* III*; stIs10116 [his-72(promoter)::his-24::mCherry::let-858 3'UTR + unc-119(+)]; stIs37 [pie-1(promoter)::mCherry::H2B::pie-1 3'UTR + unc-119(+)]; stIs10703 [CEH-26::TGF(3H4)::GFP::TY1::3xFLAG]*	CEH-26	Excretory cell

Supplementary Table S4. Average cell division timings, ADS and accumulative division timing derived from 91 wild-type embryos (attached as a separate Excel file)

Supplementary Table S5. Comparison of fate transformation between those observed in this screening and those reported by Du et al., 2014.

Gene	Phenotypes reported by Du et al., 2014	Phenotypes in this study: Observed	Phenotypes in this study: Uncertain	Comments
apx-1	ABp→ABa		✓	
gld-2	ABar→ABal		✓	
	ABpra→ABpla		✓	
	E→MS		✓	
	P_4→D		✓	
*glp-1 /lin-12**	ABp→ABa		✓	PHA-4 expression is lost in most AB lineages.
	ABalp→ABarp		✓	
	ABara→ABala		✓	
	ABpla→ABpra		✓	
gsk-3	ABar→ABal	✓		
	ABala→ABara	✓		
	ABpra→ABpla		✓	
	E→MS	✓		
	C→EMS	✓		
lag-1	ABp→ABa	✓		✓supported by NHR-25 expression data, but not by those of PHA-4. PHA-4 expression is lost in AB lineage.
	ABalp→ABarp	✓		
	ABara→ABala		✓	
	ABpla→ABpra		✓	
mex-3	AB→8C		✓	
	D→P4	✓		
mex-5	AB→2EMS	✓		
	P_4→D	✓		
mom-2	ABar→ABal		✓	ABaraa→ABalpa is observed.
	ABpra→ABpla		✓	
	E→MS	✓		
par-2	ABp→ABa		✓	
	ABalp→ABarp		✓	
	P_2→EMS	✓		
	E→MS	✓		
par-6	ABp→ABa		✓	Ectopic PHA-4 expression observed in various AB sub-lineages.
	ABalp→ABarp		✓	
	ABara→ABala		✓	
	P_2→EMS	✓		
	E→MS	✓		
pie-1	ABp→ABa		✓	
	P_2→EMS		✓	
pop-1	ABpla→ABpra		✓	ABara→ABalp is observed.
	MS→E	✓		
rba-1	ABala→ABara	✓		PHA-4 expression is lost in ABalp.
skn-1	ABalp→ABarp	✓		
	ABara→ABala		✓	
	ABpra→ABpla	✓		
	EMS→2C	✓		
*skr-2**	ABp→ABa		✓	E→MS is observed.
	ABala→ABara	✓		
	ABpra→ABpla		✓	
	MS→EMS		✓	
	C→EMS		✓	
	Ca→C		✓	
	P_4→P_3	✓		
src-1	P_4→D	✓		
wwp-1	ABa→ABp		✓	
	ABpla→ABpra		✓	

* The RNAi phenotypes of *glp-1/lin-12* or *skr-2* are contrasting with those of *glp-1* or *skr-1/2* RNAi by Du et al (2014).

Supplementary Table S6. List of sister pairs between which ADS is bigger than 5 minutes in wild-type embryos.

Name of sister pair	Average ADS	Standard deviation (SD)
P4-D	29.0	4.5
MSaaapp-MSaaapa	28.4	5.0
ABalappaa-ABalappap	12.6	3.4
MSaapap-MSaapaa	10.1	2.3
ABarpaaa-ABarpaap	9.7	2.2
MSaapp-MSaapa	9.5	2.0
ABaraaapp-ABaraaapa	9.5	2.5
ABpraaapa-ABpraaapp	9.2	2.3
ABalpaapp-ABalpaapa	9.2	2.2
MSpapp-MSpapa	8.7	1.6
Caap-Caaa	8.6	2.7
Cppa-Cppp	8.4	2.1
ABalapapa-ABalapapp	8.4	2.6
ABalpaaa-ABalpaap	8.3	2.1
MSpapap-MSpapaa	8.3	1.9
ABaraaaa-ABaraaap	8.2	2.2
ABpraaaaa-ABpraaaap	8.1	2.6
ABarapaaa-ABarapaap	8.1	2.9
Capa-Capp	7.8	2.1
MSppapp-MSppapa	7.5	2.4
ABplaaapa-ABplaaapp	7.4	3.2
ABalppaa-ABalppap	7.4	2.0
ABalpppaa-ABalpppap	7.3	2.2
ABplpappp-ABplpappa	7.3	2.2
ABarpappa-ABarpappp	7.2	2.8
ABalaappa-ABalaappp	7.2	2.9
ABpraappa-ABpraappp	7.2	2.6
ABaraaaap-ABaraaaaa	7.1	3.5
ABalappa-ABalappp	6.8	1.8
ABalppppa-ABalppppp	6.7	2.0
ABarppaaa-ABarppaap	6.7	2.9
P3-C	6.7	0.9
MSapapp-MSapapa	6.6	2.4
MSpaap-MSpaaa	6.4	1.5
ABarpppaa-ABarpppap	6.2	2.4
Eplp-Epla	6.1	3.2
ABplaappa-ABplaappp	6.1	2.2
Eprp-Epra	5.8	3.3
ABalaapa-ABalaapp	5.8	1.7
ABarappaa-ABarappap	5.7	2.5
ABalaaaa-ABalaaap	5.5	2.1
Daa-Dap	5.5	1.7
MSaaaap-MSaaaaa	5.4	2.1
Dpa-Dpp	5.4	1.7
ABprapaap-ABprapaaa	5.2	2.6
Cap-Caa	5.1	1.8

Supplementary Table S7. List of genes whose perturbation produces overall slowdown

Gene name	Sequence name	KOG Information (merged)
ama-1	F36A4.7	RNA polymerase II, large subunit
C34B2.8	C34B2.8	NADH:ubiquinone oxidoreductase, B16.6 subunit/cell death-regulatory protein
C50F2.3	C50F2.3	mRNA splicing factor
cacn-1	W03H9.4	Cactin
cdc-25.2	F16B4.8	M-phase inducer phosphatase
cdc-73	F35F11.1	Ortholog of mammalian and Saccharomyces cerevisiae CDC73/Parafibromin that is a member of the PAF1 (Polymerase-Associated Factor 1) complex
cdk-2	K03E5.3	Protein kinase PCTAIRE and related kinases
cdk-9	H25P06.2	Cyclin T-dependent kinase CDK9
ceh-32	W05E10.3	Transcription factor SIX and related HOX domain proteins
cpsf-1	Y76B12C.7	mRNA cleavage and polyadenylation factor II complex, subunit CFT1 (CPSF subunit)
cpsf-2	F09G2.4	mRNA cleavage and polyadenylation factor II complex, subunit CFT2 (CPSF subunit)
cpsf-3	Y67H2A.1	mRNA cleavage and polyadenylation factor II complex, BRR5 (CPSF subunit)
cye-1	C37A2.4	G1/S-specific cyclin E
cyh-1	Y49F6B.1	Cyclin H
cyl-1	C52E6.4	Cyclin L
D1081.8	D1081.8	mRNA splicing protein CDC5 (Myb superfamily)
dap-3	C14A4.2	Mitochondrial ribosome small subunit component, mediator of apoptosis DAP3
dhfr-1	C36B1.7	Dihydrofolate reductase
E04A4.5	E04A4.5	Mitochondrial import inner membrane translocase, subunit TIM17
emb-5	T04A8.14	Transcription elongation factor SPT6
F10B5.8	F10B5.8	Predicted cleavage and polyadenylation specificity factor (CPSF subunit)
F19F10.12	F19F10.12	Predicted cleavage and polyadenylation specificity factor (CPSF subunit)
F33D11.10	F33D11.10	Predicted ATP-dependent RNA helicase FAL1, involved in rRNA maturation, DEAD-box superfamily
F55A3.3	F55A3.3	Global transcriptional regulator, cell division control protein
F58F12.1	F58F12.1	Mitochondrial F1F0-ATP synthase, subunit delta/ATP16
F59C6.5	F59C6.5	NADH-ubiquinone oxidoreductase, subunit NDUFB10/PDSW
gad-1	T05H4.14	Uncharacterized conserved protein, contains WD40 repeat
gsy-1	Y46G5A.31	Glycogen synthase
hmp-2	K05C4.6	Armadillo/beta-Catenin/plakoglobin
hsp-6	C37H5.8	Molecular chaperones mortalin/PBP74/GRP75, HSP70 superfamily
iftb-1	K04G2.1	Translation initiation factor 2, beta subunit (eIF-2beta)
imb-3	C53D5.6	Karyopherin (importin) beta 3
inf-1	F57B9.6	Translation initiation factor 4F, helicase subunit (eIF-4A) and related helicases
mdt-26	C25H3.6	Mediator protein
mex-5	W02A2.7	CCCH-type Zn-finger protein
mrpl-18	D2007.4	Mitochondrial Ribosomal Protein, large subunit
mrpl-9	B0205.11	Mitochondrial Ribosomal Protein, large subunit
nhr-6	C48D5.1	Nuclear receptors of the nerve growth factor-induced protein B type
nud-1	F53A2.4	Nuclear distribution protein NUDC
pfs-2	R06A4.9	Polyadenylation factor I complex, subunit PFS2

phb-1	Y37E3.9	Prohibitin
phf-5	Y54F10BM.14	Uncharacterized conserved protein, contains CXXC motifs
pole-2	F08B4.5	DNA polymerase epsilon, subunit B
pri-1	F58A4.4	Eukaryotic-type DNA primase, catalytic (small) subunit
prp-19	T10F2.4	Yeast PRP (splicing factor) related
prp-31	Y110A7A.8	Yeast PRP (splicing factor) related
prp-38	D1054.14	Yeast PRP (splicing factor) related
rba-1	K07A1.11	Nucleosome remodeling factor, subunit CAF1/NURF55/MSI1
repo-1	F11A10.2	Splicing factor 3a, subunit 2
rnf-113	K01G5.1	Predicted E3 ubiquitin ligase
rnp-7	K04G7.10	U1 small nuclear ribonucleoprotein (RRM superfamily)
rpb-3	C36B1.3	RNA Polymerase II (B) subunit
rpn-5	F10G7.8	26S proteasome regulatory complex, subunit RPN5/PSMD12
rps-10	D1007.6	40s ribosomal protein s10
snr-3	T28D9.10	Small nuclear ribonucleoprotein SMD1 and related snRNPs
stip-1	C07E3.1	Septin and Tuftelin Interacting Protein) homolog, a potential component in a multisubunit complex with the splicing factor
T26A5.8	T26A5.8	DNA polymerase epsilon, subunit D
taf-1	W04A8.7	TBP-associated transcription factor
taf-4	R119.6	TBP-associated transcription factor

KOG: Eukaryotic Orthologous Groups of proteins

Supplementary Table S8. Role of the identified regulatory genes of ADS in cell fate specification

Gene	Phenotypes upon perturbation	Literature	Pathway
mex-1	ABa ---> MS<br>ABp ---> MS<br>P3 adopts muscle fate. (P4 ---> D)	(Mello et al, 1992; Victor et al, 2002)	Early maternal
mex-6	Muscle excess	(Page et al, 2001)	Early maternal
	No intestine<br>Excess hyperdermal cells<br>Excess pharyngeal cells	(Huang et al, 2002)	
pal-1	ABar ---> ABal	(Walston et al, 2004)	Early maternal
par-2	P2 ---> EMS<br>E --->MS	(Bowerman et al, 1997)	Early maternal
par-6	ABp ---> ABa	(Du et al, 2014; Mango et al, 1994; Mello et al, 1994; Watts et al, 1996)	Early maternal
	ABalp ---> ABarp<br>ABara ---> ABala	(Du et al, 2014; Hutter & Schnabel, 1994; Watts et al, 1996)	
	P2 ---> EMS<br>E --->MS	(Bowerman et al, 1997; Du et al, 2014)	
pie-1	ABp ---> ABa	(Mango et al, 1994)	Early maternal
	P2 ---> EMS	(Mello et al, 1992)	
skn-1	ABalp ---> ABarp<br>ABara ---> ABala	(Hutter & Schnabel, 1994); (Shelton & Bowerman, 1996).	Early maternal
	ABpra ---> ABpla	(Du et al, 2014; Hutter & Schnabel, 1994; Hutter & Schnabel, 1995)	
	EMS ---> 2C	(Bowerman et al, 1992)	
cul-1	Abnormal postembryonic cell division of vulval cells	(Fay & Han, 2000)	E3 Ligase
lin-23	Cp ---> E	(Segref et al, 2010)	E3 Ligase
skr-2	ABar ---> ABal<br>ABala ---> ABara	(Du et al, 2014)	E3 Ligase
	ABpra ---> ABpla	(Du et al, 2014; Hutter & Schnabel, 1995)	
	MS ---> EMS	(Du et al, 2014)	
	C ---> EMS	(Du et al, 2014; Lin, 2003; Shirayama et al, 2006)	
	Ca ---> C<br>P4 ---> P3	(Du et al, 2014)	
dsh-2	No intestine	(Bei et al, 2002)	Wnt signaling
gsk-3	ABar ---> ABal	(Du et al, 2014; Walston et al, 2004)	Wnt signaling
	ABala ---> ABara	(Du et al, 2014)	
	ABpra ---> ABpla	(Du et al, 2014; Hutter & Schnabel, 1994; Hutter & Schnabel, 1995)	
	C ---> EMS	(Maduro et al, 2001)	

	E --->MS	(Schlesinger et al, 1999)	
kin-19	E --->MS<br>Excess pharyngeal cells	(Peters et al, 1999)	Wnt signaling
lit-1	E --->MS<br>MSap ---> MSaa<br>MSpp ---> MSpa	(Kaletta et al, 1997)	Wnt signaling
mom-2	ABar ---> ABal	(Du et al, 2014; Hutter & Schnabel, 1994; Rocheleau et al, 1997; Thorpe et al, 1997; Walston et al, 2004)	Wnt signaling
	ABpra ---> ABpla	(Du et al, 2014; Hutter & Schnabel, 1994; Hutter & Schnabel, 1995)	
	E --->MS	(Rocheleau et al, 1997; Thorpe et al, 1997)	
pop-1	ABpla ---> ABpra	(Du et al, 2014; Hutter & Schnabel, 1994; Hutter & Schnabel, 1995; Lin et al, 1998)	Wnt signaling
	MS ---> E	(Lin et al, 1995)	
	ABxxa ---> ABxxp	(Lin et al, 1998)	
wrm-1	No intestine	(Maduro et al, 2001)	Wnt signaling
	No endoderm<br>Excess pharyngeal cells	(Rocheleau et al, 1997)	
	E --->MS	This study	
src-1	ABar ---> ABal	(Walston et al, 2004)	MES-1-SRC-1 signaling
	P4 ---> D	(Bei et al, 2002)	
lag-1	ABp--->ABa<br>ABara--->ABala<br>ABalp--->ABarp	(Du et al, 2014)	Notch signaling
	ABplaa--->ABpra	(Moskowitz & Rothman, 1996)	
lin-12/ glp-1	ABp--->ABa	(Mello et al, 1994)	Notch signaling
	ABara--->ABala<br>ABalp--->ABarp	(Hutter & Schnabel, 1994)	
sel-8	Production of coelomocytes instead of sex myoblasts in ventral 4-M cells of the M-lineage	(Foehr & Liu, 2008)	Notch signaling
	No anterior pharynx<br>No rectum<br>Nose twisted	(Doyle et al, 2000)	
ceh-13	Mislocalization of hypodermal and body-wall muscle cells during embryonic development	(Brunschwig et al, 1999)	Transcription factor
ceh-43	Loss of dopaminergic neuron identity	(Doitsidou et al, 2013)	Transcription factor
	Detachment of pharynx from mouth hypodermis, arrested at 3-fold stage embryo.	(Aspock & Burglin, 2001)	
die-1	Loss of asymmetry in ASE	(Sarin et al, 2007)	Transcription factor
egl-18	Failure in proper seam cells differentiation	(Koh & Rothman, 2001)	Transcription factor

ham-1	Production of extra HSN-like cells	(Desai et al, 1988)	Transcription factor
	Transformation of sister cell HSN/PHB precursor into a second HSN/PHB precursor	(Guenther & Garriga, 1996)	
	Production of extra neurons<br>Large cell-death corpses	(Frank et al, 2005)	
	Defective Q.a asymmetric division	(Feng et al, 2013)	
hlh-2	Cell fate transformation in germline DTC cell	(Chesney et al, 2009)	Transcription factor
nhr-25	Affects the fate of seam-cell anterior daughters in L2 worms	(Silhankova et al, 2005)	Transcription factor
	Production of extra gonad arms	(Chesney et al, 2009)	
	Vulvaless	(Hwang & Sternberg, 2004)	
nob-1	Transformation of posterior into anterior in gut and nervous system<br>Eprp ---> Earp	(Van Auken et al, 2000)	Transcription factor
	Disruption of asymmetric divisions of T cells, leading to the production of hypodermal cells instead of neural cells<br>Division failed in T cells	(Arata et al, 2006)	
plp-1	No endoderm	(Witze et al, 2009)	Transcription factor
sptf-3	Affect the orientation of P7.p lineage	(Ulm et al, 2011)	Transcription factor
	Fate transformation of pharyngeal I3 interneuron to pharyngeal gland cell 1P(sister cell)<br>Defective M4 sister, AQR sisters, g1A sisters and I2 sisters cell death	(Hirose & Horvitz, 2013)	
tbx-33	Neuron ---> excretory cell	This study	Transcription factor
tbx-37/ tbx-38	ABalpppaaaa, ABalppaapa, ABarppaaap divide but does not differentiate or undergo apoptosis<br>ABalpppapp undergoes apoptosis	(Good et al, 2004)	Transcription factor
cbp-1	Lack of mesodermal, endodermal, or hypodermal differentiation	(Shi & Mello, 1998; Victor et al, 2002)	Chromatin modification
	Production of extra neuronal cells		
cbp-2	Lack of mesodermal, endodermal, or hypodermal differentiation	This study	Chromatin modification
epc-1	Multivulva	(Ceol & Horvitz, 2004)	Chromatin modification
let-526	Disruption of asymmetric tlp-1 and psa-3 expression in the T cell lineage<br>Loss of gonad arms	(Shibata et al, 2012)	Chromatin modification
lex-1	Negatively regulates induction of VPC	(Tseng et al, 2007)	Chromatin modification
lin-40	Disruptions of vulval induvtion and transverse division during vulva morphogenesis	(Chen & Han, 2001)	Chromatin modification
snfc-5	Increased apoptosis	(Checchi & Engebrecht, 2011; Green et al, 2011)	Chromatin modification
	Protruding vulva	(Cui et al, 2004)	
swsn-3	Protruding vulva	(Cui et al, 2004; Simmer et al, 2003)	Chromatin modification

Supplementary Table S9. Manually curated list of cell cycle related genes

RNAi targeted gene	Early embryonic arrest*	KOG description
air-2	Yes#	Serine/threonine protein kinase
cdc-14		Protein tyrosine phosphatase CDC14
cdc-25.2	Yes#	M-phase inducer phosphatase
cdc-37		Cell division cycle 37 protein, CDC37
cdc-42		Ras-related small GTPase, Rho type
cdc-6		Pre-initiation complex, subunit CDC6, AAA+ superfamily ATPase
cdk-1	Yes#	Cyclin-dependent kinase
cdk-2		Cyclin-dependent kinase
cdk-4		Cyclin-dependent kinase
cdk-5		Cyclin-dependent kinase CDK5
cdk-8		Cyclin dependent kinase CDK8
cdk-9		Cyclin T-dependent kinase CDK9
cdka-1		CDK5 kinase activator p35/Nck5a
cdk-12		Cdc2-related protein kinase
chk-1	Yes#	Checkpoint kinase and related serine/threonine protein kinases
cki-1		Cyclin-dependent kinase inhibitor
cyb-1		Cyclin B and related kinase-activating proteins
cyb-3	Yes#	Cyclin B and related kinase-activating proteins
cyd-1		G1/S-specific cyclin D
cye-1	Yes#	G1/S-specific cyclin E
cyh-1	Yes#	Cdk activating kinase (CAK)/RNA polymerase II transcription initiation/nucleotide excision repair factor TFIIH/TFIIK, cyclin H subunit
cyl-1	Yes#	Cyclin L
dpl-1	Yes^	Transcription factor E2F/dimerization partner (TDP) DP-Like
F55A3.3	Yes#	Global transcriptional regulator, cell division control protein
gld-2		S-M checkpoint control protein CID1 and related nucleotidyltransferases
lin-35		Rb (Retinoblastoma tumor suppressor)-related protein
M03F8.3	Yes#	Cell cycle control protein (crooked neck)
mdf-2		Spindle assembly checkpoint protein
plk-1	Yes#	Polo-like serine/threonine protein kinase
rad-51		DNA repair protein RAD51/RHP55
wee-1.3	Yes#	Cyclin-dependent kinase WEE1 homolog

KOG: Eukaryotic Orthologous Group; gene listed in Figure 5 is highlighted in blue;# gene depletion produced early embryonic arrest and embryo became not editable; *arrest before 300 cells; ^ no embryo produced

Supplementary Table S10. Actual p values and ADS in minutes that are used in Fig 5 (attached as a separate Excel file)

Supplementary Figures

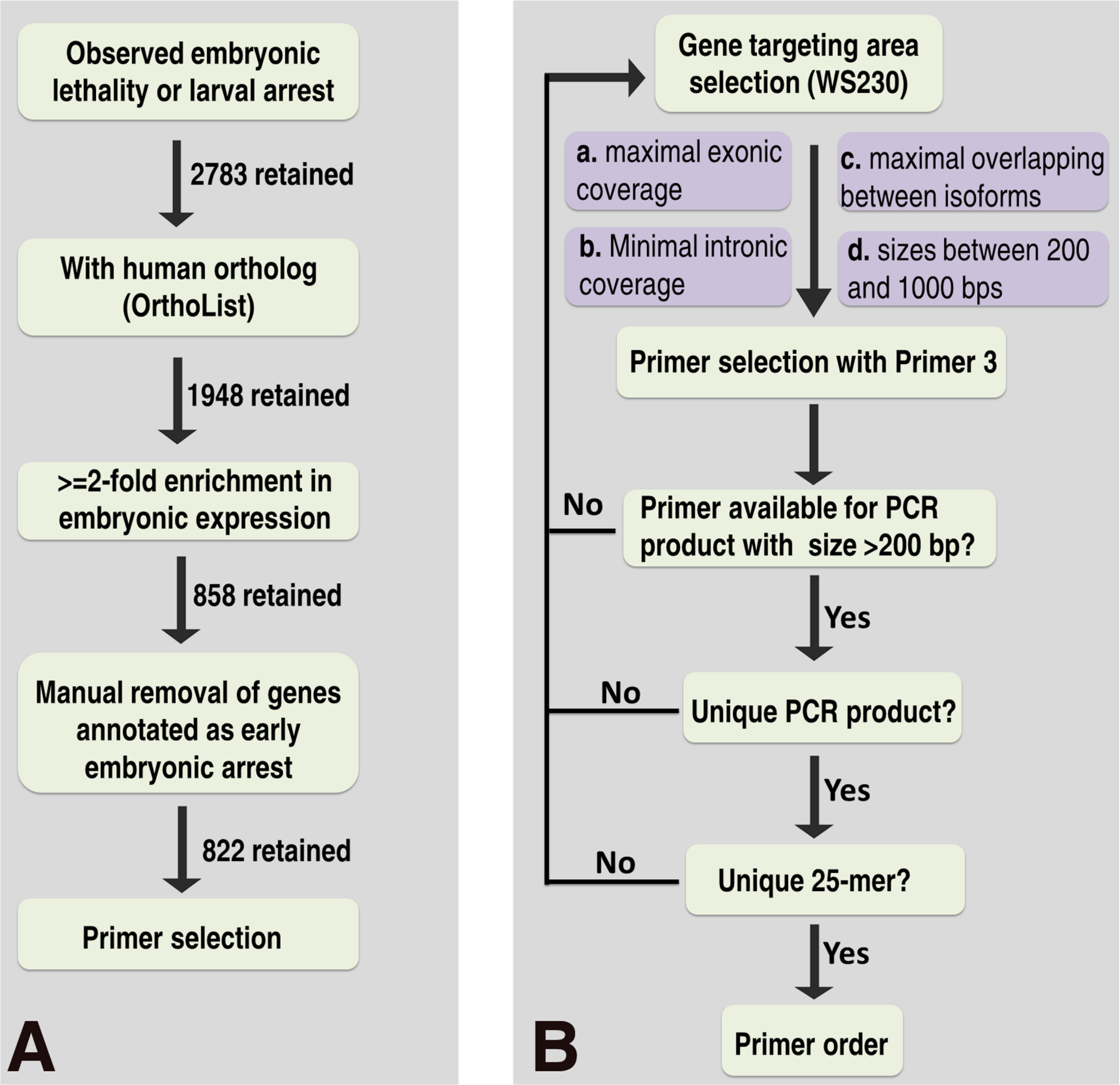

Supplementary Fig S1 Gene prioritization and primer selection. (A) Flow chart of gene prioritization. Genes associated with embryonic lethality and/or early larval arrest were retrieved from Wormbase (WS230). The gene list was further filtered with a requirement of at least two-fold enrichment in embryonic expression compared to that of the mixed stage. Genes reported to produce early embryonic arrest upon perturbation were manually removed. Only those that contain an unambiguous human ortholog were retained. (B) Flow chart of primer selection for the synthesis of dsRNA.

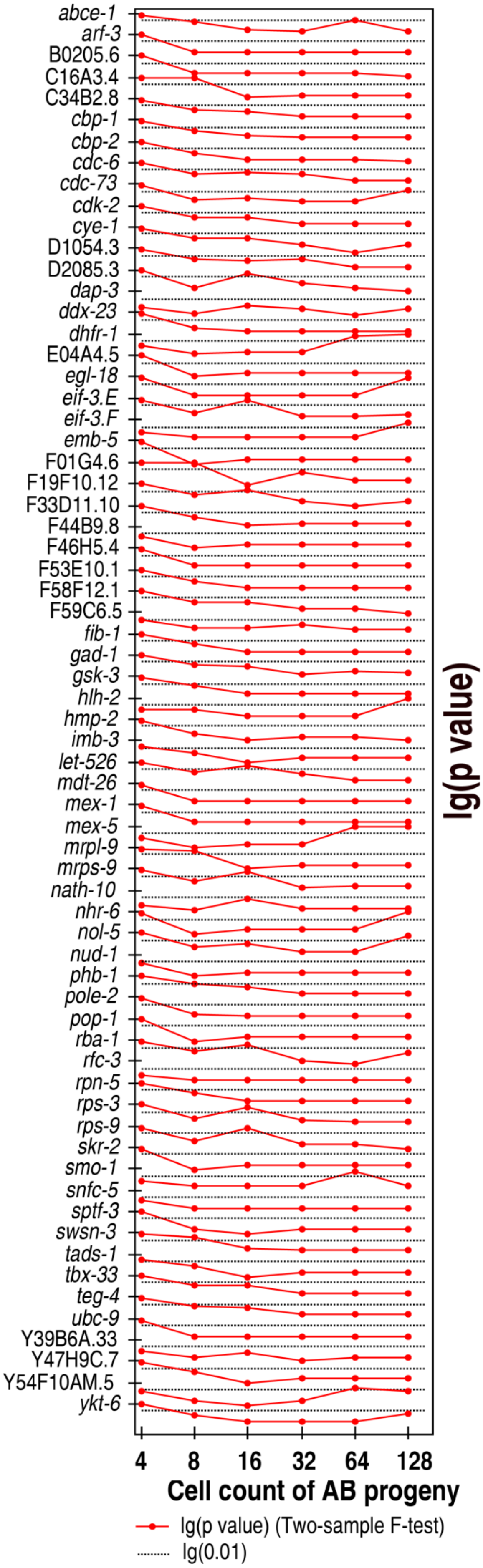

Supplementary Fig S2 List of genes with highly variable division timing upon perturbation. Only data from AB4 to AB128 are used for the calculation. Shown are the genes with a significantly higher variation (dispersion) in division timing for at least four out of six consecutive generations (p<0.01) in AB progeny (see Materials and Methods). Vertical and horizontal axes denote the lg(p value) and the round of AB division (indicated by the number of AB progeny) respectively. Dash lines indicated the value of lg(0.01). Names of the perturbed genes are indicated on the left.

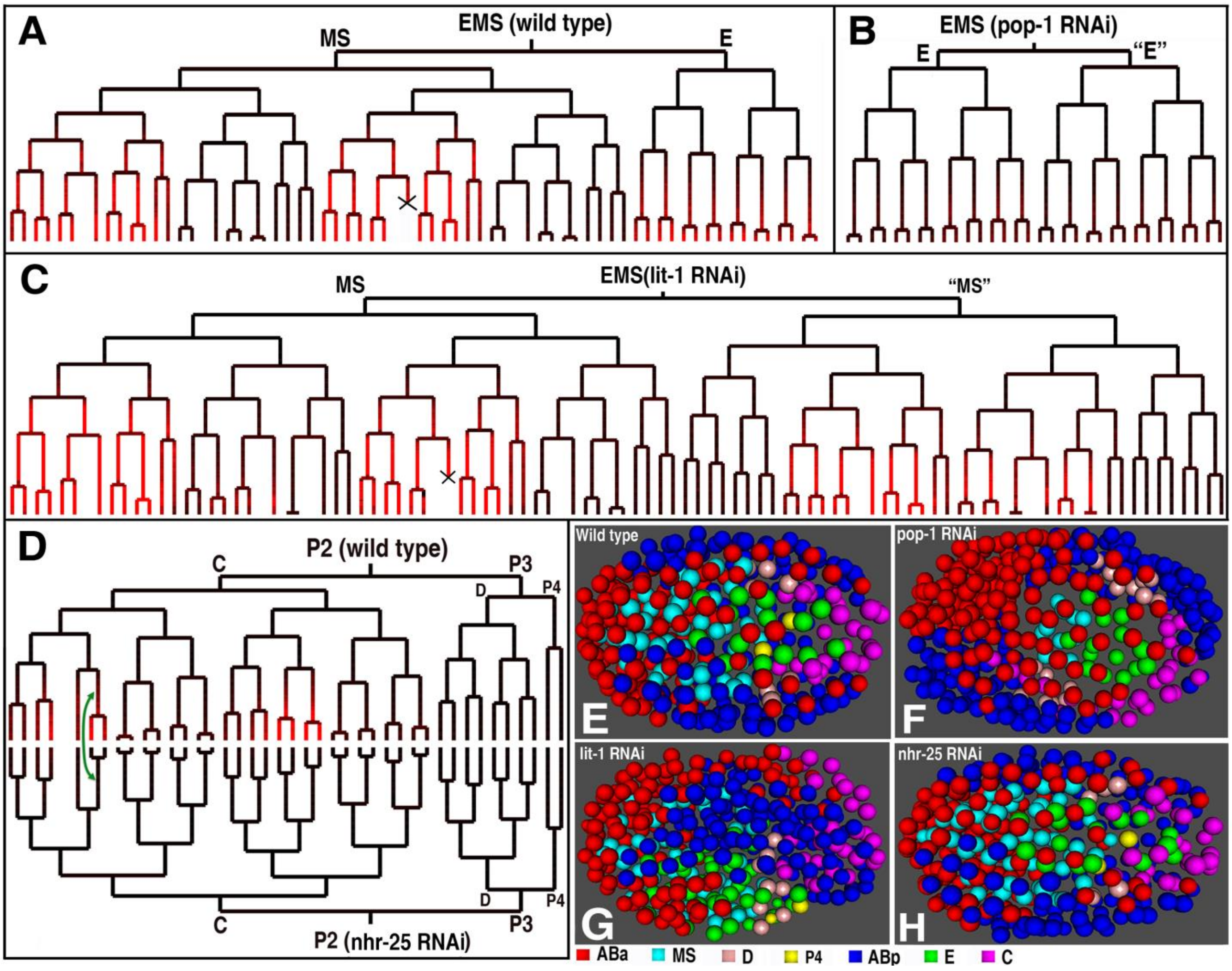

Supplementary Fig S3 Validation of the experimental pipeline (see also Fig 3). (A-C) EMS lineage trees with superimposed PHA-4 expression (colored in red) for embryos of wild-type (A), RNAi against *pop-1* (B) and *lit-1* (C). Note the expected “MS” to “E” like cell fate transformation by *pop-1* RNAi and the opposite fate transformation by *lit-1* RNAi. Cell death is indicated by an “X”. (D) Pairwise comparison of P2 lineage with superimposed NHR-25 expression (colored in red) between the wild-type (top) and *nhr-25* RNAi (bottom) embryos. An elongated division timing of Caapp between wild-type and the RNAi embryos was highlighted with a double-headed arrow. (E-H) Space-filling models for the nuclei of approximately 350-celled embryos of a wild-type (E), RNAi against *pop-1* (F), *lit-1* (G) and *nhr-2*5 (H) respectively. The nuclei are differentially color-coded as indicated. Note the symmetric migration of ABa and ABp progeny was obvious in the wild-type embryo but was severely disrupted in the embryos treated with RNAi against *pop-1* and *lit-1*.

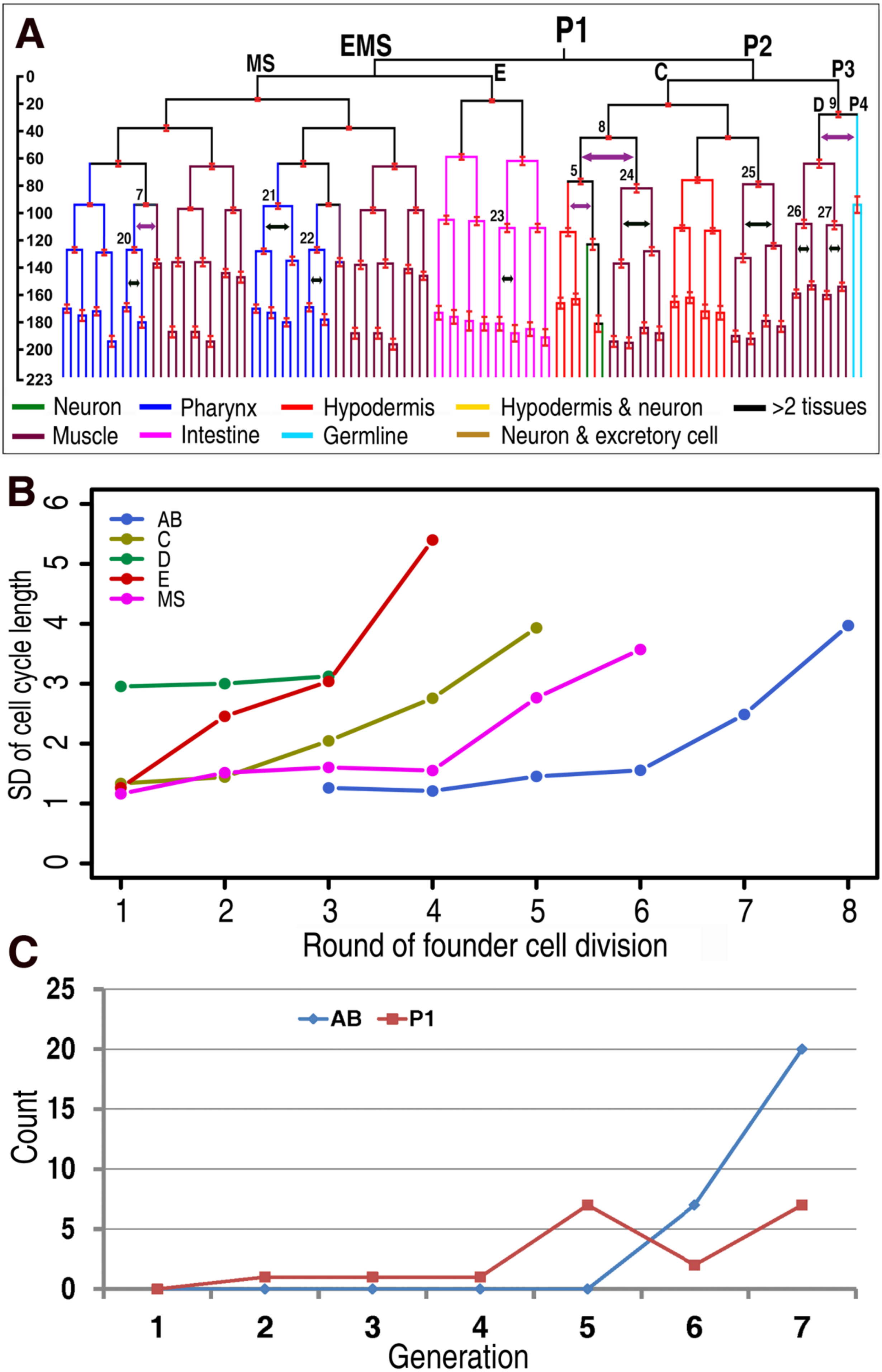
A
P1
EMS
MS
E
P2
C
P3
D
9
P4
8
5
24
25
7
21
20
22
23
26
27
0
20
40
60
80
100
120
140
160
180
200
223
Neuron
Pharynx
Hypodermis
Hypodermis & neuron
>2 tissues
Muscle
Intestine
Germline
Neuron & excretory cell
B
AB
C
D
E
MS
SD of cell cycle length
0
1
2
3
4
5
6
1
2
3
4
5
6
7
8
Round of founder cell division
C
AB
P1
Count
0
5
10
15
20
25
1
2
3
4
5
6
7
Generation

Supplementary Fig S4 Reproducibility of cell division timing. (A) A P1 cell lineage tree derived from the average division timings of 91 wild-type embryos of approximately 350-cells with standard deviations (SD) indicated in red bars on division nodes. Developing time in minutes shown on the left starts from last time point of EMS to the cut-off time point. Cell fates of P1 descendants and the sister pairs used in screening of ADS are differentially color-coded in the same way as that in Fig 4 A and B. (B) Change in standard deviation (SD) (vertical axis) of the division timing in minutes over first 8 rounds of cell division (horizontal axis) in 91 wild-type embryos. SDs derived from the descendants of AB, C, D, E and MS are plotted and differentially color-coded. (C) Count of the cells (vertical axis) whose ADS is longer than five minutes over the round of cell division (horizontal axis). Only the daughters of AB and P1 are shown.

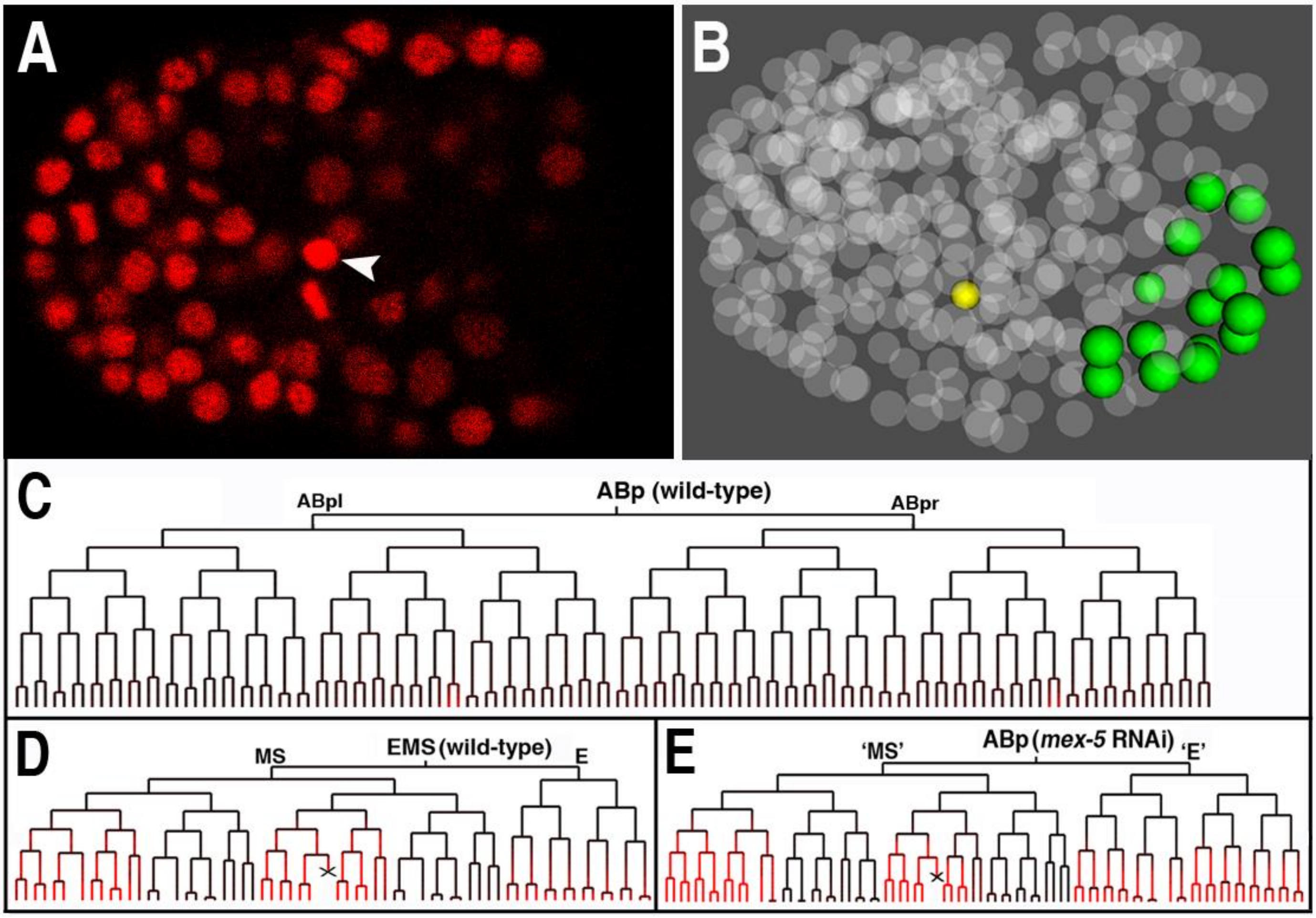

Supplementary Fig S5 An example of the decrease in cell count due to the homeotic cell fate transformation upon gene perturbation. Shown is the fate transformation from "ABp" to "EMS" like by RNAi of against *mex-5*. (A) Fluorescence micrograph of a *mex-5* RNAi embryo, showing that an "ABp" descendant, "ABplpaapp", is transformed into a "MSpaapp" like fate as revealed by automated lineaging and apoptosis phenotype. Note the cell is undergoing apoptosis as judged by its aggregated fluorescent signal (indicated with an arrow head). (B) A space-filling model of the nuclei from the same embryo as that in (A). The apoptotic cell is colored in yellow, the intestine cells in green and the remaining ones were rendered transparent. (C and E) "ABp" lineage trees from a wild-type and a *mex-5* RNAi (E) embryos. (D) "EMS" lineage tree from a wild-type embryo. Lineal expression of PHA-4::GFP is colored in red and cell death is indicated by an "X".

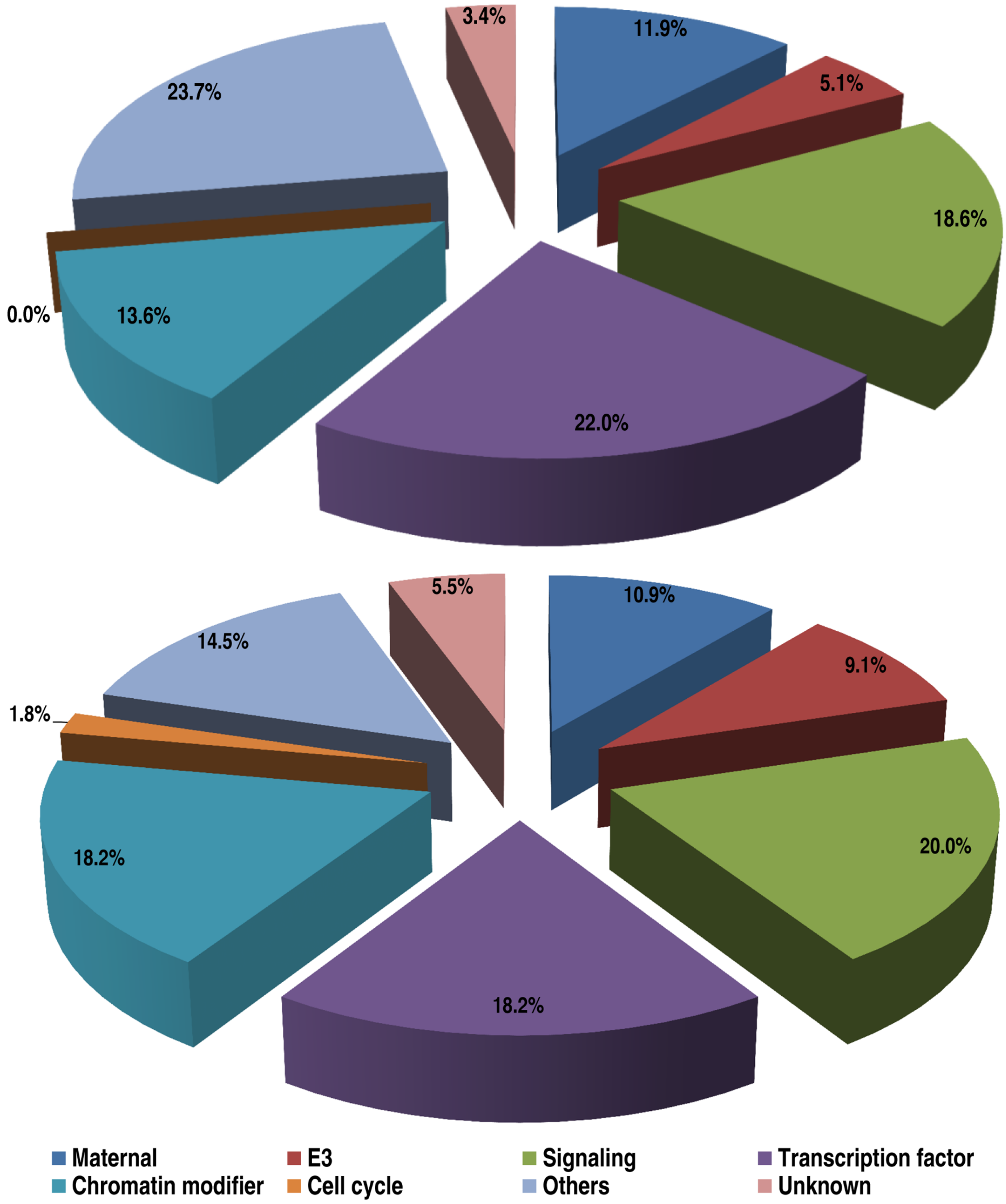

Supplementary Fig S6 Distribution of the genes involved in temporal coordination across pathways/functional groups. Temporal genes operating during cell fate specification or tissue growth are shown on the top and bottom panels respectively.

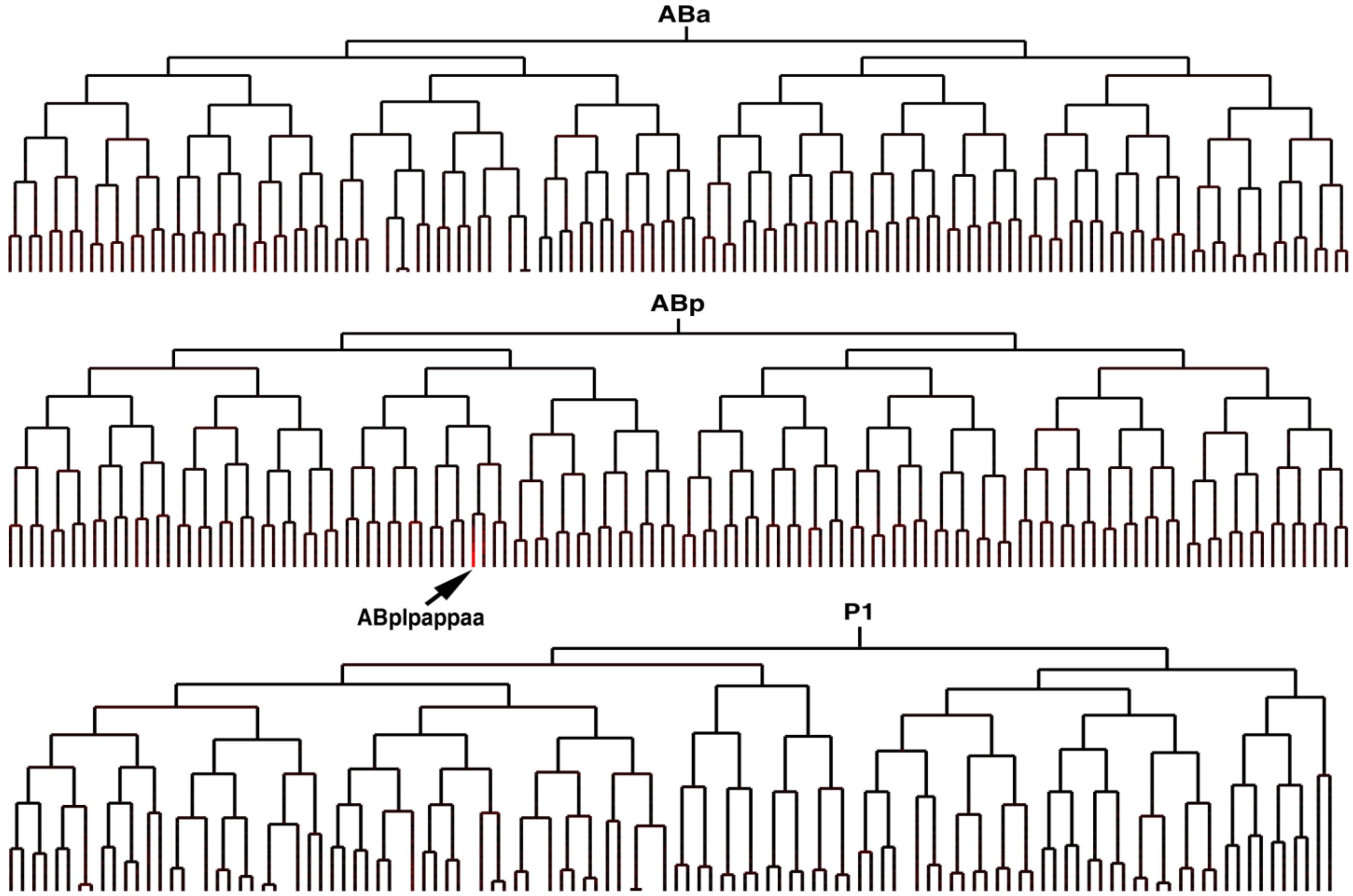

Supplementary Fig S7 Lineal expression of CEH-26. Shown are the trees with lineal expression of CEH-26 at approximately 350-cell stage of *C. elegans* embryo. The expression of CEH-26::GFP (colored in red) in the cell “ABplpappaa” is indicated with an arrow (see also Fig. 6A). The posterior daughter of the cell develops into the excretory cell.

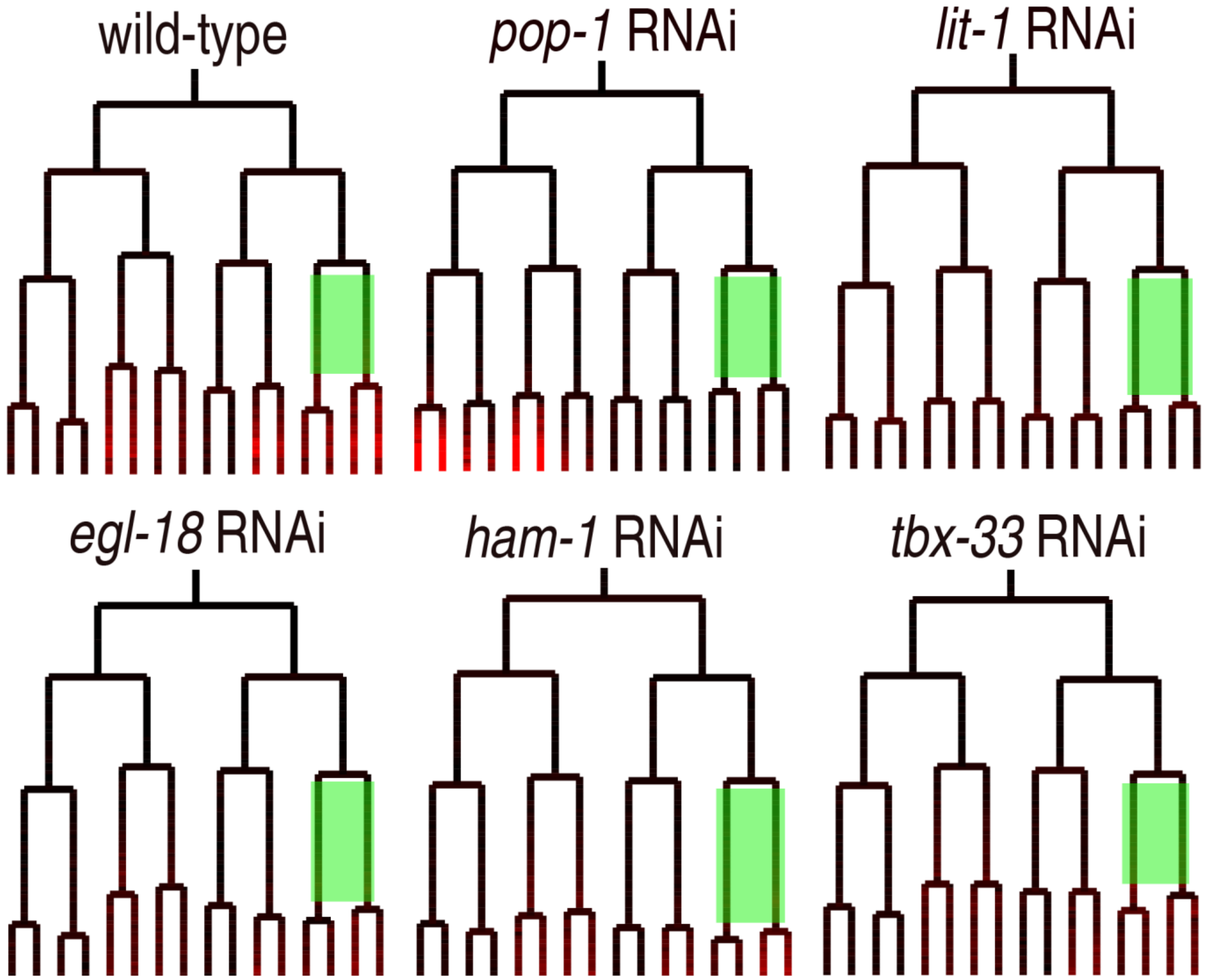

Supplementary Fig S8 Lineal expression of NHR-25 (colored in red) in wild-type and RNAi embryos with genotypes indicated on the top. All trees are rooted with "ABarpa". The two sister cells used for the calculation of ADS are shaded in green.

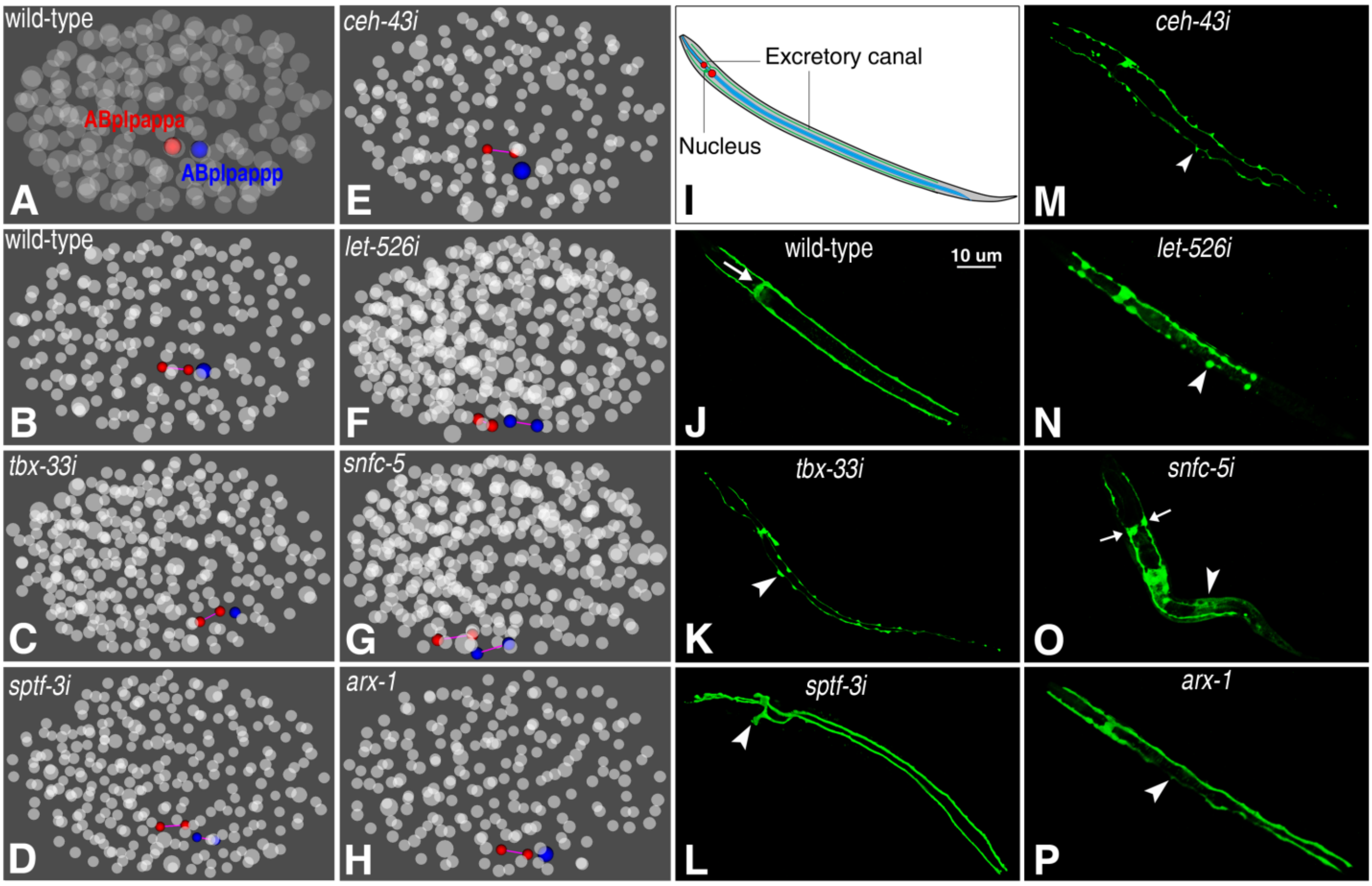

Supplementary Fig S9 Embryonic and postembryonic defects in cell migration after RNAi against the genes involved in regulating the ADS of the excretory cell precursor "ABplpapp". (A) Embryonic positions of "ABplpappa" (red dot) and "ABplpappp" (blue dot) at the last time point of "ABplpappa" in a wild-type embryo. (B) The division orientation of "ABplpappa" in a wild-type embryo. (C-H) Embryonic positions and division orientations of "ABplpappa" and "ABplpappp" at the first time point of the cell "ABplpappaa" in the RNAi embryos against *tbx-33* (C), *sptf-3* (D), *ceh-43* (E), *let-526* (F), *snfc-5* (G) and *arx-1* (H) respectively. (I) A cartoon showing the morphology of *C. elegans* excretory cell at L1 stage. (J) Fluorescence micrograph of a wild-type L1-stage worm expressing a excretory cell-specific marker pgp-12::GFP. The cell body of excretory cell consisting of a single nucleus is indicated with an arrow. (K-P) Fluorescence micrographs of L1-stage animals expressing pgp-12::GFP from embryos treated with the RNAi against *tbx-33* (K), *sptf-3* (L), *ceh-43* (M), *let-526* (N), *snfc-5* (O) and *arx-1* (P) respectively. Examples of the defects in the excretory canal are indicated with an arrowhead. Two nuclei of excretory cell (O) are indicated with an arrow.

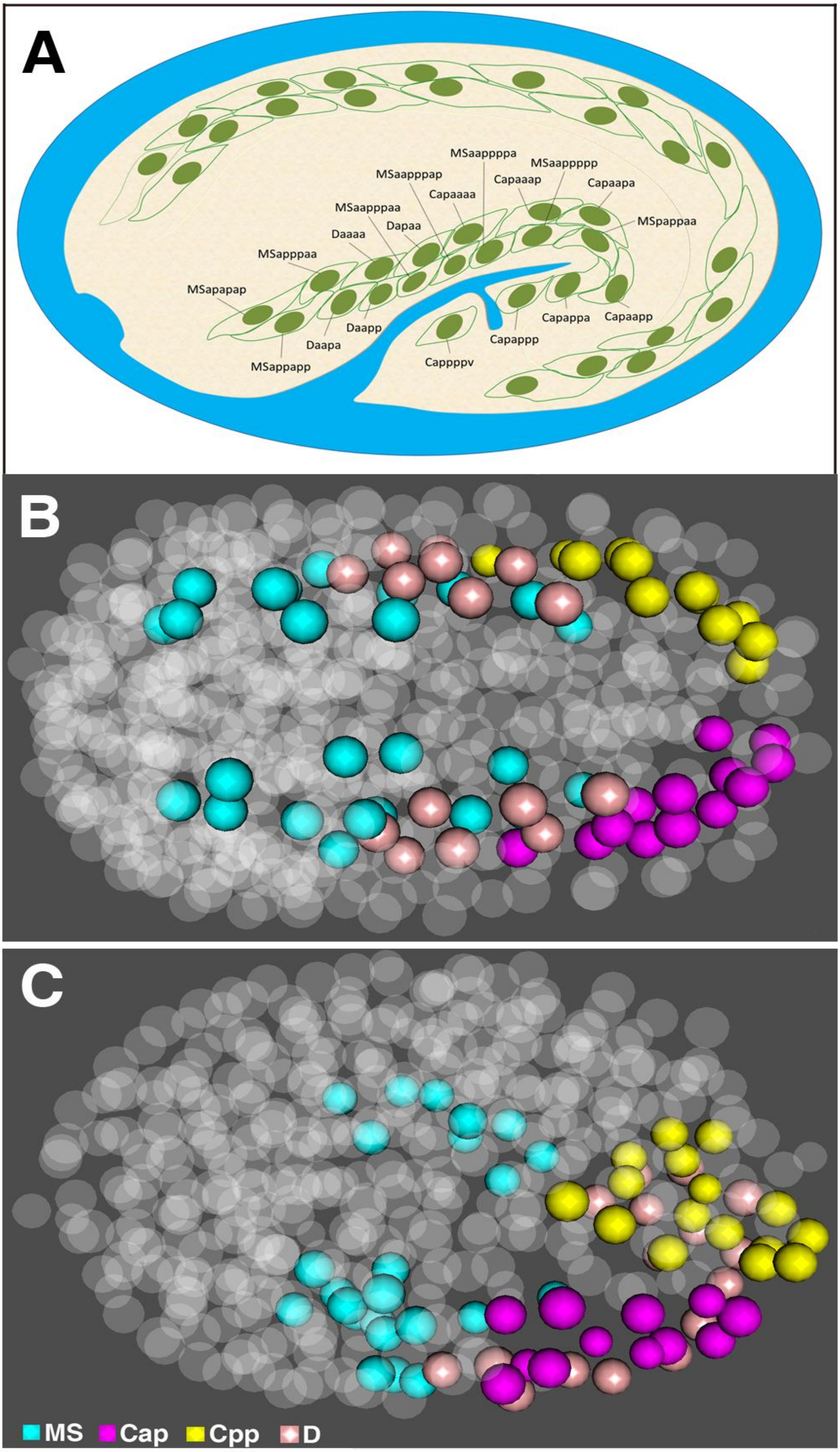
A
MSaappppa
MSaappppp
MSaapppap
Capaaap
Capaapa
Capaaaa
MSaapppaa
MSpappaa
Dapaa
Daaaa
MSapppaa
MSapapap
Capappa
Capaapp
Daapp
Capappp
Daapa
MSappapp
Cappppv
B
C
MS
Cap
Cpp
D

Supplementary Fig S10 Cell migrations during the formation of body-wall muscles. (A) A diagram showing the side view of an embryo at one-and-a-half-fold stage, which highlights the arrangement of body-wall muscle cells originated from MS, C and D lineages (modified from Wormatlas). (B and C) A 3D space-filling model showing the nuclei from 550-cell stage embryos of the wild-type and *tads-1* RNAi respectively. Body-wall muscle cells derived from “MS”, “C” and “D” lineages are differentially color-coded while the remaining cells are rendered transparent.

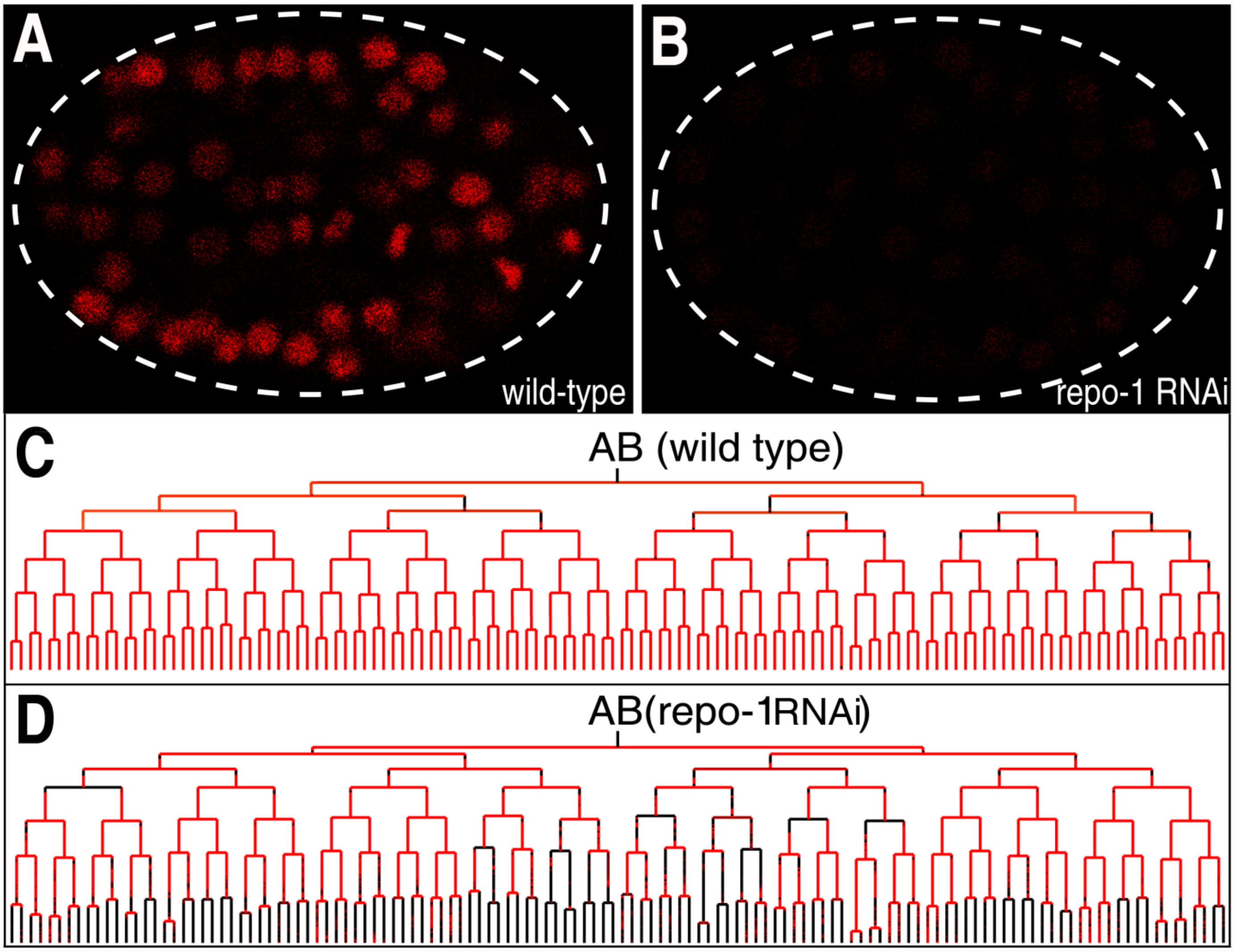

Supplementary Fig S11 Inactivation of REPO-1 inhibits the zygotic expression of lineaging marker, HIS-72::mCherry. A and B, fluorescence micrographs showing the expression of HIS-72::mCherry in a wild-type and REPO-1 depleted embryos respectively. C and D, the lineal expression of HIS-72::mCherry in a wild-type and REPO-1 depleted embryo respectively.

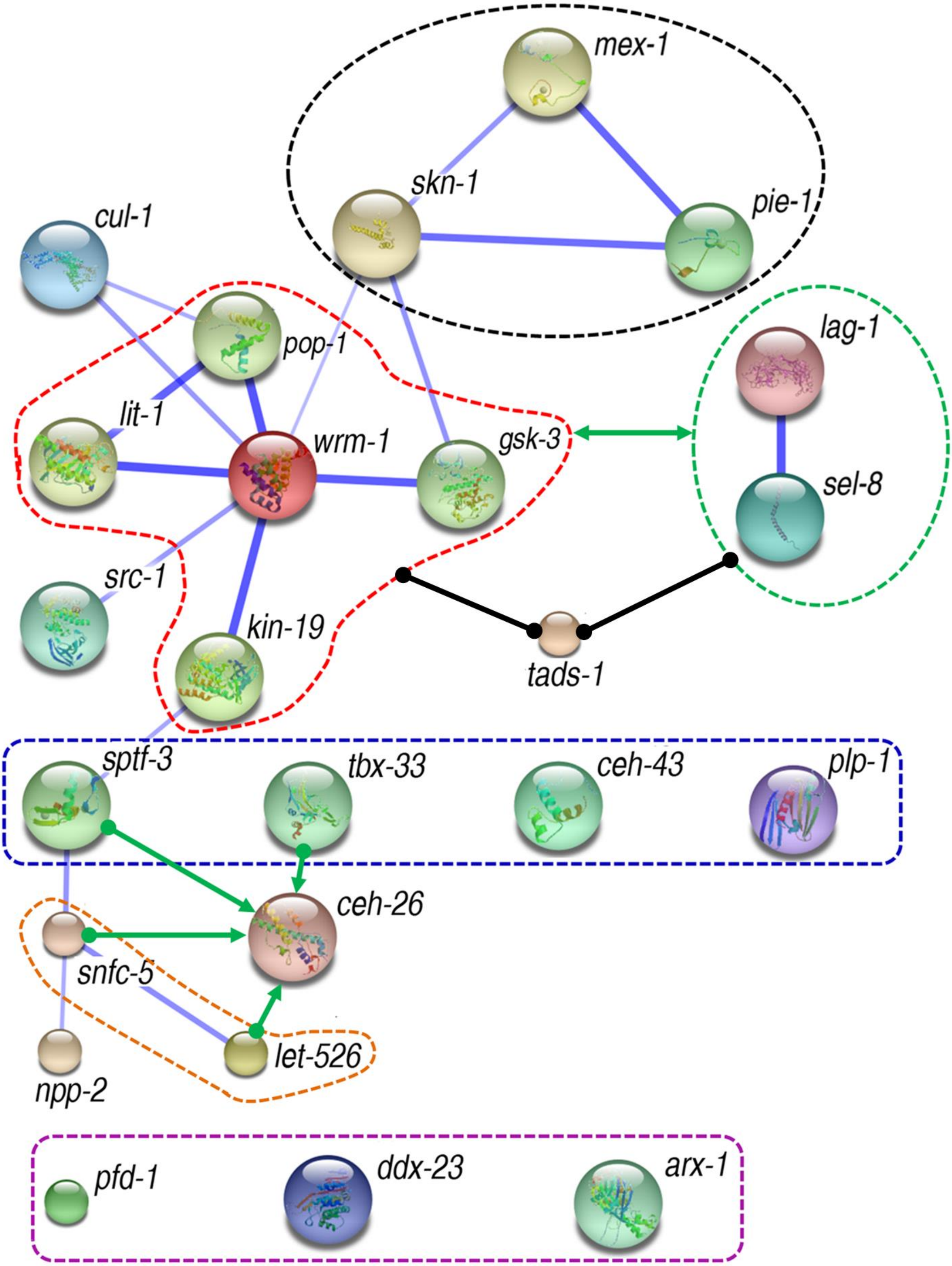
mex-1
skn-1
pie-1
cul-1
pop-1
lag-1
lit-1
wrm-1
gsk-3
sel-8
src-1
kin-19
tads-1
sptf-3
tbx-33
ceh-43
plp-1
ceh-26
snfc-5
let-526
npp-2
pfd-1
ddx-23
arx-1

Supplementary Fig S12 A putative gene network of excretory cell development inferred using STRING with the genes involved in ADS control of the excretory cell precursor, “ABplpapp”. Shown is the confidence view using all the genes involved in regulating the ADS of “ABplpapp” as an input. Genes are arbitrarily grouped into modules based on their existing functional information with color-coded dash lines (module name indicated). Green arrows indicate gene activation based on our lineal expression analysis while the double-headed arrow denotes a potential crosstalk. *tads-1* appears to be one of the major target proteins of both Wnt and Notch signaling pathways as inferred from motif analysis (data not shown). The thickness of the blue lines generated with STRING indicates the confidence level of the predicted relationship based on the existing functional data.

Supplementary references

Arata Y, Kouike H, Zhang Y, Herman MA, Okano H, Sawa H (2006) Wnt signaling and a Hox protein cooperatively regulate psa-3/Meis to determine daughter cell fate after asymmetric cell division in *C. elegans*. *Developmental cell* **11:** 105-115

Aspock G, Burglin TR (2001) The Caenorhabditis elegans distal-less ortholog ceh-43 is required for development of the anterior hypodermis. *Developmental dynamics : an official publication of the American Association of Anatomists* **222:** 403-409

Bei Y, Hogan J, Berkowitz LA, Soto M, Rocheleau CE, Pang KM, Collins J, Mello CC (2002) SRC-1 and Wnt signaling act together to specify endoderm and to control cleavage orientation in early *C. elegans* embryos. *Developmental cell* **3:** 113-125

Bowerman B, Eaton BA, Priess JR (1992) skn-1, a maternally expressed gene required to specify the fate of ventral blastomeres in the early *C. elegans* embryo. *Cell* **68:** 1061-1075

Bowerman B, Ingram MK, Hunter CP (1997) The maternal par genes and the segregation of cell fate specification activities in early Caenorhabditis elegans embryos. *Development* **124:** 3815-3826

Brunschwig K, Wittmann C, Schnabel R, Burglin TR, Tobler H, Muller F (1999) Anterior organization of the Caenorhabditis elegans embryo by the labial-like Hox gene ceh-13. *Development* **126:** 1537-1546

Ceol CJ, Horvitz HR (2004) A new class of *C. elegans* synMuv genes implicates a Tip60/NuA4-like HAT complex as a negative regulator of Ras signaling. *Developmental cell* **6:** 563-576

Checchi PM, Engebrecht J (2011) Caenorhabditis elegans histone methyltransferase MET-2 shields the male X chromosome from checkpoint machinery and mediates meiotic sex chromosome inactivation. *PLoS genetics* **7:** e1002267

Chen Z, Han M (2001) Role of *C. elegans* lin-40 MTA in vulval fate specification and morphogenesis. *Development* **128:** 4911-4921

Chesney MA, Lam N, Morgan DE, Phillips BT, Kimble J (2009) *C. elegans* HLH-2/E/Daughterless controls key regulatory cells during gonadogenesis. *Dev Biol* **331:** 14-25

Cui M, Fay DS, Han M (2004) lin-35/Rb cooperates with the SWI/SNF complex to control Caenorhabditis elegans larval development. *Genetics* **167:** 1177-1185

Desai C, Garriga G, McIntire SL, Horvitz HR (1988) A genetic pathway for the development of the Caenorhabditis elegans HSN motor neurons. *Nature* **336:** 638-646

Doitsidou M, Flames N, Topalidou I, Abe N, Felton T, Remesal L, Popovitchenko T, Mann R, Chalfie M, Hobert O (2013) A combinatorial regulatory signature controls terminal differentiation of the dopaminergic nervous system in *C. elegans*. *Genes & development* **27:** 1391-1405

Doyle TG, Wen C, Greenwald I (2000) SEL-8, a nuclear protein required for LIN-12 and GLP-1 signaling in Caenorhabditis elegans. *Proceedings of the National Academy of Sciences of the United States of America* **97:** 7877-7881

Du Z, Santella A, He F, Tiongson M, Bao Z (2014) De novo inference of systems-level mechanistic models of development from live-imaging-based phenotype analysis. *Cell* **156:** 359-372

Fay DS, Han M (2000) Mutations in cye-1, a Caenorhabditis elegans cyclin E homolog, reveal coordination between cell-cycle control and vulval development. *Development* **127:** 4049-4060

Feng G, Yi P, Yang Y, Chai Y, Tian D, Zhu Z, Liu J, Zhou F, Cheng Z, Wang X, Li W, Ou G (2013) Developmental stage-dependent transcriptional regulatory pathways control neuroblast lineage progression. *Development* **140:** 3838-3847

Foehr ML, Liu J (2008) Dorsoventral patterning of the *C. elegans* postembryonic mesoderm requires both LIN-12/Notch and TGFbeta signaling. *Dev Biol* **313:** 256-266

Frank CA, Hawkins NC, Guenther C, Horvitz HR, Garriga G (2005) *C. elegans* HAM-1 positions the cleavage plane and regulates apoptosis in asymmetric neuroblast divisions. *Dev Biol* **284:** 301-310

Good K, Ciosk R, Nance J, Neves A, Hill RJ, Priess JR (2004) The T-box transcription factors TBX-37 and TBX-38 link GLP-1/Notch signaling to mesoderm induction in *C. elegans* embryos. *Development* **131:** 1967-1978

Green RA, Kao HL, Audhya A, Arur S, Mayers JR, Fridolfsson HN, Schulman M, Schloissnig S, Niessen S, Laband K, Wang S, Starr DA, Hyman AA, Schedl T, Desai A, Piano F, Gunsalus KC, Oegema K (2011) A high-resolution *C. elegans* essential gene network based on phenotypic profiling of a complex tissue. *Cell* **145:** 470-482

Guenther C, Garriga G (1996) Asymmetric distribution of the *C. elegans* HAM-1 protein in neuroblasts enables daughter cells to adopt distinct fates. *Development* **122:** 3509-3518

Hirose T, Horvitz HR (2013) An Sp1 transcription factor coordinates caspase-dependent and -independent apoptotic pathways. *Nature* **500:** 354-358

Huang NN, Mootz DE, Walhout AJ, Vidal M, Hunter CP (2002) MEX-3 interacting proteins link cell polarity to asymmetric gene expression in Caenorhabditis elegans. *Development* **129:** 747-759

Hutter H, Schnabel R (1994) glp-1 and inductions establishing embryonic axes in *C. elegans*. *Development* **120:** 2051-2064

Hutter H, Schnabel R (1995) Establishment of left-right asymmetry in the Caenorhabditis elegans embryo: a multistep process involving a series of inductive events. *Development* **121:** 3417-3424

Hwang BJ, Sternberg PW (2004) A cell-specific enhancer that specifies lin-3 expression in the *C. elegans* anchor cell for vulval development. *Development* **131:** 143-151

Kaletta T, Schnabel H, Schnabel R (1997) Binary specification of the embryonic lineage in Caenorhabditis elegans. *Nature* **390:** 294-298

Koh K, Rothman JH (2001) ELT-5 and ELT-6 are required continuously to regulate epidermal seam cell differentiation and cell fusion in *C. elegans*. *Development* **128:** 2867-2880

Lin R (2003) A gain-of-function mutation in oma-1, a *C. elegans* gene required for oocyte maturation, results in delayed degradation of maternal proteins and embryonic lethality. *Dev Biol* **258:** 226-239

Lin R, Hill RJ, Priess JR (1998) POP-1 and anterior-posterior fate decisions in *C. elegans* embryos. *Cell* **92:** 229-239

Lin R, Thompson S, Priess JR (1995) pop-1 encodes an HMG box protein required for the specification of a mesoderm precursor in early *C. elegans* embryos. *Cell* **83:** 599-609

Maduro MF, Meneghini MD, Bowerman B, Broitman-Maduro G, Rothman JH (2001) Restriction of mesendoderm to a single blastomere by the combined action of SKN-1 and a GSK-3beta homolog is mediated by MED-1 and -2 in *C. elegans*. *Molecular cell* **7:** 475-485

Mango SE, Thorpe CJ, Martin PR, Chamberlain SH, Bowerman B (1994) Two maternal genes, apx-1 and pie-1, are required to distinguish the fates of equivalent blastomeres in the early Caenorhabditis elegans embryo. *Development* **120:** 2305-2315

Mello CC, Draper BW, Krause M, Weintraub H, Priess JR (1992) The pie-1 and mex-1 genes and maternal control of blastomere identity in early *C. elegans* embryos. *Cell* **70:** 163-176

Mello CC, Draper BW, Priess JR (1994) The maternal genes apx-1 and glp-1 and establishment of dorsal-ventral polarity in the early *C. elegans* embryo. *Cell* **77:** 95-106

Moskowitz IP, Rothman JH (1996) lin-12 and glp-1 are required zygotically for early embryonic cellular interactions and are regulated by maternal GLP-1 signaling in Caenorhabditis elegans. *Development* **122:** 4105-4117

Page BD, Guedes S, Waring D, Priess JR (2001) The *C. elegans* E2F- and DP-related proteins are required for embryonic asymmetry and negatively regulate Ras/MAPK signaling. *Molecular cell* **7:** 451-460

Peters JM, McKay RM, McKay JP, Graff JM (1999) Casein kinase I transduces Wnt signals. *Nature* **401:** 345-350

Rocheleau CE, Downs WD, Lin R, Wittmann C, Bei Y, Cha YH, Ali M, Priess JR, Mello CC (1997) Wnt signaling and an APC-related gene specify endoderm in early *C. elegans* embryos. *Cell* **90:** 707-716

Sarin S, O'Meara MM, Flowers EB, Antonio C, Poole RJ, Didiano D, Johnston RJ, Jr., Chang S, Narula S, Hobert O (2007) Genetic screens for Caenorhabditis elegans mutants defective in left/right asymmetric neuronal fate specification. *Genetics* **176:** 2109-2130

Schlesinger A, Shelton CA, Maloof JN, Meneghini M, Bowerman B (1999) Wnt pathway components orient a mitotic spindle in the early Caenorhabditis elegans embryo without requiring gene transcription in the responding cell. *Genes & development* **13:** 2028-2038

Segref A, Cabello J, Clucas C, Schnabel R, Johnstone IL (2010) Fate specification and tissue-specific cell cycle control of the Caenorhabditis elegans intestine. *Molecular biology of the cell* **21:** 725-738

Shelton CA, Bowerman B (1996) Time-dependent responses to glp-1-mediated inductions in early *C. elegans* embryos. *Development* **122:** 2043-2050

Shi Y, Mello C (1998) A CBP/p300 homolog specifies multiple differentiation pathways in Caenorhabditis elegans. *Genes & development* **12:** 943-955

Shibata Y, Uchida M, Takeshita H, Nishiwaki K, Sawa H (2012) Multiple functions of PBRM-1/Polybromo- and LET-526/Osa-containing chromatin remodeling complexes in *C. elegans* development. *Dev Biol* **361:** 349-357

Shirayama M, Soto MC, Ishidate T, Kim S, Nakamura K, Bei Y, van den Heuvel S, Mello CC (2006) The Conserved Kinases CDK-1, GSK-3, KIN-19, and MBK-2 Promote OMA-1 Destruction to Regulate the Oocyte-to-Embryo Transition in *C. elegans*. *Current biology : CB* **16:** 47-55

Silhankova M, Jindra M, Asahina M (2005) Nuclear receptor NHR-25 is required for cell-shape dynamics during epidermal differentiation in Caenorhabditis elegans. *Journal of cell science* **118:** 223-232

Simmer F, Moorman C, van der Linden AM, Kuijk E, van den Berghe PV, Kamath RS, Fraser AG, Ahringer J, Plasterk RH (2003) Genome-wide RNAi of *C. elegans* using the hypersensitive rrf-3 strain reveals novel gene functions. *PLoS biology* **1:** E12

Thorpe CJ, Schlesinger A, Carter JC, Bowerman B (1997) Wnt signaling polarizes an early *C. elegans* blastomere to distinguish endoderm from mesoderm. *Cell* **90:** 695-705

Tseng RJ, Armstrong KR, Wang X, Chamberlin HM (2007) The bromodomain protein LEX-1 acts with TAM-1 to modulate gene expression in *C. elegans*. *Molecular genetics and genomics : MGG* **278:** 507-518

Ulm EA, Sleiman SF, Chamberlin HM (2011) Developmental functions for the Caenorhabditis elegans Sp protein SPTF-3. *Mechanisms of development* **128:** 428-441

Van Auken K, Weaver DC, Edgar LG, Wood WB (2000) Caenorhabditis elegans embryonic axial patterning requires two recently discovered posterior-group Hox genes. *Proceedings of the National Academy of Sciences of the United States of America* **97:** 4499-4503

Victor M, Bei Y, Gay F, Calvo D, Mello C, Shi Y (2002) HAT activity is essential for CBP-1-dependent transcription and differentiation in Caenorhabditis elegans. *EMBO reports* **3:** 50-55

Walston T, Tuskey C, Edgar L, Hawkins N, Ellis G, Bowerman B, Wood W, Hardin J (2004) Multiple Wnt signaling pathways converge to orient the mitotic spindle in early *C. elegans* embryos. *Developmental cell* **7:** 831-841

Watts JL, Etemad-Moghadam B, Guo S, Boyd L, Draper BW, Mello CC, Priess JR, Kemphues KJ (1996) par-6, a gene involved in the establishment of asymmetry in early *C. elegans* embryos, mediates the asymmetric localization of PAR-3. *Development* **122:** 3133-3140

Witze ES, Field ED, Hunt DF, Rothman JH (2009) *C. elegans* pur alpha, an activator of end-1, synergizes with the Wnt pathway to specify endoderm. *Dev Biol* **327:** 12-23
