# Supplementary material for: Systems-level quantification of division timing reveals a common genetic architecture controlling asynchrony and fate asymmetry
Source: Mol Syst Biol. 2015 Jun 15;11(6):814. doi: 10.15252/msb.20145857 (PMC4501849; doi:10.15252/msb.20145857)
Supplement: Supplementary file 8 [file msb0011-0814-sd8.pdf]

Systems-level quantification of division timing reveals a common genetic architecture controlling asynchrony and fate asymmetry

Vincy Wing Sze Ho, Ming-Kin Wong, Xiaomeng An, Daogang Guan, Jiaofang Shao, Hon Chun Kaoru Ng, Xiaoliang Ren, Kan He, Jinyue Liao, Yingjin Ang, Long Chen, Xiaotai Huang, Bin Yan, Yiji Xia, Leanne LH Chan, King L Chow, Hong Yan and Zhongying Zhao

Corresponding author: Zhongying Zhao, Hong Kong Baptist University

Review timeline:

Submission date:	21 October 2014
Editorial Decision:	03 December 2014
Revision received:	24 February 2015
Editorial Decision:	17 April 2015
Revision received:	27 April 2015
Editorial Decision:	13 May 2015
Revision received:	18 May 2015
Accepted:	19 May 2015

Editor: Maria Polychronidou

Transaction Report:

(Note: With the exception of the correction of typographical or spelling errors that could be a source of ambiguity, letters and reports are not edited. The original formatting of letters and referee reports may not be reflected in this compilation.)

1st Editorial Decision 03 December 2014

Thank you again for submitting your work to Molecular Systems Biology. We have now heard back from the three referees who agreed to evaluate your manuscript. As you will see below, the reviewers acknowledge that the presented data could represent a useful resource. However, they raise a series of concerns and make suggestions for modifications, which we would ask you to address in a revision of this work.

Since the comments and recommendations of the referees are very clear, there is no need to repeat them. However, some of the more fundamental issues are the following:
- An important point raised by reviewer #1 refers to the need to perform further analyses in order to better support some of the conclusions and increase the level of insight resulting from the acquired data.
- As all three referees point out, the text needs to be extensively edited in order to improve the presentation of the data and the overall clarity of the manuscript. Moreover, the figures should be edited to include all necessary information and they should be properly referenced in the text.
- Several clarifications regarding data analysis need to be provided (i.e. filtering criteria for selecting genes for further analysis, exact number of analyzed genes, statistical significance, hypothetical additional possible outcomes of gene depletion that are currently not discussed etc.).
- The data need to be carefully presented and discussed in order to avoid overstatements. Additional references should be included in order to properly place the work in the context of the existing

literature.
- Along the lines of comment #2 of reviewer #1, we would recommend avoiding the introduction of novel acronyms whenever possible, in order to make the main message of the paper easily accessible to a broad audience.
The referees provide constructive and detailed suggestions regarding the points listed above, and all other issues that are mentioned in their reports.

Reviewer #1:

This manuscript describes analysis of embryogenesis in C. elegans up to the 350-cell stage using time-lapse microscopy at 1.5-minute intervals and semi-automated cell lineaging using previously established software. The authors measure cell division timings of WT and embryos treated with RNAi against 822 genes and compare differences in both cell division timing and cell lineages, with the aim of elucidating functional relationships between cell cycle timing and cell fate decisions that influence cell migration and patterning.
Overall, the authors do a reasonably thorough job of making the measurements that form the foundation of the study, and they have assembled a very nice dataset.
There are a number of concerns regarding the presentation, analysis, and interpretation of results:

1) The manuscript contains a huge number of grammatical and typographical errors. These make it more difficult to follow the narrative and require correction before the manuscript could be considered for publication. Some sentences are simply nonsensical. For example, on p. 8: "As described earlier, the anterior and posterior daughters of the two cells became terminally differentially and stem cells respectively."

2) I have an issue with the nomenclature the authors have chosen to describe the phenomenon they are primarily interested in, i.e. "TADS (temporal asymmetry of division between sister cells)". The standard nomenclature for this kind of phenotype is "asynchrony", not "temporal asymmetry" (which is a term that does not make sense to me). For example, in the C. elegans embryo, there is a well-known asymmetry [a spatial descriptor] in the first mitotic cell division (which gives rise to a larger anterior AB cell and a smaller posterior P0 cell), as well as asynchrony [a temporal descriptor] of cell divisions of the two daughter cells (AB divides earlier than P0), and differences in orientation of division planes due to differential rotation of the spindle (P0 rotates and divides along the AP axis, whereas AB does not and divides transversely). The authors would do well to observe more commonly accepted standards of vocabulary to describe these phenotypes, rather than come up with new and unnecessary nomenclature. As I understand it, the phenotype being addressed here is a difference in the length of the cell cycle between daughter cells (time from birth to division). Why not refer to this simply as a measure of asynchrony, which is the same thing and can be described/defined in whatever units are useful (here, minutes)? Similarly, cell division timings that are identical can simply be referred to as "synchronous". In addition, "DDT" is a well-known pesticide and it would be preferable to avoid using this acronym altogether.

3) In order to validate their pipeline, the authors examined cell lineage and tissue marker expression for genes that are known to produce embryonic defects when depleted. RNAi of cbp-1 results in extra cell divisions when compared to WT (Figure 3A). The authors also show that there is a net difference in division timing between the cbp-1 depleted embryos and WT. While this seems significant, its unclear if this net difference in cell division timing has a direct bearing on cell fate. Are there any conditional alleles/hypomorphic cbp-1 alleles available to test if differences in timing of cell divisions have a direct consequence on cell fate?

4) The authors assert on p. 7 that a larger deviation in division timings later in embryogenesis suggests that cell cycle control might be relaxed relative to the early divisions.
a) Is there any other evidence in the literature to suggest this? If so then the authors can include a reference.
b) This argument is not convincing without further investigation/elaboration. It appears from the manuscript that this statement refers to measures of absolute time of cell division with respect to a

much earlier registration point. Thus, one would expect the total deviation over several cell divisions to reflect the cumulative deviation over several generations. If this is the case, then the statement cannot be supported without statistics to show that the deviation is larger than expected, given the historical trajectory.
If, on the other hand, the authors wish to refer to the deviation with respect to the length of the immediately preceding cell division, then they should change the presentation to reflect this (i.e. provide a comparison of variation in the length of earlier cell cycles vs. later cell cycles).
Either of the suggested approaches would essentially address the same question, and I suspect that the observation will not hold up.

5) The authors explain several of their filtering criteria in terms of "further increasing selectivity" for the study. I don't understand the rationale for this. For example, the authors state (p. 7) that they only analyzed data for genes with at least a 50% reduction in DDT. Why not also look for increases in DDT? Or was this not observed?

6) Similarly, on p. 7, why were genes that did not show relatively "normal" speeds included in the analysis? Would these not be expected to produce interesting or relevant results? Is it just because they did not reach the 350-cell stage at the desired time?

7) On p. 9, it is stated that many genes giving rise to TADS of cells with the same fate are the same as those giving rise to TADS of cells with different fates. Do examples of two different kinds of TADS arise within the same lineages? I.e. does a mutation in Wnt signaling produce the two kinds of TADS in different generations of the same lineage, so that cells showing same-fate TADS later differentiate into different fates? This is important considering the sequence of events that occurs along the path of differentiation: chromatin remodeling and changes in gene expression often occur across several cell cycles as cells become progressively committed to a specific differentiated cell fate.

8) On p. 9, there is a long discussion that is essentially a laundry list of results and some vague comments about what they could mean. This discussion is very unsatisfactory. Also the role of SWI/SNF in T cells is noted; what species is this in? What kind of cells are these "T" cells?

9) On p. 11, the authors declare that morphogenesis defects later in development are causally related to earlier defects in cell division timing. This might be true, but could also be very indirect; this appears to be a conflation of correlation with causality between early, proximal phenotypes and much later, secondary / indirect phenotypes. For example, mutation in any of the PAR genes, which are important for cellular asymmetries and correct segregation of cell fate determinants in the one-cell stage, all give rise to "a disorganized mass of partially differentiated cells" with a variable terminal phenotype of several hundred cells (see various publications by Kemphues and Priess). Certainly there is some relationship between these, but many other things went wrong between the initial defects in the one-cell embryo and the terminal stage.

10) Figure 1: B) Why do the authors show only 47 terminal cells, when they measure out to 350 cells? C) The colors of the adjacent lineages do not reflect actual examples from B, which is confusing.

11) Figure 3: B) What does the X-axis represent? Time? This is not described. It also looks as if the cell cycle length in the cbp-1 RNAi condition is not only greater in magnitude on average, but that it shows much higher variability. It has previously been noted that many perturbations release phenotypic variation (e.g. Levy and Siegal). How does this factor into the authors' analysis and conclusions? Does RNAi of other genes result in similar observations?

12) Figure 4: A) The legend and the colors and placement of the double-sided arrows do not match. There appears to be more than one error here, in both the text and the figure. C) In at least one case, the difference in cell cycle length appears to be as long as 30 minutes, but I cannot see this in the dendrogram in (A). This is presumably because that particular difference occurs later, but it's so

anomalous that it is interesting (though not discussed).

13) Figure 5: Parts (A) and (B) describe a higher level of resolution in terms than parts (C) and (D). I don't quite understand the rationale for lumping transcription factors, signaling factors, and chromatin modifiers together as "cell fate" genes. This is not necessarily the case, and doesn't strictly make sense to me. Why not just keep those three categories? Sure, some genes in each of the three classes can affect cell fate decisions, but that is certainly not the case across the board for all genes in these three classes. So, I find this somewhat misleading.

14) Figure 6: (J) The meaning of the red and green dots is not provided. (K) This is just a representation of the data in the STRING database for a subset of genes that the authors recovered in their screen. The connections are based on multiple types of functional connections that are not described at all here; conversely, functional associations that could be derived from the current study (such as quantitative measures of phenotypic similarity) are not represented. The meaning of the blue lines is not indicated, nor are the line weights; moreover the circles and green arrows are just ad hoc cartoons added by the authors. Referring to this as an excretory cell regulatory network, and to these groupings as "modules", seems a bit of a stretch.

15) The authors examined a subset of gene depletions (tbx-33, sptf-3, ceh-43, let526, snfc-5 and arx-1) that resulted in "broken" TADS in the excretory lumen. Further, expression of CEH-26, a secretory cell-specific marker, was eliminated in all cases except ceh-43 and arx-1 (Fig. 6 A-G), thus suggesting a coupled regulation of division timing and cell fate specification. Is the lack of CEH-26 expression a direct consequence of broken TADS? Could the authors test another tissue-specific marker?

16) The authors note that they did not have reproducible results between replicates for a number of genes. Why not perform more than two experiments per gene in order to determine if there was simply an issue with a particular experiment? It's not that hard to repeat RNAi and time-lapse on more replicates. This would make it easier to determine if results could be repeatedly observed, even if RNAi was incomplete in some trials (and therefore elicited variable phenotypes).

17) The use of "ad hoc" on p. 13 is incorrect. I think the authors were looking for something like "de novo", but they should just say that they developed a new database.

18) Figure 7: (A-H) The description of the red and blue dots is incorrect in the legend. The orientation of divisions is more or less A-P in all cases shown; thus the argument that the orientation of division is seriously perturbed here is not at all convincing (in contrast the cells are clearly in the wrong positions in some panels). Why not make multiple measurements for all cases and do a more rigorous statistical evaluation, if the authors wish to assert that these changes are meaningful? (J-M) The legend is inadequate, and arrows appear only in panel (O). In addition, the distinction between the "normal" cell bodies and the extra ones should be more explicit.

19) The connection between spindle orientation and cell migration seems weak. These are different issues and a strong relationship between these is not clearly demonstrated. If the point relates to coordination between the asymmetric segregation of developmental determinants and orientation of the cleavage plane, then this point should be made more transparently. Again, I believe that the authors conflate correlation with causality, which raises some concern; for example, changes in either cell fate or timing can obviously change the environment in which cells find themselves, and thus influence cell migrations. But this is not an earth-shattering idea. What is the new message here?

20) The final two sentences of the discussion should not be the concluding sentences of the entire paper; they belong more in the results or methods somewhere.

© European Molecular Biology Organization

21) Overall, the message of the paper (summarized in Figure 8) is not clear. Differences in cell cycle timing can occur between daughters of identical or different cell fates; in the former case, this can affect total cell proliferation and tissue size, and in the latter may (or may not) relate to the asymmetric segregation of developmental determinants. Mainly, the conclusions of the manuscript are largely phenomenological. The authors have done a large amount of work and collected a nice dataset, but they need to spend more effort to mine the resulting data and come up with some deeper insights into the results.

Reviewer #2:

This manuscript describes an RNAi screen using state-of-the-art lineage tracing for phenotyping. The authors selected 822 genes based on a somewhat arbitrary but defensible set of criteria (reported to be essential, presence of human ortholog(s), mRNA expressed in embryos, lack of known "early embryonic arrest"). After filtering out genes with global effects leading to early arrest or global changes in rate, they focus on about 550 genes and find that RNAi depletion of about 80 of these causes changes in division timing asymmetry between one or more pairs of sister cells. The use of microinjection for RNAi is critical as previous work indicates this is likely to give stronger phenotypes than the more commonly used feeding strategy. Many of these hits are genes also known to be important for fate specification indicating that regulation of cell cycle asymmetry and fate are functionally linked. The novelty of some of these results is overstated in the current text (see below), but once the text is clarified and the data are made more accessible (see below) this will surely be a highly valuable resource for the community.

The main concerns I have are in the presentation and availability of the data. The quality of English is mediocre and the paper could benefit greatly from a detailed edit. I tried to access the website listed by the authors in the discussion to access the raw data, but it appeared that all access to the data required a login. This should be fixed. I have a fairly large number of mostly minor questions and suggestions that should be addressed, listed below.

The "early embryonic arrest" criteria was a bit unclear - what stage "arrest" was required for genes to be excluded using this criterion (or listed as "early embryonic arrest" in Supplemental Table 1)?

Some comparison with the recent work from the Bao lab (e.g. Du et al 2014) is needed. What genes if any were analyzed in common and how did the results compare?

p5 "We applied the pipeline to a total of 822 genes" - Only 762 genes have more than zero embryos and 750 have 2 or more according to Supplemental Table 1. If I understand the methods section this means that only 750 genes were actually analyzed. Figure 2, in contrast lists 865 genes. This should be clarified.

Some note about the consistency of observed phenotypes across replicates would be appropriate in the results section.

Supplemental table 1 - should note which genes were analyzed using reporters other than pha-4

Figure 4, Supplemental table 4 - how do the division timings and standard deviations here compare with those previously reported by Bao et al 2008, Richards et al 2013?

p17. How many embryos were excluded due to failure to reach 300 or 350 cells? What is the effect of removing such embryos - would many of these have had defects in TADS?

A very noticeable writing problem is the repeated use of a very strange sentence construction e.g. pg 7 "we only included genes in our analysis, the depletion of which produced at least a 50% reduction in DDT compared with the average DDT of wild-type embryos between at least one pair of sister cells." This should be rewritten to avoid the ", the depletion of which...". For example: "we only included genes in our analysis whose depletion produced at least a 50% reduction in DDT compared with the average DDT of wild-type embryos between at least one pair of sister cells." This also occurs on pp8,9,10,12,17,26(legend title for Figure 5)

In general the authors overuse the passive voice. For example "An online search database called Phenics is built" would be better as "We built an online search database called Phenics." This occurs throughout the manuscript.

Is the lack of core CC components in TADS regulation (p13) due to the fact that many of them lead to early embryonic lethal phenotypes so they were not considered?

There are previous studies showing CC is influenced by fate determinants (eg Boeck et al, Developmental Biology (2011); Kaletta et al 1997, Lin and Priess (1995)). This result is thus not novel or "surprising" (as described in the abstract). The present study is useful not for identifying a novel role for fate regulators in controlling cell cycle but instead for pairing specific regulators with cells.

p3 "How cell division timings are regulated in vivo to ensure proper cell fate specification or tissue growth... is largely unknown." There is quite a bit of uncited literature on this topic in C. elegans including Bao et al 2008 (for which Dr. Zhao was a coauthor) and various others (Edgar and McGhee, Cell (1988); Nair et al, Development (2013); Budirahardja and Gonczy, Development (2008); Hebeisen and Roy, Development (2008))

Abstract (p2): "cell division timings" - should be "timing" . Similarly "paces" should be "pace."

Abstract - "Unprecedented spatiotemporal resolution" seems inaccurate given the earlier work from the Schnabel, Waterston, Bao and Murray labs. What is unique here is the scope in terms of the number of genes analyzed.

p7 "embryonic stem cells" do not exist in C. elegans in the sense most readers would think of here. I'm not sure what cells the authors are referring to - perhaps Blast cells? "Stem cells" are also referred to in Figure 1C - not clear how the authors define such cells.

A supplemental table listing the magnitude of differences (like Figure 5A/B but with the actual division timing numbers for WT and RNAi embryos and full p value) would be very useful.

Figure 1A: Label for excretory cell (which points to the end of one of the canals) is misleading since the EC cell body is in the head, at the opposite end of the worm.

Figure 2 A-X lettering is unnecessarily complicated. Would be better to label column 1 "A, A', A'..." Column 2 "B, B', B'..." etc.

The idea of using sister asymmetry as a phenotype is an interesting one, but not novel, as this quantity was analyzed in depth for WT embryos in Bao et al (2008). The authors should clarify what is gained by focusing exclusively on "TADS" as opposed to looking more broadly for CC defects. Figure 3B makes it look as if cbp-1 RNAi causes broad changes in division timing, many of which wouldn't effect TADS.

The definition of fate-symmetric vs. fate-asymmetric divisions and color coding in Figures 4 and S3 was confusing. The legend states that cells that give rise to the "same fate" are purple, and different fates are black but it appears this is reversed based on the color scheme. And some tissues (such as pharynx and neuron) are extremely heterogeneous. For example MSpaap gives two "pharyngeal" daughters, yet one produces valve, neuron, marginal cells and cell deaths, while the other produces neurons and muscles. It isn't clear to me that treating these as two separate classes is meaningful. Are these classes partitioned among the genes (is a gene with a TADS defect in one "same fate" division more likely to have additional defects in other "same fate" divisions than in "different fate" divisions?)

Figure 4B - are the axes 91 embryos (label axes). It appears there are a few major groups of embryonic timing profiles from this plot - what are the major differences between the top-left and bottom-right embryos? 4C (legend) should read "DDTs that are bigger than five minutes"

p8 - "suggesting that "differentially putting cell division on hold" may primarily rely on transcription factors rather than chromatin modifiers" conversational - rephrase

© European Molecular Biology Organization

p9 "dyp-28" should be "dpy-28?"

p10 "broke TADS of one tissue" - the word "broke" is very informal. Maybe "disrupted" would be better?

p11 given the focus on the excretory cell maybe Figure S3 "ABpxp" should be part of the main body figure?

p10 - CEH-26. Images in Kolotuev et al 2013 suggest CEH-26/PROS-1 is expressed in many more cells later in embryogenesis. This should be noted (better to say "CEH-26... is specifically expressed in the excretory cell prior to the 350-cell stage?")

The "long-range migration" of the excretory cell stated in the text and implied by Figure 6H/I is somewhat misleading as that cell's "migration" is due more to collective cell movements related to gastrulation - it doesn't really migrate relative to its neighbors. To me this suggests excretory cell migration defects are likely due to larger defects in cell positions within the embryo (such as a failure of gastrulation). In addition it not obvious that 6I represents a significant migration defect compared with 6H.

Figure 6J - can you label the points with significant deviations?

Figure 6K is intriguing. How was this network constructed? "Green arrows indicate gene expression based on lineal expression analysis" - does that just mean the green arrows point from early genes to later-expressed genes in the same lineage? What are the blue lines? How were the dotted line groups constructed? What do the node colors mean?

p11 "is likely due to incomplete penetrance of the RNAi" - speculative. Could also be a partially penetrant null phenotype.

Figure 7 - please label colored cells and RNAi experiments on the figure, not just in the legend.

Figure 7Q (mentioned in the legend) was not part of my figure

Figure 8 - not proven in this study that "cell fate determinants" (CFDs) independently regulate temporal asymmetry and fate asymmetry. It could be that CFD's regulate fate and fate regulates temporal asymmetry, as well as more complicated organizations.

p16 "91wild-type" missing a space

p17 "pixel intensity of tissue marker expression was extracted from the raw images acquired with the channel other than that for the lineaging marker" awkward phrasing. Sufficient to say that tissue marker expression was extracted from the raw images - it is clear from the earlier methods that this marker was imaged in a second color.

The authors state (p17) that genes where "at least one pair of sister cells between wild-type and perturbed embryos ($p<0.05$)" were significantly different from WT. It was unclear whether and how the authors accounted for the multiple testing burden in this analysis. Also, the listed test (D'Agostino's K-squared test) is a test for normality, but the application appears to be to identify differences in mean division timing. How exactly was this test applied?

p14 - ending with the caveats of RNAi is very awkward. I suggest reorganizing to end by highlighting the utility of the dataset and how to access it.

Reviewer #3:

Summary and General Remarks

The authors present a very nice high throughput data set of embryonic cell division timings of C. elegans. To identify genes involved in the timing of cell divisions they focused on temporal

© European Molecular Biology Organization

asymmetries of division timing between sister cells (TADS). To do so, they performed an RNAi screen for genes, which are conserved with human and known to give an embryonic lethality or larval arrest phenotype. The aim of the screen was to identify genes required for TADS and to investigate whether different genes regulate TADS between cells of the same or different developmental fate.

They conclude two things from their analysis: 1) many of the same genes are required for TADS between cells with the same or different fates. 2) loss of TADS also leads to defects in cell migration and development.

The methodology of the paper is a combination of published techniques: RNAi screening and automated cell lineaging. This study highlights the role of a conserved set of genes in the differential timing of cell division between sister cells. These findings shed light on how cell division timing is controlled throughout embryogenesis; an important aspect of developmental biology we know surprisingly little about. In addition, the data archive promises a rich resource to investigate further aspects of cell cycle timing and differentiation in the future and is therefore of high interest to developmental and cell biologists.

Major concerns

(I) Regarding presentation of data: The aim of this study (as suggested by the title) is to compare which genes influence cell division timing during cell fate specification or tissue growth. Emphasis is put on genes that influence both, or only one of these types of divisions. But the presentation of the data (Figure 5) is not organized in a way that the reader could follow this comparison intuitively. The comparison might be easier if the genes in panels A and B were in the same row. Then it would be obvious which row is 'filled' in A AND B and which row is specific for only A OR B. (For detailed suggestions, please refer to Specific Comments, Figure 5).
(II) Regarding conclusion 1) I am missing a detailed analysis of one component or pathway that explains the differential role of one gene (set of genes) in cell division timing for cells of different fate or same fate. i.e. How is cell division timing regulated by these components? How does one gene regulate timing AND cell fate (is it through the same pathway or separate pathways)?
(III) Regarding conclusion 2) In the discussion it is stated: 'Our data on excretory cell specification also supported that temporal coordination of division paces is likely to facilitate proper cell migration during the proliferative stage of animal development.' From the presented data, the causalities are not clear. Does failure to set up cell fate affect temporal coordination and cell migration independently from each other? Does temporal coordination affect cell migration directly?

Comments and suggestions:

1)The authors could have taken greater care to avoid small mistakes in the manuscript; especially the referral to figures is imprecise and sometimes inaccurate (i.e. the figure or movie reference does not support the statement). Figure panels (A, B, C,...), not figures should be cited in the text! It is very difficult to follow the argumentation if the Figure references are not accurate.
2)Some hypothetically possible outcomes of gene depletion are not discussed: Did you also consider genes, where depletion produced a significant extension in DDT, or did that never happen? Could you add data on genes that changed the overall pace of development (in addition to changing TADS)?
3)Please define the phrases you use, e.g. What is a lineaging strain? (Any strain that expresses a histone marker? A strain that expresses a specific tissue marker?).
How do you define cell fate determinants? How do you define the gene class required for cell fate specification? (page 9, 2nd paragraph) (Based on known gene functions or your results?)
4)Could you comment what is special about the two sisters with the longest DDT?
5)The conclusions about the role of cell cycle (dominant or passive role, page 13) genes should be treated with caution. As mentioned, many of these genes lead to early embryonic arrest and were therefore not analyzed in this study. In the discussion it should be added that more detailed analysis (perhaps partial run-down) would be required to elucidate the role of cell cycle regulators.
6)Also, the authors describe cell specific phenotypes as if the genes were only depleted in those cells. Please discuss the possibility that some of the phenotypes could come from changes in their ancestor cells that were not picked up by focusing on differences in timing.

Presentation and style:
-In my opinion, the paper would benefit from a reduction of figures, which will encourage the authors to be more concise and focus their main conclusions.
-Only show Figures (and panels) that are explained in the main text and that actually support the given statement.

Specific comments and questions:

Introduction:
Page 4: You should mention that there is also a body of work that focused on the differences in cell division timings in the early stages of embryogenesis e.g. the Brauchle et al paper you cite. And discuss later how these results relate to your findings.

Main text:
Page 6, Validation of the pipeline:
-Why did you choose these genes for the validation?
-Which tissue markers were used for each of the RNAi experiments?
-Concerning: 'The application of our pipeline to pop-1 and lit-1 also allowed us to recapitulate the previously reported phenotypes.' What are these phenotypes? Why do you show data for pop-1 but not lit-1?
Page 6, bottom paragraph: Mention how many of the embryos were imaged with which tissue marker.

Page 7, second sentence: cite Supp. Table 4

Page 7, second paragraph: '...we required that the perturbed embryos developed at a relatively "normal" speed (see Materials and Methods)'. Which of the RNAi conditions lead to an overall decrease or increase in developmental time? The reference to M&M could be more specific.
The next sentence is not an example of this statement. Here you could also mention that embryos were excluded that arrested before 350 cells (such as many of the cell cycle RNAi conditions).
Page 8, top paragraph: reference to Figures not useful as these cells are not highlighted in Fig. 4A and Supp. Fig. 3A.
Page 8, 3rd paragraph:
- 'We referred to the genes of signal transduction, transcriptional regulation and chromatin modification as cell fate determinants.' In the next sentence you state that more genes are known to mediate cell fate specification, therefore I am confused by your definition of a cell fate determinant. (Do you mean the same genes/proteins when you use the term on page 3 in the Introduction?)
- Also you state that: 'the temporal asymmetry between the precursor of germline and body-wall muscle cells was primarily dictated by maternal factors and the Wnt/Src signaling pathway.' I guess this refers to P4-D? According to Figure 5, genes in the E3 ligase pathway also affected this.
- Refer to Fig. 5C for the last sentence.
Page 8, bottom paragraph: Should this section better be moved to the discussion? What do you conclude at this point from your analysis on the relationship between cell division timing and cell fate specification?
Page 9, second paragraph: Again, I find it difficult to know what exactly is meant by cell fate specification and cell fate determinants.
Page 9, bottom paragraph: Should this section better be moved to the discussion? What can be learned about the control of cell division timing from analyzing DDT during tissue growth?
Page 10, second paragraph: 23 genes affect the timing of the daughters of ABplapp. How/Why did you chose the presented 6 genes? It would be helpful to give the genes in the same order as in Fig. 5
Page 11, top paragraph: Concerning Figure 7, it is not obvious for ceh-43 (E) and arx-1 (H) that the division angles are different to wildtype. How was this quantified? Interestingly, in ceh-43 and arx-1 RNAi conditions, che-26 is still expressed. Could you comment if there could be a causal relationship between cell fate and the division angle of these cells?
Page 11, 3rd paragraph: Concerning: 'For example, a gene network controlling the specification of excretory cell was readily constructed based on its common roles in the regulation of temporal asymmetries and expression data (Figure 6K).' In the legend it says: 'genes identified for temporal asymmetry'. Do you know that all these genes that affect temporal asymmetry also are required for excretory cell specification? Is this denoted by the green arrows pointing to ceh-26 in Fig. 6K? Which gene expression data was used? Are the literature Refs. missing?

Discussion
Page 12, top paragraph: The grh-1 example is not clear. Is pharyngeal or cuticle an example for fate specification or temporal coordination?
Page 12, second paragraph: References missing: 'More specifically, the early regulatory factors that are asymmetrically segregated cell autonomously or by signaling events interact with three components of the cellular machinery, i.e., cell cycle, division spindle and gene expression. Interaction with the cell cycle and division spindle machinery sets the pace and angle of division respectively, while that with gene expression machinery drives the tissue-specific expression of fate determinants to mediate fate specification.'
Page 13, top: Please rephrase this sentence to be more clear: '... origin of cancer cells, and cell-specific deregulation in division timing is likely to be responsible for the tissue-specific origin of cancerous cells.'

Figures:
Figure 1: Panel C is redundant with Fig. 8A (if you choose to show both, keep the same order)
Figure 2: It would be useful to include the number of genes analyzed in the different steps of the flowchart, e.g. genes screened for DDT. Also check the number given for gene prioritization; in the text and Supp. Fig. 1 it is given as 822, not 865. Is the label for the cherry signal correct (H3 and H2B)? Which strain was used?
Figure 5: For ease of interpretation, I would assign a code (e.g. numbers) to the cells and add the same code into the lineage trees in Fig. 4A and Supp. Fig. 3A. As mentioned, I would suggest to align panels A and B to facilitate comparison between genes. It could be marked in this figure which genes are considered cell fate determinants. In panel A, there are two shadings used for transcription factors. Panels C and D could be presented in a table.
Figure 6: The lookup color bar is missing in H and I. In panel J it would be useful to assign gene names to the red dots. What do the colors and size of the balls mean in K?
Figure 7: A-H, which cell is red and which cell is blue?
Figure 8: Figure 8B should be replaced by a more concise presentation of the main findings (see also comment Supp. Fig. 6)

Supplemental:
Supplemental Fig. 2, related to Fig. 3: This Figure is supposed to support the statement for the validation of the pipeline, page 6. For this cbp-1 (Figure 2), pop-1, lit-1 and nhr-25 were characterized. But, only data from panel Supp. Fig. 2E is mentioned in the main text! Panels A-C: asymmetric migration of Aba and ABp are not referred to. Panel D: fate transformation after pop-1 RNAi is not discussed. If you discuss and show pop-1, you should also show lit-1.

Supplemental Fig. 3, related to Fig. 4: It should be mentioned in Fig. 4, that the ABp and P1 lineages can be found in Supp. Fig 3. Or move them to the main figure.
Panel A: for daughters of ABplaaap, differentiated cell and stem cell should be denoted, as in Fig. 4A
Is there a way to graphically present the following statement: TADS becomes apparent from the 6th and 3rd round of divisions in the sublineages of AB and P1, respectively (page 7, second paragraph).
Supplemental Fig. 5: This Figure is not informative. It is difficult to see which cells actually express CEH-26. It would be better if the cells expressing CEH-26 were stated in the text. And then refer to Fig. 6A for expression in ABplpappaa.
Supplemental Fig. 6: This Figure is related to Fig. 8B? For my taste, both, Fig. 8B and Supp. Fig.6 are too complicated to serve as summary figures. You should aim at presenting the main findings in one schematic Figure.

Supplemental Fig. 7: Only panel A is mentioned in the text. The example of mislocalization of cells after tads-1 RNAi is not discussed in the main text.

Supplemental Fig. 8: This figure is not properly referred to in the text and most of the defects described in the legend are not obvious from the images. Would it be better to refer to previous screens and cell biology papers that describe the defects instead of showing the images?

Supplemental Fig. 9: Which strain (from Table 3) is used for this analysis?

Supplemental Table 4: It would be great if this table also had a column for accumulated developmental time such as in Fig. 4A and Supp. Fig. 3A,B (0-223min) plus/minus errors.

Supplemental Table 6: Cited in the main text as: 'Surprisingly, 41 out of the 58 genes were also known to mediate cell fate specification directly or indirectly (Supplemental Table 6)'. Why are there 5 genes that do not have a reference?

Supplemental Table 7: Please double-check this list for completeness (e.g. air-2 is missing) and indication of 'early embryonic arrest'. e.g. dpl-1 is annotated as early embryonic arrest, but in Table 1 is says, it does not produce embryos.
Labeling of early embryonic arrest and red color coding is confusing. The black YES could be analyzed and were included in screen although they gave early embryonic arrest?

Trivial mistakes:

-Please check the manuscript carefully for typos, grammar and spelling mistakes (there are too many to be listed here)
-Page 13, line 6: 'a dominant and passive role' use active instead of dominant?
-Supplemental Table 3 is referred to as Supp. Table 2 on page 14
-Page 15, automated lineaging: was GPF and mCherry really collected simultaneously? Or rather consecutively?

1st Revision - authors' response 24 February 2015

(please see next page)

Reviewer #1:

This manuscript describes analysis of embryogenesis in C. elegans up to the 350-cell stage using time-lapse microscopy at 1.5-minute intervals and semi-automated cell lineaging using previously established software. The

authors measure cell division timings of WT and embryos treated with RNAi against 822 genes and compare differences in both cell division timing and cell lineages, with the aim of elucidating functional relationships between cell cycle timing and cell fate decisions that influence cell migration and patterning.

Overall, the authors do a reasonably thorough job of making the measurements that form the foundation of the study, and they have assembled a very nice dataset.

There are a number of concerns regarding the presentation, analysis, and interpretation of results:

1) The manuscript contains a huge number of grammatical and typographical errors. These make it more difficult to follow the narrative and require correction before the manuscript could be considered for publication. Some sentences are simply nonsensical. For example, on p. 8: "As described earlier, the anterior and posterior daughters of the two cells became terminally differentially and stem cells respectively."

We appreciate the reviewer for the tremendous amount of time in reviewing our manuscript and the remarkably thoughtful and constructive comments. We agree that there are substantial grammatical and typographical errors that should have been avoided before the submission and apologize for the inconvenience incurred. To improve the manuscript, both our colleagues and me have carefully read through the manuscript again and corrected all the identified errors including the one mentioned above. In addition, we had the manuscript proofread by professional editors who are native English speaker after revision of the content. We hope the revised manuscript can better satisfy your criteria for publication.

2) I have an issue with the nomenclature the authors have chosen to describe the phenomenon they are primarily interested in, i.e. "TADS (temporal asymmetry of division between sister cells)". The standard nomenclature for this kind of phenotype is "asynchrony", not "temporal asymmetry" (which is a term that does not make sense to me). For example, in the C. elegans embryo, there is a well-known asymmetry [a spatial descriptor] in the first mitotic cell division (which gives rise to a larger anterior AB cell and a smaller posterior P0 cell), as well as asynchrony [a temporal descriptor] of cell divisions of the two daughter cells (AB divides earlier than P0), and differences in orientation of division planes due to differential rotation of the spindle (P0 rotates and divides along the AP axis, whereas AB does not and divides transversely). The authors would do well to observe more commonly accepted standards of

vocabulary to describe these phenotypes, rather than come up with new and unnecessary nomenclature. As I understand it, the phenotype being addressed here is a difference in the length of the cell cycle between daughter cells (time from birth to division). Why not refer to this simply as a measure of asynchrony, which is the same thing and can be described/defined in whatever units are useful (here, minutes)? Similarly, cell division timings that are identical can simply be referred to as "synchronous". In addition, "DDT" is a well-known pesticide and it would be preferable to avoid using this acronym altogether.

To be consistent with the literature in terms of nomenclature, we changed "temporal asymmetry of division between sister cells" into "asynchrony of division between sister cells (ADS)". We reserved the "DS" because we need to be specific about the asynchrony between sister cells. An asynchrony may not necessarily mean the relationship between the immediate sister cells such as that between E2 and MS2 cells. We replaced "DDT" with "asynchrony" or ""ADS" where appropriate and changed "temporal asymmetry" into "asynchrony" throughout the manuscript.

3) In order to validate their pipeline, the authors examined cell lineage and tissue marker expression for genes that are known to produce embryonic defects when depleted. RNAi of cbp-1 results in extra cell divisions when compared to WT (Figure 3A). The authors also show that there is a net difference in division timing between the cbp-1 depleted embryos and WT. While this seems significant, its unclear if this net difference in cell division timing has a direct bearing on cell fate. Are there any conditional alleles/hypomorphic cbp-1 alleles available to test if differences in timing of cell divisions have a direct consequence on cell fate?

cbp-1 encodes a homolog of the mammalian transcription co-factor, CREB-binding protein (CBP), that has been shown to have histone acetyltransferase activity that is essential for its role in regulating transcription and cell fate specification. It has also been shown to positively or negatively regulate Wnt signaling targets depending on the cellular context in *Drosophila* (PubMed ID: 17410209). In addition to the broad defects in asynchrony that has not been reported previously, our data showed that depletion of CBP-1 eliminated the expression of tissue marker of PHA-4 and NHR-25 (see "phonics" database), but not that of the lineaging marker HIS-72::mCherry. In addition, E lineage pattern became MS like, indicating its broad roles in both cell fate specification and division synchrony. However, our data or even those

with conditional or hypomorphic allele may not be able to establish whether the defects in the synchrony play a direct or indirect role on cell fate formation. This will demand detailed biochemical and cellular analyses, which is beyond the scope of this paper. The main focus of this paper is to identify which genetic components are responsible for the observed asynchronies across cell types and within a single cell type during the proliferative stage of *C. elegans* embryogenesis.

4) The authors assert on p. 7 that a larger deviation in division timings later in embryogenesis suggests that cell cycle control might be relaxed relative to the early divisions.
a) Is there any other evidence in the literature to suggest this? If so then the authors can include a reference.
We could not find any such evidence in the literature.

b) This argument is not convincing without further investigation/elaboration. It appears from the manuscript that this statement refers to measures of absolute time of cell division with respect to a much earlier registration point. Thus, one would expect the total deviation over several cell divisions to reflect the cumulative deviation over several generations. If this is the case, then the statement cannot be supported without statistics to show that the deviation is larger than expected, given the historical trajectory.
We agree that part of the variations may be due to the cumulative effects from the previous generations. Since precise differentiation of the accumulative from "self" effects in division-timing variations is not feasible, we included a sentence in the main text, i.e., "It also remains possible that the increased variations in division timing during the later generations could be a product of the cumulative variations inherited from earlier generations".

If, on the other hand, the authors wish to refer to the deviation with respect to the length of the immediately preceding cell division, then they should change the presentation to reflect this (i.e. provide a comparison of variation in the length of earlier cell cycles vs. later cell cycles).
Either of the suggested approaches would essentially address the same question, and I suspect that the observation will not hold up.
See the description above.

5) The authors explain several of their filtering criteria in terms of "further

increasing selectivity" for the study. I don't understand the rationale for this. For example, the authors state (p. 7) that they only analyzed data for genes with at least a 50% reduction in DDT. Why not also look for increases in DDT? Or was this not observed?

A large scale screening of quantitative deviation in asynchrony at cellular level during metazoan development has so far not been reported in the existing literatures. To gain initial knowledge on how the division asynchrony is systematically established during early embryogenesis of metazoan development, we adopted the harsh filtering criteria in order to get hold of the most prominent players in the asynchrony regulation while minimizing the potential noises. We understand that we may miss some genes important for the asynchrony control for a particular cell, but our intention is to provide an initial survey on the genes involved in asynchrony control regardless of the cellular context. To facilitate the use of our data for the community, we published all the division timings and computed ADS in the website “Phenics” associated with this manuscript, including those increased ADS as described below. One can readily apply various filtering criteria to comb out the potential genes involved in control of the asynchrony between the cells of their interest.

We actually did the calculation for the increased ADS and upload them in “Phenics” database (which can be accessed by the Tab “Division timing” then “Sister cell division timing”, then searchable by gene name). A brief look at these genes show that some of them are those involved in cell adhesion (hmp-2, for example), generic gene transcription (splicing factors, for example), ATP production, etc. These embryos usually are commonly associated with severe defects in cytokinesis, which may partially explain the observed increase in ADS. However, a systematic analysis of these data has not been performed. This is because we made the assumption that the observed division asynchrony is a regulated cellular process in *C. elegans* by some factors which we are after; whereas in most other cases, initial rounds of embryonic divisions are synchronous, such as those in *Drosophila*, but how the asynchrony was regulated *in vivo* at systems-level remains largely unknown. Perturbation screening is supposed to recover the factors that control the asynchrony as observed during *C. elegans* embryogenesis.

6) Similarly, on p. 7, why were genes that did not show relatively "normal" speeds included in the analysis? Would these not be expected to produce interesting or relevant results? Is it just because they did not reach the 350-cell

stage at the desired time?

We assumed the question is ‘why were genes that did not show relatively "normal" speeds excluded in the analysis?’. We add this filter based on the assumption that depletion of many genes, for example, house-keeping ones, general factors in gene transcription or translation as well as those involved in ATP production may likely to produce overall slowdown of division paces and/or early embryonic arrest usually before 350-cell stage or cannot reach 350-cell stage at the desired time. Inactivation of these genes is not expected to cause differential effects on cell division timing that is manifested as ADS. Recent literature also demonstrated that overall slowing pace in development caused by low temperature or gene perturbation did not produce relative division timing between cells (Pubmed ID: 23863485). We listed the genes filtered out by these criteria in a new Supplementary Table S7.

7) On p. 9, it is stated that many genes giving rise to TADS of cells with the same fate are the same as those giving rise to TADS of cells with different fates. Do examples of two different kinds of TADS arise within the same lineages? I.e. does a mutation in Wnt signaling produce the two kinds of TADS in different generations of the same lineage, so that cells showing same-fate TADS later differentiate into different fates? This is important considering the sequence of events that occurs along the path of differentiation: chromatin remodeling and changes in gene expression often occur across several cell cycles as cells become progressively committed to a specific differentiated cell fate.

We do see the same gene that first regulates ADS during cell fate specification and later regulates the ADS during tissue growth with same lineal origin. An example of the gene is *pop-1* (Wnt signaling TCF) that regulates ADS between the two daughters of Ca that give rise to two different fates, i.e., anterior mainly to hypodermis while the posterior exclusively to body wall muscles. The gene later also regulates the ADS between the two daughters of the same lineage, i.e., Cap, which give rise to the same fate, which is body wall muscle (Figure 5 and Supplemental Fig 3A). There are quite a few such examples (Figure 5). However, due to the selection criteria for tissue growth, i.e., only those that give to the same fate are included in this category. Therefore, genes functioning in the opposite order, i.e., regulating same-fate ADS and later these sister cells differentiate into different fates, will not be identified.

8) On p. 9, there is a long discussion that is essentially a laundry list of results

and some vague comments about what they could mean. This discussion is very unsatisfactory. Also the role of SWI/SNF in T cells is noted; what species is this in? What kind of cells are these "T" cells?

We did find there are substantial confusions caused by poor logical flow and redundant descriptions here. We rephrased the relevant sentences and removed the sentence “The remaining genes appeared to be individually recruited by different cells to control the DDT between their daughters” in the first paragraph, which was repeated near the end of the second paragraph. The jargon "T" cell was replaced “tail seam cells” and species origin was also included.

9) On p. 11, the authors declare that morphogenesis defects later in development are causally related to earlier defects in cell division timing. This might be true, but could also be very indirect; this appears to be a conflation of correlation with causality between early, proximal phenotypes and much later, secondary / indirect phenotypes. For example, mutation in any of the PAR genes, which are important for cellular asymmetries and correct segregation of cell fate determinants in the one-cell stage, all give rise to "a disorganized mass of partially differentiated cells" with a variable terminal phenotype of several hundred cells (see various publications by Kemphues and Priess). Certainly there is some relationship between these, but many other things went wrong between the initial defects in the one-cell embryo and the terminal stage.

We agree with the point that such effect could be direct or indirect. In our original sentence “The results also suggested that defective temporal asymmetry during embryogenesis could be partially manifested as improper morphogenesis during late development”, we did not conclude that the defective division timing at early stage is fully responsible for the morphogenesis defects at a later stage by using “partially manifested”. We are also not certain whether the effect is direct or indirect either. Therefore, we are not explicit on that argument in terms of the causative relationship between the two. We removed “also” in the sentence, which seems improper logically.

10) Figure 1: B) Why do the authors show only 47 terminal cells, when they measure out to 350 cells?

This is mainly for the purpose of clarity of illustration here. A complete tree of 350-cell stage will be overwhelming when fitted here in a single row. A complete subtree of 350-cell stage is shown in Figure 4A (ABa and ABp) and

supplemental Figure 3A (P1) respectively.

C) The colors of the adjacent lineages do not reflect actual examples from B, which is confusing.
We have thought about the issue. On the one hand, the concept of DDT/ADS is uncommon to the community. We try to introduce the concept as explicit as possible. On the other hand, ADS is not obvious for cells at the 47-cell stage. Therefore, we decided to use the cartoon to illustrate the ADS concept.

11) Figure 3: B) What does the X-axis represent? Time? This is not described.
X stands for progeny of the blastomeres as indicated, which are ordered first by blastomere generation and second by alphabetical sequence of their name. For example, AB progeny was ordered as ABa, ABp, ABaa, ABap, ABpa, ABpp, ABaaa etc. We moved the name of blastomere into the differentially color coded areas to minimize the confusion. The figure legend was updated accordingly.

It also looks as if the cell cycle length in the cbp-1 RNAi condition is not only greater in magnitude on average, but that it shows much higher variability. It has previously been noted that many perturbations release phenotypic variation (e.g. Levy and Siegal). How does this factor into the authors' analysis and conclusions? Does RNAi of other genes result in similar observations?
Given the high dimension of our data, there are many aspects of the division timings that are beyond of our capacity to explore them in full. We agree the observation is a very interesting one, but it does not fit with our major question, which is to screen for the regulators that control division asynchronies at cellular resolution rather than those that appear to function as a phenotypic buffer or capacitor.
To increase the level of the insight of our data, we systematically computed the dispersion of division timings for both wild-type and perturbed embryos (see Materials and Methods) and identified a total of 66 genes as shown in Supplementary Fig S2. We did not factor them into our main conclusion in the abstract but discussed the results in the main text.

12) Figure 4: A) The legend and the colors and placement of the double-sided arrows do not match. There appears to be more than one error here, in both the text and the figure.
We are sorry about the discrepancies. The black arrows should denote sisters

giving rise to the same fate while the pink ones indicate sisters giving rise to the different fates. The mistakes are now corrected.

C) In at least one case, the difference in cell cycle length appears to be as long as 30 minutes, but I cannot see this in the dendrogram in (A). This is presumably because that particular difference occurs later, but it's so anomalous that it is interesting (though not discussed).

The longest difference in cell cycle length is between P4 and D, which is around 30 minutes. This can be observed in supplemental Figure 3A. The cell-pair list of the difference in cell cycle length between cells that is over 5 minutes is provided as Supplementary Table S4. A complete list of the cell-pairs with computed differences in cell cycle length can also be found in our online database “Phenics” by clicking “Division Timing” then “Sister cell division timing”. The output list can be ranked based on cell names, Sister Cell Cycle Length Difference (min) etc.

13) Figure 5: Parts (A) and (B) describe a higher level of resolution in terms than parts (C) and (D). I don't quite understand the rationale for lumping transcription factors, signaling factors, and chromatin modifiers together as "cell fate" genes. This is not necessarily the case, and doesn't strictly make sense to me. Why not just keep those three categories? Sure, some genes in each of the three classes can affect cell fate decisions, but that is certainly not the case across the board for all genes in these three classes. So, I find this somewhat misleading.

The figure 5C and 5D are changed to keep the original categories. In addition, Figure 5A and 5B are merged in order to facilitate the identification of the genes that are unique for “cell fate” or “tissue growth” or for specific cells. The revised figure 5C and 5D are moved into Supplementary data as Fig S6. We referred to the first five categories as cell fate determinant without necessarily meaning they always function as fate determinants, but the categories of these genes unexpectedly stand out in our screening. To find out a term that most appropriately represent the identified genes, we believe that “cell fate determinant” is one of the best descriptors for these genes, which are usually studied in the context of cell fate specification as seen in supplementary Table S8.

14) Figure 6: (J) The meaning of the red and green dots is not provided.

We are sorry for the missing descriptions here. Green and red dots denote

significant ($p<0.05$) and insignificant deviation ($p>0.05$) respectively from wild-type distributions, which are explicitly indicated in the figure. We replace the wording “ball” with “dot” which means the same. We also indicate the gene names with significant deviation on the figure.

(K) This is just a representation of the data in the STRING database for a subset of genes that the authors recovered in their screen. The connections are based on multiple types of functional connections that are not described at all here; conversely, functional associations that could be derived from the current study (such as quantitative measures of phenotypic similarity) are not represented. The meaning of the blue lines is not indicated, nor are the line weights; moreover the circles and green arrows are just ad hoc cartoons added by the authors. Referring to this as an excretory cell regulatory network, and to these groupings as "modules", seems a bit of a stretch.
We understand the naïve features of the “network” and the “module”. Given the content in this panel is not central to the major point we try to make, we move the panel into Supplementary data as Fig S10 and explain in more details of the figure in its legend. The intention of this figure is to illustrate that our quantitative data could be used a resource by the community to deduce a novel or enrich an existing regulatory network. We generate our data with the expectation that the scientific community will find them useful, for both those who are interested in a specific process or pathway as well as those who wish to build more comprehensive models of developmental regulation. We replace the panel with a new one as Figure 6K, which illustrates the significant deviations of division angle from wild-type by perturbation of the same set of genes.

15) The authors examined a subset of gene depletions (tbx-33, sptf-3, ceh-43, let526, snfc-5 and arx-1) that resulted in "broken" TADS in the excretory lumen. Further, expression of CEH-26, a secretory cell-specific marker, was eliminated in all cases except ceh-43 and arx-1 (Fig. 6 A-G), thus suggesting a coupled regulation of division timing and cell fate specification. Is the lack of CEH-26 expression a direct consequence of broken TADS? Could the authors test another tissue-specific marker?
Our data could not establish whether the functional relationship between broken TADS and CEH-26 expression is direct or indirect. We included this point in our Discussion section.
To further examine the coupling of regulation on division timing and cell fate

specification, we test another tissue specific marker as suggested, NHR-25, which is a hypodermis specific marker. We examine the marker expression in ABarpapp by depletion of two Wnt components, POP-1 and LIT-1 as well as three transcription factors, TBX-33, EGL-18 and HAM-1. All the depletions not only produce significant reduction in ADS (Figure 5), but also lead to elimination or reduced marker expression. The results are included as Supplementary Fig S8 (see also below).

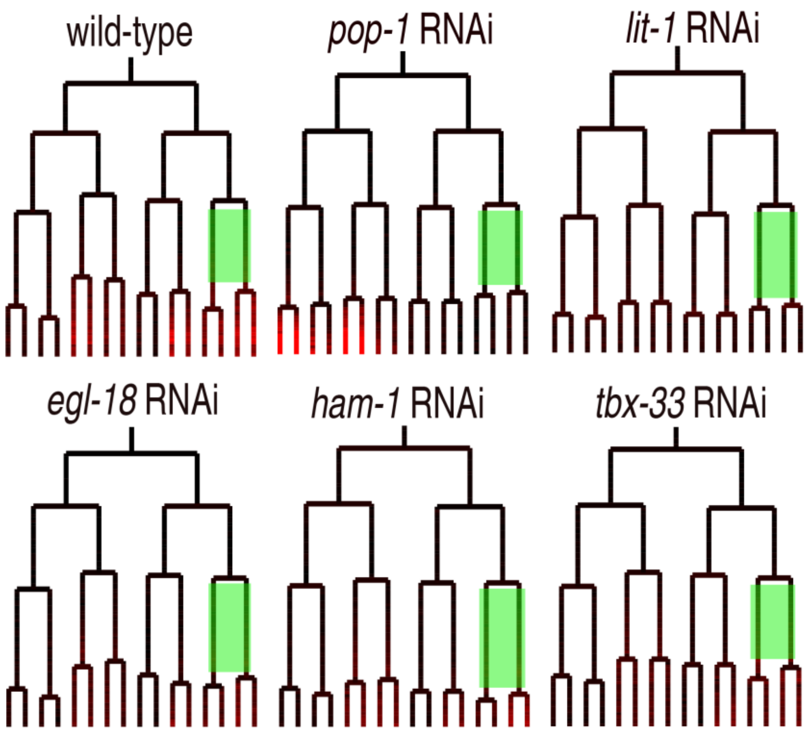

Lineal expression of NHR-25 (colored in red) in wild-type and RNAi embryos with genotypes indicated on the top. All trees are rooted with “ABarpa”. The two sister cells used for the calculation of ADS are shaded in green.

16) The authors note that they did not have reproducible results between replicates for a number of genes. Why not perform more than two experiments per gene in order to determine if there was simply an issue with a particular experiment? It's not that hard to repeat RNAi and time-lapse on more replicates. This would make it easier to determine if results could be repeatedly observed, even if RNAi was incomplete in some trials (and therefore elicited variable phenotypes).

We actually performed more than two replicate experiments per gene for over 130 genes (Supplemental Table 1). However, many of them still showed variable results, i.e., fewer than two replicates produce reproducible TADS phenotypes (see our screening criteria in Materials and Methods), suggesting that some of the RNAi tend to produce a spectrum of phenotypes at the cellular level. We speculate that many of such irreproducibility could be

resulted from intrinsic weakness of the RNAi, i.e., incomplete penetrance of the depletion. The reproducibility especially at the single-cell level is likely to be more prone to trivial changes of protein dosage than at the organism level. In addition, due to the highly demanding feature of the pipeline, we can only afford imaging of three and manual editing of two embryos per gene up to 350-cell stage for most genes. Repeating the entire pipeline would require substantial investment, including RNAi by injection, continuous 4D live-cell confocal imaging for at least 6 hours, running the lineaging pipeline for another half hour, human annotation for each embryo for another 1-3 hours per embryo depending on the image quality (Note that perturbation of some genes leads to substantial decrease in image quality).

17) The use of "ad hoc" on p. 13 is incorrect. I think the authors were looking for something like "de novo", but they should just say that they developed a new database.
“ad hoc” was removed or replaced with “new” throughout the manuscript.

18) Figure 7: (A-H) The description of the red and blue dots is incorrect in the legend.
Sorry for the mistake! It is corrected in the updated legend. The figure is moved into Supplementary data as Fig S9.

The orientation of divisions is more or less A-P in all cases shown; thus the argument that the orientation of division is seriously perturbed here is not at all convincing (in contrast the cells are clearly in the wrong positions in some panels). Why not make multiple measurements for all cases and do a more rigorous statistical evaluation, if the authors wish to assert that these changes are meaningful?
Thanks for the pointing out the weakness here. To examine the changes in division orientation in a more thorough way, we performed statistical analysis of the deviation of the division angles of the perturbed embryos from those of the 91 wild-type embryos. As a result, only three out of the six genes stand out as significant deviations from wild-type distribution relative to at least one reference plane. The remaining three demonstrate insignificant deviation relative to the wild-type, therefore not shown. We include these data as Fig 6K (see attached figure below) while moving the original Fig 6K panel and the entire Fig 7 into supplemental data as stated above.

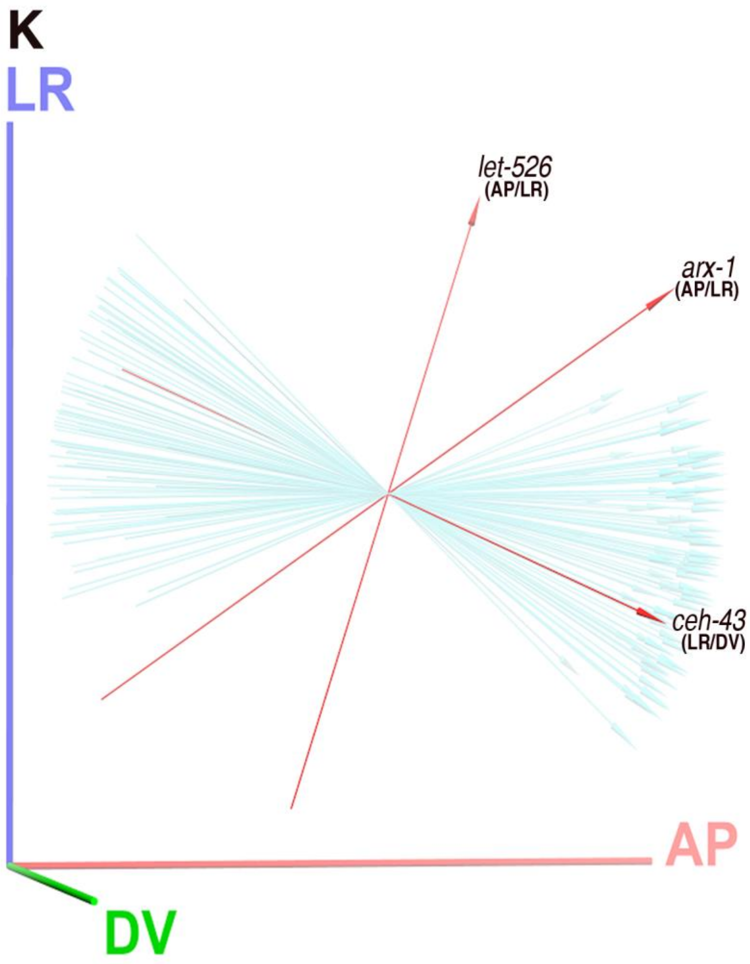

Shown are division angles of 91 wild-type (light blue) and three perturbed embryos with genes indicated (red). Division angles are calculated as that between the division orientation of ABplpappa relative to the three planes defined by axes between AP/LR, AP/DV and LR/DV. Only division angles that are significantly deviated from wild-type upon perturbation are shown. The reference plane for calculation of the angle deviation is indicated below gene name. Embryo axes are defined in the same way as that in panel J.

(J-M) The legend is inadequate, and arrows appear only in panel (O). In addition, the distinction between the "normal" cell bodies and the extra ones should be more explicit.

We now label the cell body of wild-type excretory cell with an arrow and update the figure legend as well.

19) The connection between spindle orientation and cell migration seems weak. These are different issues and a strong relationship between these is not clearly demonstrated. If the point relates to coordination between the asymmetric segregation of developmental determinants and orientation of the cleavage plane, then this point should be made more transparently. Again, I believe that the authors conflate correlation with causality, which raises some concern; for example, changes in either cell fate or timing can obviously change the environment in which cells find themselves, and thus influence cell migrations. But this is not an earth-shattering idea. What is the new message here?

We agree that our data do not provide direct evidence to establish directionality of the functional relationship between spindle orientation and cell migration. We therefore eliminate the original Figure 8B and supplemental Figure 6, which seem to be an overstatement of our results. The take-home message of our results is that it is the regulatory proteins commonly involved in cell fate specification that simultaneously control the division asynchrony observed during early embryogenesis. The genes involved in TADS also appear to affect cell migrations. However, the causal relationship between the regulation on division timing, cell fate specification and cell migration cannot be established solely with our data.

20) The final two sentences of the discussion should not be the concluding sentences of the entire paper; they belong more in the results or methods somewhere.

Thanks for pointing out the mistake! The two sentences were moved to the Materials and Methods under the section “dsRNA production and RNAi”.

21) Overall, the message of the paper (summarized in Figure 8) is not clear. Differences in cell cycle timing can occur between daughters of identical or different cell fates; in the former case, this can affect total cell proliferation and tissue size, and in the latter may (or may not) relate to the asymmetric segregation of developmental determinants. Mainly, the conclusions of the manuscript are largely phenomenological. The authors have done a large amount of work and collected a nice dataset, but they need to spend more effort to mine the resulting data and come up with some deeper insights into the results.

We did find there are many other insights than can be dug in our data. We admit we only mined very limited aspects of our data. For example, after reorganization of the Figure 5, we found that the control over ADS within a single fate of same lineal origin seems more likely to involve shared regulators. We also noticed that signaling pathways are more likely to demonstrate a coupled regulation over ADS for both tissue growth and cell fate specification; whereas the transcription factors show most prominent differential regulation between the two categories. We discussed this point in the main text. However, given the multiple-dimension feature of our data, complete mining of every dimension of our data may not be possible. We leave the data to the community to explore other biology of particular aspect of their interest while draw our conclusion on a specific aspect of developmental process, i.e., what

are the regulatory proteins that differentially time the division paces during proliferating stage so that cell divisions between many cells become asynchronous regardless of their cellular contexts.

The finding that mostly the cell fate determinants play a central role in such regulation regardless of cell fate contexts is significant. This is because that the asynchrony in division timing during tissue growth (within a single tissue type) or cell fate specification remains largely unknown, especially during the proliferative stage of embryogenesis. The temporal coordination seems to be coupled with cell fate specification and cell migration, but the exact relationships between these biological processes have not been established solely with the data provided in this project. Again, we have to admit that our data is high dimensional. We only mine a small portion of the full value of our dataset, and the remaining values of the data are left to the community for question-specific data mining.

Reviewer #2:

This manuscript describes an RNAi screen using state-of-the-art lineage tracing for phenotyping. The authors selected 822 genes based on a somewhat arbitrary but defensible set of criteria (reported to be essential, presence of human ortholog(s), mRNA expressed in embryos, lack of known "early embryonic arrest"). After filtering out genes with global effects leading to early arrest or global changes in rate, they focus on about 550 genes and find that RNAi depletion of about 80 of these causes changes in division timing asymmetry between one or more pairs of sister cells. The use of microinjection for RNAi is critical as previous work indicates this is likely to give stronger phenotypes than the more commonly used feeding strategy. Many of these hits are genes also known to be important for fate specification indicating that regulation of cell cycle asymmetry and fate are functionally linked. The novelty of some of these results is overstated in the current text (see below), but once the text is clarified and the data are made more accessible (see below) this will surely be a highly valuable resource for the community.

The main concerns I have are in the presentation and availability of the data. The quality of English is mediocre and the paper could benefit greatly from a detailed edit. I tried to access the website listed by the authors in the discussion to access the raw data, but it appeared that all access to the data required a login. This should be fixed. I have a fairly large number of mostly

minor questions and suggestions that should be addressed, listed below.

The restriction to the data access is now removed. We are also planning for contact with third party such as Wormbase for either hosting our data or establishing a link to our database to facilitate the data as a resource for the community. We have worked extensively on the English (manuscript is also edited by third-party professional service) and made data presentation as logical as possible.

The "early embryonic arrest" criteria was a bit unclear - what stage "arrest" was required for genes to be excluded using this criterion (or listed as "early embryonic arrest" in Supplemental Table 1)?

We are sorry for missing the detailed criteria in the main text. The only criterion used in the Supplemental Table 1 is the failure of our lineaging software StarryNite with trials of various parameters when the images from embryos perturbed with these genes were used an input. Very often few of these embryos could develop beyond 200-cell stage. We included the criterion at the bottom of Supplementary Table S1.

Some comparison with the recent work from the Bao lab (e.g. Du et al 2014) is needed. What genes if any were analyzed in common and how did the results compare?

In Bao's paper, they used five fate markers that almost covered all 14 sublibeages except ABala and P4, and they mainly focused on fate transformation after perturbation based on the altered marker expression. In our paper, we focused on cell division timings and used only a single fate marker for each perturbation in most cases. Therefore, some of the fate changes observed in their assay may not be certain in our case due to reduced resolution for fate assignment. Another major difference between Bao and our papers is that they used feeding while we used microinjection for conducting RNAi, which could produce phenotypic difference in terms of penetrance and scope. We included 18 out of his 20 genes in our dataset and a comparison of the cell fate phenotypes is included now as Supplementary Table S5.

p5 "We applied the pipeline to a total of 822 genes" - Only 762 genes have more than zero embryos and 750 have 2 or more according to Supplemental Table 1. If I understand the methods section this means that only 750 genes were actually analyzed. Figure 2, in contrast lists 865 genes. This should be clarified.

We are sorry about the inconsistency, which was likely due to the statistics done during different periods of the project. We checked our records and found we actually applied the pipeline (specifically mean RNAi, imaging and automated lineaging) to a total of 822 genes, 749 of which had gone through the pipeline with at least two replicates of edited embryos and were actually analyzed. 11 out of the remaining 73 had gone through the pipeline, but with a single replicate of the edited embryos because all of the other perturbed embryos demonstrated complicated defects such as defective cytokinesis or nuclear separation and early arrest (mostly before 50 cells). The remaining 62 out of the 73 genes had gone through RNAi and imaging, but not the step of automated lineaging, which is also due to early arrest of all the perturbed embryos or no embryos could be retrieved in multiple injected embryos. A florescence image from one of the imaged embryos at the last imaging time point was included in our "Phenics" website, which can be accessed under Tab "RNAi knockdown", then "Early Embryo Defects" from the homepage. We made all these number explicit in the Result section, as well as in Figure 2.

Some note about the consistency of observed phenotypes across replicates would be appropriate in the results section.
We include a few sentences on the consistence of observed phenotypes across replicates in the Result section

Supplemental table 1 - should note which genes were analyzed using reporters other than pha-4
The Table was updated and information on the tissue markers used for each gene was included. The marker information is also listed in our "Phenics" database for all perturbed embryos.

Figure 4, Supplemental table 4 - how do the division timings and standard deviations here compare with those previously reported by Bao et al 2008, Richards et al 2013?
We performed a pairwise comparison of average cell division timings up to 350 cells between Moore et al 2013 or Richards et al 2013 and ours. We found the Pearson correlation coefficient (r) is at least 0.987 in both cases. The results are included in the main text.

p17. How many embryos were excluded due to failure to reach 300 or 350 cells? What is the effect of removing such embryos - would many of these

have had defects in TADS?

59 genes were removed due to the cutoff, which were included in a new Supplementary Table S7. These embryos show an overall reduced developmental speed. We believe that most of these genes may be responsible for general developmental pace rather than cell specific pace. Most of these genes encode general components in gene transcription and translation or ATP production (mitochondrial). However, it remains possible that some of these genes may affect individual TADS. We computed TADS for each cell pair of all the perturbed embryos and deposited them in the “Phenics”. Those who are interested in a particular gene can analyze the data in a way that is tailored based on their interest.

A very noticeable writing problem is the repeated use of a very strange sentence construction e.g. pg 7 "we only included genes in our analysis, the depletion of which produced at least a 50% reduction in DDT compared with the average DDT of wild-type embryos between at least one pair of sister cells." This should be rewritten to avoid the ", the depletion of which...". For example: "we only included genes in our analysis whose depletion produced at least a 50% reduction in DDT compared with the average DDT of wild-type embryos between at least one pair of sister cells." This also occurs on pp8,9,10,12,17,26(legend title for Figure 5)

We made the changes accordingly throughout the manuscript.

In general the authors overuse the passive voice. For example "An online search database called Phenics is built" would be better as "We built an online search database called Phenics." This occurs throughout the manuscript.

We made the changes for most of the cases throughout the manuscript as appropriate.

Is the lack of core CC components in TADS regulation (p13) due to the fact that many of them lead to early embryonic lethal phenotypes so they were not considered?

This is partially true. For example, depletion of CDK-1 led to embryonic arrest at one-cell stage (http://phenics.icts.hkbu.edu.hk/index.php?r=embdefect/admin) and depletion of CDK-9 produced embryonic arrest at around 120-cell stage (http://phenics.icts.hkbu.edu.hk/index.php?r=searchdb/view&id=130531PHA4cdk9ip3). On the other hand, CDK-4 appears to be dispensable for regulation

of early embryonic cell divisions (http://phenics.icts.hkbu.edu.hk/index.php?r=searchdb/view&id=130604PHA4 cdk4ip1). It appears that most of other cell cycle components control overall cell division timing but not cell specific division asynchrony, which is consistent with recent literature, which has demonstrated that overall slowing pace in development caused by low temperature or gene perturbation do not produce relative division timing between cells (Pubmed ID: 23863485).

There are previous studies showing CC is influenced by fate determinants (eg Boeck et al, Developmental Biology (2011); Kaletta et al 1997, Lin and Priess (1995)). This result is thus not novel or "surprising" (as described in the abstract). The present study is useful not for identifying a novel role for fate regulators in controlling cell cycle but instead for pairing specific regulators with cells.
We agree with the point that literatures have reported overall CC or CC of particular lineage is influenced by cell fate determinants. However, a systematic screen for genes involved in division asynchrony (relative timing in cell division) between sister cells during proliferative stage of metazoan development has so far not been performed.

p3 "How cell division timings are regulated in vivo to ensure proper cell fate specification or tissue growth... is largely unknown." There is quite a bit of uncited literature on this topic in C. elegans including Bao et al 2008 (for which Dr. Zhao was a coauthor) and various others (Edgar and McGhee, Cell (1988); Nair et al, Development (2013); Budirahardja and Gonczy, Development (2008); Hebeisen and Roy, Development (2008))
We changed the description of “is largely unknown” into “remain elusive” and cited the missing literatures.

Abstract (p2): "cell division timings" - should be "timing" . Similarly "paces" should be "pace."
We made the changes accordingly.

Abstract - "Unprecedented spatiotemporal resolution" seems inaccurate given the earlier work from the Schnabel, Waterston, Bao and Murray labs. What is unique here is the scope in terms of the number of genes analyzed.
We changed “Unprecedented spatiotemporal resolution” into “single-cell resolution at 1.5-minute interval”.

p7 "embryonic stem cells" do not exist in C. elegans in the sense most readers would think of here. I'm not sure what cells the authors are referring to - perhaps Blast cells? "Stem cells" are also referred to in Figure 1C - not clear how the authors define such cells.
Yes, the meaning of "embryonic stem cells" is the same as “blast cell”. We changed the former into the "postembryonic blast cells" in both main text and the Figure.

A supplemental table listing the magnitude of differences (like Figure 5A/B but with the actual division timing numbers for WT and RNAi embryos and full p value) would be very useful.
We included the data as a new Supplementary Table S9. Note, that there are a few differences in significance levels for a couple of genes compared with the previous version when a few extra replicates were used for p-value calculation.

Figure 1A: Label for excretory cell (which points to the end of one of the canals) is misleading since the EC cell body is in the head, at the opposite end of the worm.
Due to the limited space in the head region for labeling “EC cell body”, we changed the label “excretory cell” into “excretory canal” which is the precise meaning of the indicated part.

Figure 2 A-X lettering is unnecessarily complicated. Would be better to label column 1 "A, A', A'..." Column 2 "B, B', B'..." etc.
We labeled the four columns from I to IV to avoid the unnecessarily complicated lettering.

The idea of using sister asymmetry as a phenotype is an interesting one, but not novel, as this quantity was analyzed in depth for WT embryos in Bao et al (2008). The authors should clarify what is gained by focusing exclusively on "TADS" as opposed to looking more broadly for CC defects.
In paper by Bao et al (2008), the authors mainly focused on characterization of overall reproducibility of cell division timings across cell lineages/fates and demonstrated that perturbation of some genes led to lineage/fate specific changes in division timing of a particular lineage. In this paper, we have undertaken a large scale of screening at cellular level and are trying to get hold of the genes that regulate individual division asynchrony that are reproducibly

observed not only between fates but also within the same fate at systems level. We believe that TADS is a manifestation of temporal coordination at micro/ cellular level.

Figure 3B makes it look as if cbp-1 RNAi causes broad changes in division timing, many of which wouldn't effect TADS.
It is true that cbp-1 RNAi caused dramatic changes in cell division timing and frequent defects in TADS for many cells, but the inactivation didn’t affect the TADS in all cases as shown in Figure 5. As raised by reviewer 1, it seems the protein functions as a buffering system or capacitor for cell cycle control. Reduction of its function appears to cause a relaxed control over cell division timing. We discuss this in the main text.

The definition of fate-symmetric vs. fate-asymmetric divisions and color coding in Figures 4 and S3 was confusing. The legend states that cells that give rise to the "same fate" are purple, and different fates are black but it appears this is reversed based on the color scheme.
We are sorry for the mistake and correct the errors accordingly.

And some tissues (such as pharynx and neuron) are extremely heterogeneous. For example MSpaap gives two "pharyngeal" daughters, yet one produces valve, neuron, marginal cells and cell deaths, while the other produces neurons and muscles. It isn't clear to me that treating these as two separate classes is meaningful. Are these classes partitioned among the genes (is a gene with a TADS defect in one "same fate" division more likely to have additional defects in other "same fate" divisions than in "different fate" divisions?)
We classified the cell fates mainly based on the expression patterns of our tissue marker, including PHA-4 (pharyngeal), NHR-25 (hypodermal) and HLH-1 (muscular), which was also used in Bao’s paper (2014). We agree there are some caveats associated with such classification.
To facilitate identification of shared and differential regulation of TADS between "same fate" divisions and "different fate" divisions, we merged the two parts into one and re-organized the genes to highlight the above features. We did observe that a gene with a TADS defect in one "same fate" division is more likely to have additional defects in other "same fate" divisions than in "different fate" divisions, but this is also dependent on their lineal origins. We added a few sentences for describing this in the main text.

Figure 4B - are the axes 91 embryos (label axes).
Both the x and y axes represent the pairwise correlation coefficient of division timing of all cells between individual embryos (out of the 91 wild-type). The figure legend was updated accordingly.

It appears there are a few major groups of embryonic timing profiles from this plot - what are the major differences between the top-left and bottom-right embryos?
Imaging of the 91 wild-type embryos was performed at various times. We dug the raw data and found that embryos imaged at the same period tend to group together, which could reflect the slight shift in temperature, to which the developmental speed is highly sensitive.

4C (legend) should read "DDTs that are bigger than five minutes"
We changed it into "Distribution of sister-pair count based on their asynchrony (in minutes) in wild-type 350-celled embryos." Note only the shaded asynchronies are over 5 minutes.

p8 - "suggesting that "differentially putting cell division on hold" may primarily rely on transcription factors rather than chromatin modifiers" conversational - rephrase
Rephrased into "suggesting that cell differentiation status may primarily rely on transcription factors rather than chromatin modifiers".

p9 "dyp-28" should be "dpy-28?"
Change is made accordingly.

p10 "broke TADS of one tissue" - the word "broke" is very informal. Maybe "disrupted" would be better?
Change is made accordingly.

p11 given the focus on the excretory cell maybe Figure S3 "ABpxp" should be part of the main body figure?
We merged the ABp tree of supplemental Fig.3A into the Figure 4A in the main text.

p10 - CEH-26. Images in Kolotuev et al 2013 suggest CEH-26/PROS-1 is

expressed in many more cells later in embryogenesis. This should be noted (better to say "CEH-26... is specifically expressed in the excretory cell prior to the 350-cell stage?")

“CEH-26 is a vertebrate Prox1 homologue that is specifically expressed in the precursor of excretory cell during embryogenesis” was changed into “CEH-26 is a vertebrate Prox1 homologue that is specifically expressed in the precursor of excretory cell prior to the 350-cell stage of embryogenesis”.

The difference in construct feature may contribute to the difference of the expression patterns. For example, we used GFP tagged fosmid for transgenes while Kolotuev used ceh-26 promoter fusion with GFP.

The "long-range migration" of the excretory cell stated in the text and implied by Figure 6H/I is somewhat misleading as that cell's "migration" is due more to collective cell movements related to gastrulation - it doesn't really migrate relative to its neighbors. To me this suggests excretory cell migration defects are likely due to larger defects in cell positions within the embryo (such as a failure of gastrulation).

We checked the cell migrations after perturbation of the genes shown in the Figure 6H/I in the time-lapse 3D movies in “Phenics” database. We found none of the perturbations produced severe defect in major collective cell movements such as gastrulation, and overall migration patterns such as symmetry in ABa and ABp daughters are still maintained. This demonstrated that the defects in "long-range migration" of the excretory cell was not due to early dramatic changes in cell migration such as gastrulation failure, but could be due to a more specific migration defects initiated later than gastrulation.

In addition it not obvious that 6I represents a significant migration defect compared with 6H.

At this point, we don’t know how to statistically call a significant deviation of a trajectory from the other. So the data (migration paths/trajectories) are largely qualitative rather than quantitative. However, the shift of end position in perturbed embryo is obvious during the late stage (I) compared with that in wild type embryos (H).

Figure 6J - can you label the points with significant deviations?

The meaning of the color was indicated in both the figure and its legend, i.e., red means $p<0.05$ and the green means $p>0.05$ (95% probability within the wild-type distribution with normality determined by D'Agostino's K-squared

test).

Figure 6K is intriguing. How was this network constructed?

The network was built with an online tools STRING using genes whose depletion significantly affects the TADS of excretory cell precursor, ABplpapp. STRING establishes gene interaction with known and predicted evidences. This panel was moved into supplementary Data as Supplementary Fig S10.

"Green arrows indicate gene expression based on lineal expression analysis" - does that just mean the green arrows point from early genes to later-expressed genes in the same lineage?

Green arrows are based on our data in Figure 6A-G. These genes are likely to be expressed in the same cells but probably earlier than CEH-26.

What are the blue lines? How were the dotted line groups constructed? What do the node colors mean?

Blue lines are generated by STRING with its thickness showing the strength of interaction between genes as established with known and predicted evidences. The dotted-line groups are arbitrarily generated by ourselves based on the pathway in which the genes are known to be involved. The color of the lines only serves to distinguish different groups. The figure legend is updated accordingly.

p11 "is likely due to incomplete penetrance of the RNAi" - speculative. Could also be a partially penetrant null phenotype.

We actually mean a partially penetrant null phenotype.

Figure 7 - please label colored cells and RNAi experiments on the figure, not just in the legend.

Cell names and RANi experiments are added accordingly. This figure is moved into Supplementary data as Fig S9.

Figure 7Q (mentioned in the legend) was not part of my figure

We are sorry about the mistake. The panel was shown as an independent figure as Fig S7 in the supplementary data. Thus the legend for Figure 7Q was removed.

Figure 8 - not proven in this study that "cell fate determinants" (CFDs)

independently regulate temporal asymmetry and fate asymmetry. It could be that CFD's regulate fate and fate regulates temporal asymmetry, as well as more complicated organizations.
This is a very good point, which we agree with. However, the role of CFDs in regulating TADS within the same tissue reflects its function in regulating temporal asymmetry but not fate asymmetry therein. If a CFD regulates TADS between cell fates, the exact relationship between its role in temporal and fate asymmetry remains to be determined.

p16 "91wild-type" missing a space
Change is made accordingly.

p17 "pixel intensity of tissue marker expression was extracted from the raw images acquired with the channel other than that for the lineaging marker" awkward phrasing. Sufficient to say that tissue marker expression was extracted from the raw images - it is clear from the earlier methods that this marker was imaged in a second color.
“the pixel intensity of tissue marker expression was extracted from the raw images acquired with the channel other than that for the lineaging marker” is changed into “raw fluorescence TIFF images acquired for tissue marker were used as an input for computing the intensity of the tissue marker expression for each cell with time using Acebatch as described”.

The authors state (p17) that genes where "at least one pair of sister cells between wild-type and perturbed embryos ($p<0.05$)" were significantly different from WT. It was unclear whether and how the authors accounted for the multiple testing burden in this analysis. Also, the listed test (D'Agostino's K-squared test) is a test for normality, but the application appears to be to identify differences in mean division timing. How exactly was this test applied?
Given the small sample sizes of treatment (usually 2 replicate RNAi embryos per gene), it is inappropriate to perform statistical analysis by simply comparing the mean TADS between the 91 wild-type and that of the two replicates. To accommodate this, we apply another test method which is commonly used for statistical comparison with a small sample size. This was described in the Materials and Methods and copied below. Some errors were also corrected. “To examine the statistical significance of the difference in ADS between wild-type and perturbed embryos with a relatively small sample size in perturbed embryos (usually two curated embryos per gene), D'Agostino's

K-squared test was performed as described previously (Moore et al. 2013, (Moore, Du and Bao. 2013, PubMed ID: 23861063) to evaluate the distribution of ADS of individual cells between 91 wild-type embryos. At least 75.8% of all examined ADS values passed the normality test with an alpha value of 0.05, which allowed us to assign the probability of ADS of a perturbed embryo outside the 95% and 99% confidence interval of the distribution of wild-type ADS as the p value, i.e., p<0.05 and p<0.01 respectively. A p value of 0.01 or 0.05 was assigned for Fig 5 if the ADS of at least two perturbed embryos were significantly smaller than that of the wild type embryos with p<0.01 and p<0.05 respectively."

p14 - ending with the caveats of RNAi is very awkward. I suggest reorganizing to end by highlighting the utility of the dataset and how to access it.
The last two sentences were moved to the Method section under dsRNA production and RNAi. We included a few sentence describing "how to access the data in the "phonics" database".

Reviewer #3:

Summary and General Remarks

The authors present a very nice high throughput data set of embryonic cell division timings of C. elegans. To identify genes involved in the timing of cell divisions they focused on temporal asymmetries of division timing between sister cells (TADS). To do so, they performed an RNAi screen for genes, which are conserved with human and known to give an embryonic lethality or larval arrest phenotype. The aim of the screen was to identify genes required for TADS and to investigate whether different genes regulate TADS between cells of the same or different developmental fate.

They conclude two things from their analysis: 1) many of the same genes are required for TADS between cells with the same or different fates. 2) loss of TADS also leads to defects in cell migration and development.

The methodology of the paper is a combination of published techniques: RNAi screening and automated cell lineaging. This study highlights the role of a conserved set of genes in the differential timing of cell division between sister cells. These findings shed light on how cell division timing is controlled

throughout embryogenesis; an important aspect of developmental biology we know surprisingly little about. In addition, the data archive promises a rich resource to investigate further aspects of cell cycle timing and differentiation in the future and is therefore of high interest to developmental and cell biologists.

Major concerns

(I) Regarding presentation of data: The aim of this study (as suggested by the title) is to compare which genes influence cell division timing during cell fate specification or tissue growth. Emphasis is put on genes that influence both, or only one of these types of divisions. But the presentation of the data (Figure 5) is not organized in a way that the reader could follow this comparison intuitively. The comparison might be easier if the genes in panels A and B were in the same row. Then it would be obvious which row is 'filled' in A AND B and which row is specific for only A OR B. (For detailed suggestions, please refer to Specific Comments, Figure 5).

In order to facilitate the comparison of differential regulation of TADS between tissue growth and fate specification by each pathway, we merged Figure A and B so that only one row was occupied by a single gene to facilitate the comparison of specific genes for each category. We also re-ordered the gene within each pathway to highlight the differential regulation of TADS between the two TADS categories. Overall, gene class showing the most prominent differentiation is transcription factor. Only 5 out 17 transcription factors have shared regulation on both categories; whereas 5 and 7 transcription factors are specific for tissue growth and fate specification respectively. In contrast, most of the components in Wnt and Notch pathways as well as maternal factors show shared regulation of ADS in at least one cell in each category (Figure 5). This is not surprising in that some transcription factors are known to function in a cell specific way. Intriguingly, two Hox genes, *ceh-13* and *nob-1* were only involved in TADS regulation during fate specification, which is expected based on the fact that Hox genes regulate cell fate specification in a position rather than tissue dependent manner. However, involvement of *nhr-25* only in regulation of TADS during breaking of fate asymmetry between neuron and hypodermis is unexpected because it is a hypodermis specific transcription factor, suggesting that the factor plays an important role in defining the hypodermis fate before its identity is established. We also included these

sentences in the Results.

In addition, we corrected a minor error of dual shading of TFs in Figure 5A and assigned tads-1 an independent functional pathway, Division asynchrony.

(II) Regarding conclusion 1) I am missing a detailed analysis of one component or pathway that explains the differential role of one gene (set of genes) in cell division timing for cells of different fate or same fate. i.e. How is cell division timing regulated by these components? How does one gene regulate timing AND cell fate (is it through the same pathway or separate pathways)?

Our data demonstrated that Notch pathway components mediated TADS during both tissue growth and cell fate specification. Surprisingly, the roles of Notch signaling in temporal regulation demonstrate precise correlation spatially with its reported roles in cell fate specification (Figure 5). The precise correlation between Notch's roles in temporal regulation and fate specification at cellular level demonstrates that the signaling pathway regulates development not only by controlling cell fate specification, but also by coordinating division timings in the context of a developing animal, an important aspect that is commonly ignored during the study of cell fate differentiation. Illustration of the exact relationship between division pace setting and cell fate specification will require extra biochemical and/or cellular analysis, which is beyond of the scope of this study. We included these sentences in the Results section under "Coordination of cell division timing during cell fate specification".

(III) Regarding conclusion 2) In the discussion it is stated: 'Our data on excretory cell specification also supported that temporal coordination of division paces is likely to facilitate proper cell migration during the proliferative stage of animal development.' From the presented data, the causalities are not clear. Does failure to set up cell fate affect temporal coordination and cell migration independently from each other? Does temporal coordination affect cell migration directly?

We agree that our data cannot establish the directionality of the interaction between the temporal coordination of division and proper cell migration or cell fate specification. We cannot nail down whether the interaction is direct or indirect either with the lineaging data only. To be precise for our claim, we rephrased the relevant sentences to reflect the caveat of the data. For example, we add the following sentence in the discussion "Further studies are

needed to define the directionality of the interaction among one another.”

Comments and suggestions:

1)The authors could have taken greater care to avoid small mistakes in the manuscript; especially the referral to figures is imprecise and sometimes inaccurate (i.e. the figure or movie reference does not support the statement). Figure panels (A, B, C,...), not figures should be cited in the text! It is very difficult to follow the argumentation if the Figure references are not accurate.
We are sorry for the mistake. We change the references to each figure panel explicitly and correct the mistakes in referrals to figure.

2) Some hypothetically possible outcomes of gene depletion are not discussed: Did you also consider genes, where depletion produced a significant extension in DDT, or did that never happen?
This is a good point. We did not mention these data in the main text because we made the assumption that the observed division asynchrony is a regulated cellular process in *C. elegans* by some factors which we are after; whereas in many other cases, initial rounds of embryonic divisions are synchronous such as those in *Drosophila*. Recent literature also demonstrated that overall slowing pace in development caused by low temperature or gene perturbation did not produce relative division timing between cells (Pubmed ID: 23863485). Perturbation screening is supposed to recover the factors that are responsible for the control over the asynchrony (relative division timing) as observed during *C. elegans* embryogenesis.
We actually did the calculation for the increased DDT and upload them in “Phenics” database (which can be accessed by the Tab “Division timing” then “Sister cell division timing”, then searchable by gene name). A brief look at these genes shows that some of them are those involved in cell adhesion (hmp-2, for example), generic gene transcription (splicing factors, for example), ATP production etc. These embryos are often associated with severe defects in cytokinesis, which may partially explain the observed phenotypes.

Could you add data on genes that changed the overall pace of development (in addition to changing TADS)?
We included an extra Table S7 that contains the list of the genes perturbation of which gives rise to overall slowdown in development.

3)Please define the phrases you use, e.g. What is a lineaging strain? (Any strain that expresses a histone marker? A strain that expresses a specific tissue marker?).

A lineaging strain is defined as the one that expresses both a lineaging marker and a tissue marker. A lineaging marker ubiquitously expresses two histone proteins fusions: H3.3 and H2B fused with mCherry or GFP. The expression of H3.3 is driven by his-72 promoter while the expression of the latter driven by a pie-1 promoter). A tissue marker is pha-4 (pharynx), nhr-25 (hypodermis) or hlh-1 (body wall muscle) that is also fused with GFP or mCherry but in the opposite order relative to that of lineaging marker when present in a single strain. The exact genotypes of the lineaging strains were listed in Table S3.

How do you define cell fate determinants?

We defined cell fate determinants as components of early maternal factors, E3 ligases, signaling pathways, transcription factors and chromatin modifier, which are the first five categories as shown in Fig 5. Generally speaking, these factors are usually characterized as cell fate determinants (see Table S8), though there are some exceptions. The five functional classes are the most prominent pathways that stand out in our screening and the cell fate determinants seem to be the most appropriate descriptor for all these factors.

How do you define the gene class required for cell fate specification? (page 9, 2nd paragraph) (Based on known gene functions or your results?)

Based on known gene functions.

4) Could you comment what is special about the two sisters with the longest DDT?

Sister cells with the longest asynchrony are between P4 and D, which are to exclusively develop into germline and body-wall muscle respectively (Supplementary Fig S3A). The extreme asynchrony between the two cells is likely due to the fact that P4 does not initiate zygotic transcription until it divides into P2 and P3 whereas the zygotic transcription starts in D much earlier. We include these sentences in the Result under section “A framework for screening genes regulating temporal asynchrony” and cited the relevant references.

5)The conclusions about the role of cell cycle (dominant or passive role, page 13) genes should be treated with caution. As mentioned, many of these genes

lead to early embryonic arrest and were therefore not analyzed in this study. In the discussion it should be added that more detailed analysis (perhaps partial run-down) would be required to elucidate the role of cell cycle regulators.
A sentence “A more detailed analysis is required to elucidate the role of cell cycle components in temporal regulation, for example, by conditional or cell cell-specific mutation or partial knockdown” was added in the Discussion section.

6) Also, the authors describe cell specific phenotypes as if the genes were only depleted in those cells. Please discuss the possibility that some of the phenotypes could come from changes in their ancestor cells that were not picked up by focusing on differences in timing.
We included the sentences “It should be noted that part of the observed defects in ADS of later generations could be the accumulative effects from previous generations.” in Discussion section.

Presentation and style:
-In my opinion, the paper would benefit from a reduction of figures, which will encourage the authors to be more concise and focus their main conclusions.

-Only show Figures (and panels) that are explained in the main text and that actually support the given statement.
We agree with the point and move Figure 7 into supplemental data. We also eliminate Figure 8B and Supplementary Figure 6 because the two figures are too complicated, which seems to be an over interpretation of our results.

Specific comments and questions:

Introduction:
Page 4: You should mention that there is also a body of work that focused on the differences in cell division timings in the early stages of embryogenesis e.g. the Brauchle et al paper you cite. And discuss later how these results relate to your findings.
We add various references on cell division timing in the Introduction and Results and discuss their relevance to our findings in Discussion section.

Main text:
Page 6, Validation of the pipeline:

-Why did you choose these genes for the validation?

These genes were previously demonstrated to produce lineage or cell fate transformation during embryogenesis upon perturbation.

-Which tissue markers were used for each of the RNAi experiments?

Mostly, PHA-4::GFP was used. NHR-25 and HLH-1 were also used in some cases. The detailed information on tissue marker used for each perturbation is now included in supplementary Table S1. It can also be found in the “Phenics” database.

-Concerning: 'The application of our pipeline to pop-1 and lit-1 also allowed us to recapitulate the previously reported phenotypes.' What are these phenotypes? Why do you show data for pop-1 but not lit-1?

We add the data on lineage transformation of LIT-1 depletion in Supplementary Fig S3 C and G.

We also include the sentence “i.e., inactivation of the former led to homeotic fate transformation of MS to E while inactivation of the latter produced the opposite phenotype (Supplementary Fig S3 C and G)” in the relevant part of the Result.

Page 6, bottom paragraph: Mention how many of the embryos were imaged with which tissue marker.

In the sentence “We collected three replicate movies per imaging session with a single tissue marker for each perturbed gene”, we described that 3 embryos were imaged with each tissue marker. However, for a few genes, we did the imaging sequentially with different tissue markers for a more thorough evaluation of fate transformation.

Page 7, second sentence: cite Supp. Table 4

Change was made accordingly.

Page 7, second paragraph: '...we required that the perturbed embryos developed at a relatively "normal" speed (see Materials and Methods)'. Which of the RNAi conditions lead to an overall decrease or increase in developmental time?

We define the abnormal speed in Materials and Methods as embryos that did not satisfy the following criteria, namely when a perturbed embryo was not able to develop into 300 cells at the last editable time point or could not reach

350 cells up to the last imaging time point in a curated embryo, which was assumed as “abnormal” developing speed. The genes whose perturbation led to “abnormal” developing speed are listed in Table S7.

The reference to M&M could be more specific.
We changed the sentence “(see Materials and Methods)” into “(see “Quantitative analysis of cell division timings and marker gene expression” in Materials and Methods)”.

The next sentence is not an example of this statement. Here you could also mention that embryos were excluded that arrested before 350 cells (such as many of the cell cycle RNAi conditions).
We rephrased the sentence accordingly.

Page 8, top paragraph: reference to Figures not useful as these cells are not highlighted in Fig. 4A and Supp. Fig. 3A.
The mentioned two sister pairs are now highlighted with an arrow and arrowhead in Fig 4A (Note, part of original Fig S3A (ABp lineage tree) was merged into Fig 4A).

Page 8, 3rd paragraph:
- 'We referred to the genes of signal transduction, transcriptional regulation and chromatin modification as cell fate determinants.' In the next sentence you state that more genes are known to mediate cell fate specification, therefore I am confused by your definition of a cell fate determinant. (Do you mean the same genes/proteins when you use the term on page 3 in the Introduction?)
We are sorry about the confusions here. We refer to the cell fate determinant as any regulatory factors that mediate fate specification. By applying this criterion to Fig 5, we referred to the first five categories as cell fate determinant without necessarily meaning they always function as fate determinants, but the categories of these genes unexpectedly stand out in our screening. To find out a term that most appropriately represent these identified genes, we believe that “cell fate determinant” is one of the best descriptors for these genes, which are usually studied in the context of cell fate specification as seen in supplementary Table S8.

- Also you state that: 'the temporal asymmetry between the precursor of germline and body-wall muscle cells was primarily dictated by maternal factors and the Wnt/Src signaling pathway.' I guess this refers to P4-D? According to Figure 5, genes in the E3 ligase pathway also affected this.
Yes, it refers to P4-D. Sorry for the inexplicit description! We indeed miss the genes in the E3 ligase pathway and now describe them in the main text.

- Refer to Fig. 5C for the last sentence.
Change is made accordingly.

Page 8, bottom paragraph: Should this section better be moved to the discussion? What do you conclude at this point from your analysis on the relationship between cell division timing and cell fate specification?
We retained the paragraph but had it reorganized and rewritten in order to be explicit about our conclusion here.

Page 9, second paragraph: Again, I find it difficult to know what exactly is meant by cell fate specification and cell fate determinants.
See explanations above. Cell fate specification means cell division with each daughter gives rise to distinct cell type (fate) while tissue growth means cell division with both daughters developing into a single cell type.

Page 9, bottom paragraph: Should this section better be moved to the discussion? What can be learned about the control of cell division timing from analyzing DDT during tissue growth?
We reorganized the paragraph and included the sentences in order to be explicit about our conclusion here.

Page 10, second paragraph: 23 genes affect the timing of the daughters of ABplapp. How/Why did you chose the presented 6 genes? It would be helpful to give the genes in the same order as in Fig. 5
We mainly focused on the genes that are transcription factor or chromatin modifier or those known to be relevant to excretory cell development except for those of signaling pathways. We included the criteria for the gene selection in the main text and re-ordered the genes according to Fig 5.

Page 11, top paragraph: Concerning Figure 7, it is not obvious for ceh-43 (E) and arx-1 (H) that the division angles are different to wild-type. How was this

quantified? Interestingly, in ceh-43 and arx-1 RNAi conditions, che-26 is still expressed. Could you comment if there could be a causal relationship between cell fate and the division angle of these cells?

We agree that the claim on division angle here is weak because we did not quantify the angles for statistical analysis here. Also, the deviation in division angle in 3D may not be obvious in a 2D figure. To ascertain the significant changes in division orientation, we performed statistical analysis of the deviation of the division angles of the perturbed embryos from those of 91 wild-type (For details, see updated Materials and Methods). As a result, only three out of the six genes (let-526, arx-1 and ceh-43) show significant and reproducible deviations from wild-type distribution relative to at least one reference plane. The remaining three demonstrate either irreproducible deviations between replicates or insignificant deviation. We include these data as Fig 6K (also attached below) while moved the original Fig 6K panel and the entire Fig 7 into supplementary data. We updated the relevant part in the manuscript.

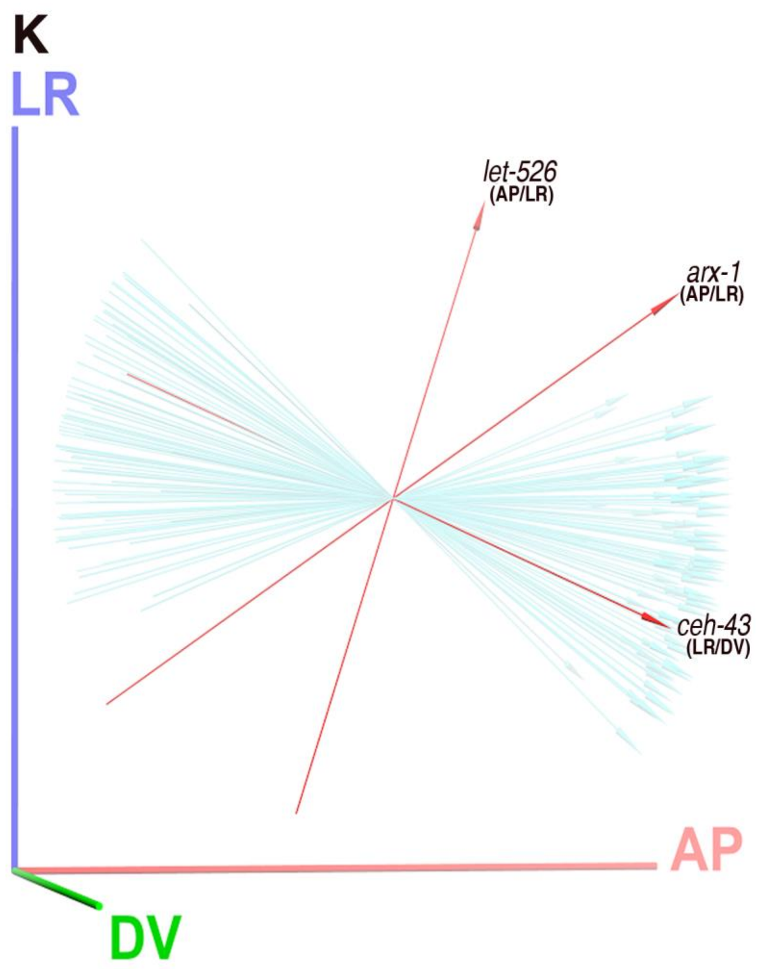

Shown are division angles of 91 wild-type (light blue) and three perturbed embryos with genes indicated (red). Division angles are calculated as that between the division orientation of ABplpappa relative to the three planes defined by axes between AP/LR, AP/DV and LR/DV. Only division angles that are significantly deviated from wild-type upon perturbation are shown. The reference plane for calculation of the angle deviation is indicated below gene name. Embryo axes are defined in the same way as that in panel J.

Page 11, 3rd paragraph: Concerning: 'For example, a gene network controlling the specification of excretory cell was readily constructed based on its common roles in the regulation of temporal asymmetries and expression data (Figure 6K).' In the legend it says: 'genes identified for temporal asymmetry'. Do you know that all these genes that affect temporal asymmetry also are required for excretory cell specification? Is this denoted by the green arrows pointing to ceh-26 in Fig. 6K? Which gene expression data was used? Are the literature Refs. missing?

We don’t know whether these genes are required for excretory cell specification or not when we prepared the figure, but 4 out of the six were demonstrated to be required for its specification based on the expression analysis shown in Fig 6A-G.

Those denoted by the green arrows indeed point to ceh-26 in Fig. 6K from the data shown in Fig 6A-G. We updated the figure legend accordingly to make the meaning explicit. This panel was moved into supplementary data as Fig S10 after the update.

Discussion

Page 12, top paragraph: The grh-1 example is not clear. Is pharyngeal or cuticle an example for fate specification or temporal coordination?

The grh-1 is for illustration of its sole role in temporal coordination in the case of pharyngeal tissue development (Fig 5), but the gene has been shown to be required for fate specification during cuticle synthesis (function of hypodermis), which is not picked up here in temporal control of hypodermis, suggesting that it is differentially used for temporal and fate control depending on the cellular context.

Page 12, second paragraph: References missing: 'More specifically, the early regulatory factors that are asymmetrically segregated cell autonomously or by signaling events interact with three components of the cellular machinery, i.e., cell cycle, division spindle and gene expression. Interaction with the cell cycle and division spindle machinery sets the pace and angle of division respectively, while that with gene expression machinery drives the tissue-specific expression of fate determinants to mediate fate specification.'

We rephrase the sentences and this is just our speculation, so no reference is added.

Page 13, top: Please rephrase this sentence to be more clear: '... origin of cancer cells, and cell-specific deregulation in division timing is likely to be responsible for the tissue-specific origin of cancerous cells.'

The sentence was rephrased in the main text as “Given the conservation of the identified genes, our findings on cell-specific regulation of division timings by various regulatory proteins could provide clue to the tissue-specific origin of cancerous cells”.

Figures:

Figure 1: Panel C is redundant with Fig. 8A (if you choose to show both, keep the same order)

We chose to show both and change the order in Panel C of Fig 1 into the same as that in Fig 8 (now Fig 7 due to moving of the original Fig 7 into supplementary data).

Figure 2: It would be useful to include the number of genes analyzed in the different steps of the flowchart, e.g. genes screened for DDT. Also check the number given for gene prioritization; in the text and Supp. Fig. 1 it is given as 822, not 865. Is the label for the cherry signal correct (H3 and H2B)? Which strain was used?

We are sorry about the mistake. We include the corrected numbers of genes on each step (Figure 2 and Figure S1) accordingly. The cherry label of H3::mCherry is changed into H3.3::mCherry (which is HIS-72::mCherry). The strain RW10425 was used for the lineaging.

Figure 5: For ease of interpretation, I would assign a code (e.g. numbers) to the cells and add the same code into the lineage trees in Fig. 4A and Supp. Fig. 3A. As mentioned, I would suggest to align panels A and B to facilitate comparison between genes. It could be marked in this figure which genes are considered cell fate determinants. In panel A, there are two shadings used for transcription factors. Panels C and D could be presented in a table.

Thanks for pointing out the nice way for improving the data presentation! The figure is updated by merging of panel A and B and the genes within each functional category are re-ordered to facilitate the identification of regulatory proteins that show differential control over division timing between tissue growth and fate specification. As a result, transcription factors turn out to be the most prominent category showing the differential regulation. We include the new finding in the main text.

A code is assigned in the updated Fig 5 and labeled accordingly in Fig 4A and Supplementary Fig S3A. Panels C and D of the original Fig 5 were presented as supplementary Fig S6.

Figure 6: The lookup color bar is missing in H and I. In panel J it would be useful to assign gene names to the red dots. What do the colors and size of the balls mean in K?
Color changes gradually with accumulative developmental timing in H and I. A color-coded time scale is included for both panels. Gene names for red dots are now included in the updated Fig 6J. Meaning of the dot color is defined in the figure legend. The ball size is not relevant.

Figure 7: A-H, which cell is red and which cell is blue?
The figure is moved into supplementary data as Fig S9. Red and blue cells represent “ABplpappa” or its daughters and “ABplpappp” or its daughters respectively, and the coloring info was included in the update figure legend. Excretory cell is the granddaughter of “ABplpappa”.

Figure 8: Figure 8B should be replaced by a more concise presentation of the main findings (see also comment Supp. Fig. 6)
We eliminate original Figure 8B and Supp. Fig. 6 because first, it is too complicated; second, we feel it seems an over interpretation of our result. We therefore update the original Fig 8A to reflect our points in a more precise way (now as Fig 7 due to original Fig 7 was moved to Supplementary data).

Supplemental:
Supplemental Fig. 2, related to Fig. 3: This Figure is supposed to support the statement for the validation of the pipeline, page 6. For this cbp-1 (Figure 2), pop-1, lit-1 and nhr-25 were characterized. But, only data from panel Supp. Fig. 2E is mentioned in the main text! Panels A-C: asymmetric migration of Aba and ABp are not referred to. Panel D: fate transformation after pop-1 RNAi is not discussed. If you discuss and show pop-1, you should also show lit-1.
We included the data on lit-1 RNAi in supplementary Fig S3 C and G and made the reference to all the panels.

Supplemental Fig. 3, related to Fig. 4: It should be mentioned in Fig. 4, that the ABp and P1 lineages can be found in Supp. Fig 3. Or move them to the main

figure.

We only moved ABp lineage in Supp. Fig 3 to Fig 4A due to the use of precursor of the excretory cell later and space limitation. We made the reference as appropriate in the figure legend.

Panel A: for daughters of ABplaaap, differentiated cell and stem cell should be denoted, as in Fig. 4A

We highlight the daughters of ABplaaap with an arrow in the update figure.

Is there a way to graphically present the following statement: TADS becomes apparent from the 6th and 3rd round of divisions in the sublineages of AB and P1, respectively (page 7, second paragraph).

We included a supplementary Fig S4C (also attached below) showing the count of cell with a TADS over 5 minutes in AB and P1. After a detailed examination of Table S6, it turns out that 5th and 4th round of division in AB and P1 lineage respectively when TADS becomes apparent. The mistake is corrected in the main text.

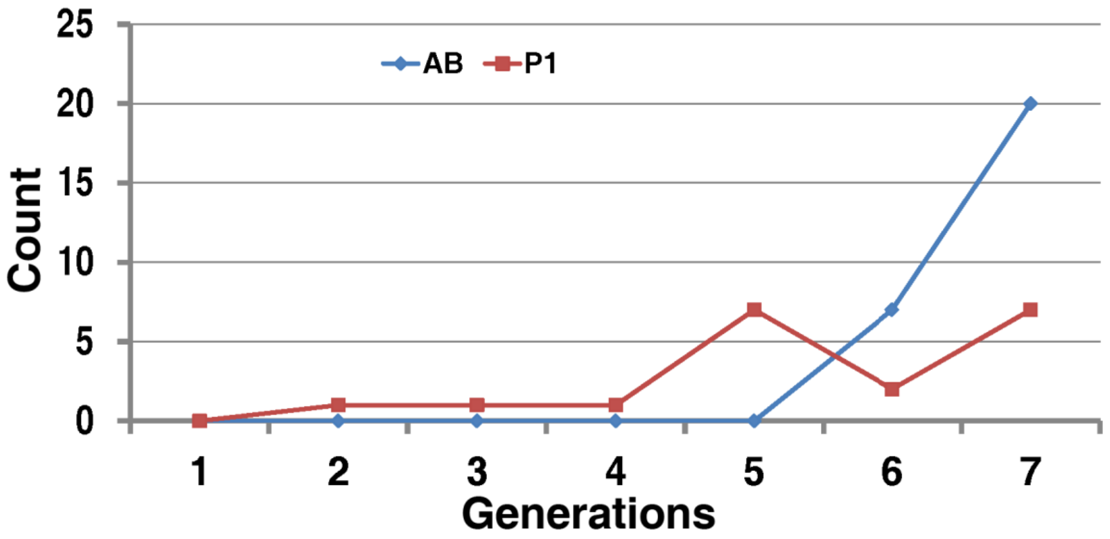

Count of cells (vertical axis) whose ADS is longer than five minutes over generations (horizontal axis). Daughters of AB and P1 lineage are differentially color-coded.

Supplemental Fig. 5: This Figure is not informative. It is difficult to see which cells actually express CEH-26. It would be better if the cells expressing CEH-26 were stated in the text. And then refer to Fig. 6A for expression in ABplpappaa.

The figure legend was rewritten to make the meaning explicit. A reference to Fig 6A was made in the text. A typo of “Show” is corrected as “Shown”.

Supplemental Fig. 6: This Figure is related to Fig. 8B? For my taste, both, Fig. 8B and Supp. Fig.6 are too complicated to serve as summary figures. You should aim at presenting the main findings in one schematic Figure.
We eliminate both Figure 8B and Supp. Fig. 6 as described above and update Figure 8A to reflect our points in more concise way (now as Figure 7 due to original Figure 7 was moved to Suppl data).

Supplemental Fig. 7: Only panel A is mentioned in the text. The example of mislocalization of cells after tads-1 RNAi is not discussed in the main text.
We added a few sentences and discussed the cell migration defect by tads-1 RNAi and made the reference to them in the main text.

Supplemental Fig. 8: This figure is not properly referred to in the text and most of the defects described in the legend are not obvious from the images. Would it be better to refer to previous screens and cell biology papers that describe the defects instead of showing the images?
We removed the figure and made the reference to the previous screening on cell biology defects as appropriate.

Supplemental Fig. 9: Which strain (from Table 3) is used for this analysis?
RW10425 was used for this analysis. To generate lineal expression of lineaging marker expression (Supplemental Fig. 9), instead of TIFF images derived from tissue marker, but those derived from lineaging marker (H2B::mCherry, H3.3::mCherry) were used as an input for Acebatch. We included this information in the end of section of “Automated lineaging, profiling of tissue marker expression and manual curation of the automated output” under Materials and Methods.

Supplemental Table 4: It would be great if this table also had a column for accumulated developmental time such as in Fig. 4A and Supp. Fig. 3A,B (0-223min) plus/minus errors.
We included accumulative developmental time and its standard deviations in the updated Table S4.

Supplemental Table 6: Cited in the main text as: 'Surprisingly, 41 out of the 58

genes were also known to mediate cell fate specification directly or indirectly (Supplemental Table 6)'. Why are there 5 genes that do not have a reference? Sorry for missing the references! We included the missed reference in the updated Supplemental Table S8.

Supplemental Table 7: Please double-check this list for completeness (e.g. air-2 is missing) and indication of 'early embryonic arrest'. e.g. dpl-1 is annotated as early embryonic arrest, but in Table 1 is says, it does not produce embryos.
We included *air-2* in the table S9. The criterion for selection of cell cycle related gene is a bit arbitrary, so a “complete” list may not be feasible. We also highlight *dpl-1* with a “^” to indicate the exceptional case.

Labeling of early embryonic arrest and red color coding is confusing. The black YES could be analyzed and were included in screen although they gave early embryonic arrest?
We replaced red labeling with a “#” and labeled the corresponding text into black. Yes, the black YES could be included in the screen if we relax our screening criteria.

Trivial mistakes:

-Please check the manuscript carefully for typos, grammar and spelling mistakes (there are too many to be listed here)
We had the manuscript proofread again by colleagues and English native speakers. This type of errors should be minimal now.

-Page 13, line 6: 'a dominant and passive role' use active instead of dominant?
Change was made accordingly.

-Supplemental Table 3 is referred to as Supp. Table 2 on page 14
Change was made accordingly.

-Page 15, automated lineaging: was GPF and mCherry really collected simultaneously? Or rather consecutively?
GFP and mCherry images were indeed collected consecutively and the change was made accordingly.

2nd Editorial Decision 17 April 2015

Thank you again for submitting your work to Molecular Systems Biology. I apologize for the delay in getting back to you, which was due to the late arrival of one of the referee reports. We have now heard back from the three referees who agreed to evaluate your revised manuscript. As you will see from the reports below, the main concerns of the reviewers have been satisfactorily addressed. However, they still list some remaining issues that we would ask you to address in a revision of the manuscript.

Overall, the referees provide clear and constructive recommendations. We would like to raise your attention to the point raised by reviewers #1 and #2, which refers to the need to improve text quality and clarity and make sure to avoid overstatements and over-interpretation of the results. Moreover, in line with comment #15 of reviewer #1, we think that the dataset/resource should be highlighted in a separate paragraph.

Reviewer #1:

This resubmission by Ho et al. is greatly improved over the original version. Overall, this is a very nice piece of work. The authors have gone to great lengths to carefully address most of the comments from the first round of reviews, resulting in a greatly improved presentation. The narrative has been extensively revised and now reads much better, and the authors have made efforts to tighten and focus the interpretation and discussion of results. I have a few outstanding concerns, enumerated below, which I expect could be relatively easily addressed.

In particular, I would still like to see the Discussion tightened and streamlined in order to bring out the main conclusions from the study. There is a lot of data here that provides a nice foundation for downstream mechanistic studies. However, the authors do a lot of dancing around the fact that, without further analysis, the true functional dependencies between the factors they uncover in their screen cannot be clearly discerned. It would therefore be useful to simply state this, and then elaborate on what they think is a plausible scenario. But it needs to be clear that further analyses will be required to establish a mechanistic understanding of how asymmetric cell divisions and cell division timing asynchrony are coordinated.

1) On page 7, a reference should be included to introduce the idea of a phenotypic "buffer" or "capacitor", since this concept has not been introduced prior to this point in the manuscript.

2) Beginning on page 13 in the Results, and throughout the Discussion, there again appear a large number of grammatical errors and weird sentence constructs. These sections require further editorial attention before they could be considered suitable for publication.

3) On page 13, the authors persist in trying to use the word "asymmetric" to refer to both fate specification and division timing. As noted previously, "asymmetry" is a spatial concept, whereas "asynchrony" is a temporal one. This needs to be corrected.

4) The discussion on excretory cell specification on pp. 13-14 is confusing and should be revised. The observations are that four genes influence both asynchrony and cell fate, whereas two affected asynchrony more than fate. Then, a discussion of Notch and Wnt signaling ensues (though the data for Notch are not presented here or cited from the literature). The authors alternately note that different results indicate either "coupled" regulation of the two processes or "discrete" regulation.

The difficulty I have with the way this section is written is the apparent implication that the results are contradictory as to whether these processes are separable for a given cell type; instead I suspect that the intent is to comment upon the level of regulation at which these processes might be coupled, as revealed by RNAi of different gene products. This really does not come across clearly, however, and these comments regarding interpretation are probably best left to the Discussion.

This kind of expository problem is repeated several times in the following sections. The way the text

comes across, the authors wish to have it both ways: the regulation appears to be both coupled and separable. Without further dissecting the pathways, effectors, and dependencies here, this comes across as very cloudy. The narrative would benefit from some clarification on these points.

5) P. 15: "relative positions of most perturbed embryos were significantly shifted compared to the wild-type embryos" does not make sense. Are the embryos themselves in a different place? Presumably the authors intend to refer to specific cells/cell divisions rather than embryos per se here.

6) P. 15: Why does a cell with an extra nucleus imply defective fate specification? Usually this signifies a failure of cytokinesis (with indirect downstream effects, such as failure in the asymmetric segregation of cell determinants pursuant).

7) P. 15: What are "thorn-like" canals?

8) P. 15 and Figure S10: I really cannot see how a STRING representation of a bunch of genes that are involved in specification of a particular lineage results in "a putative gene network controlling [insert cell type here] specification" - particularly one that can be interpreted as being "initiated by maternal factors followed by Wnt and Notch pathways, and further relayed by transcription factors and chromatin modifiers". While a plausible scenario, this really seems like over-interpretation of the data.

In the supplementary figure, the composition of the STRING network needs to be more clearly described. The STRING database gets updated regularly, and the most current version is proprietary (and released to the public when a new proprietary version is available). Thus the version of STRING needs to be cited. Moreover, the kinds of evidence that went into the inference of those edges should be indicated somewhere. They include co-expression, co-citation, and a variety of other functional associations. It simply cannot be inferred that all of these global associations are relevant for a particular cell type. A key to the data sources from which the displayed links derive is needed here.

If the authors wish to retain this diagram, it is certainly a reasonable place to start, but they need to avoid over-interpretation based on this diagram. For example, it's fine to indicate that they drew arbitrary dashed lines around a group of genes that they think might represent functional modules, but they should refrain from implying that the network provides more than a hypothesis (i.e. a starting point not a full interpretive framework). This is particularly relevant to the inference of cell-type specific functional links, since the STRING data tend to represent more of a global compendium. Thus, certain functional links may not be relevant in any individual cell type of interest, a point that is particularly salient here because the authors do find cell-type specific differences in phenotypic manifestations for individual genes.
Overall my sense is that the discussion on this topic, and the figure as well, would be better suited as part of the Discussion than the results, with a stronger emphasis on the exploratory and hypothesis-generating nature of these data.

9) P. 16: Second sentence of second paragraph needs to be rewritten.

10) P. 16: Sentence beginning "Regulation of division asynchrony during tissue growth ..." needs to be rewritten.

11) P. 17: "despite a coupled or separate regulation ..." Again, the authors cannot have it both ways. I suspect this is a language problem and that they do not mean "despite", but perhaps "whether".

12) P. 17: Last two sentences of first paragraph ("It should be noted ...") need to be rewritten. These two points do not go together, but the writing suggests that they are conceptually coupled. This whole section is rambling and should be streamlined.

13) P. 17: First sentence of second paragraph should be rewritten. The point is that "cell fate determinants appear to play an active role in cell-specific regulation of division timing, whereas cell cycle components appear to play a passive role." However I don't understand how the authors arrive at their "active" vs. "passive" conclusion here.

14) P. 18: First full sentence on this page ("Notably, inactivation ...") needs to be rewritten.

15) P. 18: I might suggest that the last section on the database be separated as its own paragraph. Instead of referring to the Materials and Methods, perhaps the authors could refer to their website instead? After all they are providing a beautiful dataset and a valuable resource for the community. For example, "To facilitate public access ... we have developed a web-accessible database available at http://mywebsite.org." Then discuss the kinds of data available in the database, followed by a short summary of the ways in which users can explore the data. This does not need to be one super long sentence; several short sentences that each describe a different essential aspect of the resource could serve to highlight the large amount of work the authors have done to make the dataset available in as extensive and useful way as they can. The dataset itself is very impressive, and it deserves to be highlighted as a resource (in my humble opinion).

Reviewer #2:

This revised manuscript has been edited and is substantially improved. While it was now easier to follow the main flow of the paper, the writing is still overly complicated in places (some examples below), and there are a number of additional issues that should be resolved or addressed, detailed below.

1)
cbp-1, cdk-8 are likely to be required for (full) zygotic transcription, and previous studies showed that transcription is required for cell cycle asymmetry between the E and MS lineages. This suggests that transcription could be required for ADS as well. Is this true? Is ADS lost after ama-1 RNAi? Does zygotic expression occur after tads-1 RNAi or other treatments that cause widespread ADS defects? This wouldn't be a surprising result but if true would be the most interesting result of the paper and should be addressed and emphasized more if true.

Is it possible that the "buffering effect" suggested or cbp-1 is due to variability of the RNAi and thus the level of leaky transcription in these experiments? A more interesting criteria for such "buffers" would be genes that don't cause E->MS changes but do increase variability (check Figure S2)

2)
Table S7 - It is still unclear to me why genes with overall slowdown can't be analyzed for ADS. Especially given comment 1.

3)
Abstract and throughout the text - "Cell fate determinants" - In most cases it seems this is used to mean "regulatory genes." Why not just use that term?

4)
p4 "Recent work on the cell cycle regulator, WEE-1.1, has demonstrated that the extended cell division timings in Ea and Ep are dependent on the high level expression of the protein induced by P2" - should be "extended cell cycle lengths"

5)
"reduction of division timings" - should be " reduction of cell cycle lengths?"

6)
"but the relative timings between cells were well maintained (Nair et al. 2013)" Also reference Schnabel 1997

7)
"division paces" (Various places) - should be "division pace"

8)
"develops into not more than a handful of cells" should be "contains only a handful of cells."

9)
p7 "cell division timings in cbp-1 RNAi embryos were not only greater in magnitude" - should be "cell cycle lengths in cbp-1 RNAi embryos were not only greater in magnitude"

10)
Fig. S4B - increase in SD of division timing with time. This is expected because of compounding errors from early divisions (for division times) and longer CC in later time points. Previous studies found that CC variation as a proportion of CC length was constant over time (Bao et al 2008, Richards et al 2013). Please clarify in the text.

11)
Fig S6 needs percentages on plot

12)
How were genes that "mediate cell fate specification directly or indirectly" defined? What about "maternal factors?"

13)
"C01A2.5 (renamed as tads-1 and approved by Wormbase)" - just call it tads-1 - don't need to justify here or state that wormbase has approved. Same for cbp-2.

14)
"Interestingly, our data demonstrated the role of Notch signaling in controlling ADS of ABplaaap (to develop into the part of left head) but not that of ABarpapp (to develop into the part of right head) though the two cells develop into the symmetric cell types (Fig 4A, Fig 5)." - this is not surprising given the known role of notch in inducing the fate of ABplaaap but not ABarpapp. Text should be rephrased to make this clear.

15)
p12 "they are found to function primarily on temporal regulation in the 16 cells" - 1) The authors can't say whether anything observed in this study is cell autonomous as many of the defects could be a result of earlier fate transformations or other non-autonomous effects 2) Few of the selected divisions are truly "fate symmetric." Rather they often produce different types of neurons, different cell types within the pharynx, etc. Even in cases where the fates were annotated the "same" by Sulston, the cell fates (as defined by patterns of morphology, migration, gene expression) may be different.

16)
"two Hox genes, ceh-13 and nob-1 are only involved in regulating of ADS during fate specification but not that during tissue growth" - the data don't show this, first due to the comment above, and due to the threshold used and possible redundancy, these factors could be important for ADS in cells not identified in this study.

17)
"Tissue growth" seems like an inappropriate term since there is no growth (just reductive cleavage divisions) occurring at these stages. Maybe "clonal development" would be better? Although see comment 15.

18)
"The result suggests that the factor may play a role in defining the hypodermis fate identity by timing the asymmetric division of its ancestor" - thisis speculation better suited to the discussion

19)
p13 "the involvement of transcription factors in regulating the ADS within body-wall muscle seems to be minimal," "control of overall ADS within the same cell fate seems more likely to share regulators than the control over ADS between fates." - Can the authors provide statistical validation of these claims and others in this paragraph? Such as fold enrichments and p value?

20)
"First they are the possible cell fate determinants that are expected to produce defects in fate

specification upon perturbation" - unclear in the text here and throughout whether these "cell fate determinants" were defined solely based on their biochemical function (TF, chromatin modifier etc) or prior evidence supporting a role in fate specification. Please clarify throughout.

21)
"indicating a coupled role of the pathway in temporal coordination and cell fate specification of the excretory cell" - would be better to say "indicating a role of the pathway in both temporal coordination and cell fate specification..."

22)
"The results demonstrated that control over asynchrony might serve at least partially to coordinate cell migrations for proper tissue growth."

arx-1 likely acts directly in the leading cells to facilitate ventral enclosure. The data don't show causality between the ADS and migration defects. Have the authors considered that the ADS defect could be secondary to a ventral enclosure defect, which could be as or more interesting than their interpretation, or that the RNAi might independently cause defects in both ADS and migration? Do other ventral enclosure mutants or RNAis also cause ADS defects in the excretory cell lineage?

23)
Fig. 6J please label units on the axes.

24)
p15 "It should be noted that" - remove these words. Variations of this phrase are used throughout the manuscript and in most cases the text would be easier to read without it.

25)
Fig. S10 - how was "potential crosstalk" (double headed arrows) defined? What is the meaning of the different colors (why is wrm-1 represented by a red ball, etc)

26)
"Part of these genes may simultaneously regulate..." Should read "Some of these genes..."

27)
p16 "tissue growth" - see concerns above for the use of this term.

28)
"development demands not only fate asymmetry, but also division asynchrony" - the results presented don't show that ADS is actually important for development, they only provide some insight into how they are regulated.

Reviewer #3:

General Remarks

In enjoyed reading the revised version of the manuscript. My concerns raised in the first round of revision were addressed adequately and the analysis and conclusions are coherent. I cannot comment on whether the concerns of the other reviewers have been addressed to their satisfaction but I personally recommend the revised manuscript for publication!

Please see below for minor suggestions. There is one change in the manuscript on page 8 that I do not find conclusive and would like the authors to comment on. (referring to the increased variation in cell division timing with developmental progression)

Minor Suggestions

Stylistic: I suggest a restructuring of the supplemental material: The 12 supplemental figures would be much easier to understand, if the figure legends would be placed below the figures.

Page 6, last line: refer to the phonics database.

Page 8, second paragraph: Is the reference to Table S1 in the first sentence of 'A framework for screening genes regulating ADS' necessary?

Page 10, last paragraph: The abbreviation for the gene tads-1 could be given since now the manuscript refers to ADS instead of TADS.

Figure 3, figure legend: (A) Shading for E lineage looks red, not brown. And 'MS' is in red, therefore the sentence: 'Note that 'E' (shaded in brown) became 'MS' (shaded in green)...' Does not make sense to me. (C, D) Images of space-filling models do not show cell migration, they show cell position.

Figure 6J: Why are there two dots per RNAi condition? Are the positions for two replicate embryos for each RNAi condition given? Do the two green dots represent tbx-33 and ceh-43 RNAi conditions?

Supplemental Table 2: What does the % stand for?

Supplemental Table 7: This table could be moved to the supplemental materials instead of providing a separate excel sheet.

Page 8, second paragraph:

I am aware that the last sentence of this paragraph was added to a reviewers comment, but I don't understand why. Maybe I misunderstood the way the standard deviations you give in Figs 4A and S4A,B. Aren't these calculated for the length of each cell division? How would these standard deviations be dependent on variation in earlier divisions? You give in Table S4 the variation for the length of each cell division and also the cumulative variation with respect to one early time registry point, so why do you speculate that the 'increased variation in division timing during later generations could be a product of cumulative variations...'?

I think the speculation you make here is very interesting, as I had noticed a similar increase in variability in earlier publications. I think it would add to your paper to clarify this point. If I understand correctly, you should have the data to analyze this properly! Your Figure S4B is a start but it did not convince the other reviewer.

I have just quickly plotted the cell names against the SD or accumulative SD columns. Of course the cumulative effect is greater, but it seems like there still is a trend to greater variation in timing between early and late cell divisions. (This could be due to a lengthening of cell division. To rule that out, the length of SD could even be taken relative to the length of that cell division.)

2nd Revision - authors' response 27 April 2015

(see next page)

Reviewer #1:

This resubmission by Ho et al. is greatly improved over the original version. Overall, this is a very nice piece of work. The authors have gone to great lengths to carefully address most of the comments from the first round of reviews, resulting in a greatly improved presentation. The narrative has been extensively revised and now reads much better, and the authors have made efforts to tighten and focus the interpretation and discussion of results. I have a few outstanding concerns, enumerated below, which I expect could be relatively easily addressed.

In particular, I would still like to see the Discussion tightened and streamlined in order to bring out the main conclusions from the study. There is a lot of data here that provides a nice foundation for downstream mechanistic studies. However, the authors do a lot of dancing around the fact that, without further analysis, the true functional dependencies between the factors they uncover in their screen cannot be clearly discerned. It would therefore be useful to simply state this, and then elaborate on what they think is a plausible scenario. But it needs to be clear that further analyses will be required to establish a mechanistic understanding of how asymmetric cell divisions and cell division timing asynchrony are coordinated.
We really appreciate the great efforts again in reviewing our manuscript and in providing the very constructive comments that help improve our manuscript. We agree that in our discussion, we have not conveyed our major points in a straightforward and intuitive way. We therefore made the following changes in the Discussion section.

A. We reorganized the first and second paragraph and pooled and rewrote the central points scattered within the two into the first paragraph of the revised version.
B. We moved the part on inference of the regulatory pathway on excretory cell into the Discussion section (2nd paragraph).
C. We included more sentences on the “Phenics” database and the way for access to its data.
D. This section was subjected to extensive copyediting and numerous grammar errors and typos were corrected.

We believe the revised paragraphs should be more tightened and streamlined for bringing out our main conclusions.

1) On page 7, a reference should be included to introduce the idea of a phenotypic "buffer" or "capacitor", since this concept has not been introduced prior to this point in the manuscript.

We changed the sentence “suggesting that the gene is possibly functioning as a phenotypic buffer or capacitor for temporal control of cell division” into “suggesting that the gene is possibly functioning as a phenotypic buffer or capacitor for achieving phenotypic robustness of temporal control of cell division” and included the citation (PMID:22821470).

2) Beginning on page 13 in the Results, and throughout the Discussion, there again appear a large number of grammatical errors and weird sentence constructs. These sections require further editorial attention before they could be considered suitable for publication.

Our colleagues have carefully proofread the relevant parts and corrected numerous errors.

3) On page 13, the authors persist in trying to use the word "asymmetric" to refer to both fate specification and division timing. As noted previously, "asymmetry" is a spatial concept, whereas "asynchrony" is a temporal one. This needs to be corrected.

“timing the asymmetric division of its ancestor” was changed into “regulating the division asynchrony of its ancestor” (page 13 first paragraph).

“The precursor of the excretory cell, ABplpapp, undergoes asymmetric division in terms of both cell fate and division timing” was changed into “Division of the precursor of the excretory cell, ABplpapp, shows both fate asymmetry and asynchrony” (page 13 last paragraph).

4) The discussion on excretory cell specification on pp. 13-14 is confusing and should be revised. The observations are that four genes influence both asynchrony and cell fate, whereas two affected asynchrony more than fate. Then, a discussion of Notch and Wnt signaling ensues (though the data for Notch are not presented here or cited from the literature). The authors alternately note that different results indicate either "coupled" regulation of the two processes or "discrete" regulation.

The difficulty I have with the way this section is written is the apparent implication that the results are contradictory as to whether these processes are

separable for a given cell type; instead I suspect that the intent is to comment upon the level of regulation at which these processes might be coupled, as revealed by RNAi of different gene products. This really does not come across clearly, however, and these comments regarding interpretation are probably best left to the Discussion.

This kind of expository problem is repeated several times in the following sections. The way the text comes across, the authors wish to have it both ways: the regulation appears to be both coupled and separable. Without further dissecting the pathways, effectors, and dependencies here, this comes across as very cloudy. The narrative would benefit from some clarification on these points.

The major point of this section is to demonstrate whether the genes found responsible for the ADS regulation in our screening are also responsible for fate specification using two types of cell fates as an example, i.e., excretory cell and hypodermis cell. CEH-26 and NHR-25 were used a fate marker for each respectively. The assumption here is that if a regulatory factor simultaneously controls ADS and cell fate specification (as judged by a fate marker), a coupled control of both processes is assumed. If a factor is found only to control ADS but not fate specification, control of two processes is assumed as separable.

In the case of excretory cell, four genes were found required for both fate specification and ADS control whereas the remaining two were shown to be dispensable for the fate specification (Fig 6 D&G) though they are required for ADS. Therefore, a coupled control by the four and a separable control by the remaining two are assumed. For the case of Notch signaling, its regulation on the excretory cell fate was derived from others (see cited literature), which is coincident with its regulation on ADS from our study. Therefore a coupled control is assumed. A new sentence was included to make this clear. In the case of hypodermis cell, Wnt signaling pathway and three TFs were used for the same purpose as that in the excretory cell. However, inactivation of the three TFs only caused a partial loss of the NHR-25 marker expression, which we also treated as a fate change. Therefore, a coupled regulation of both processes is assumed.

We did find the logical flow in this section was not as clear as what was intended to be. We therefore restructured some part of the section and included a conclusion sentence at the end of the section. Some grammar

errors were also corrected.

5) P. 15: "relative positions of]most perturbed embryos were significantly shifted compared to the wild-type embryos" does not make sense. Are the embryos themselves in a different place? Presumably the authors intend to refer to specific cells/cell divisions rather than embryos per se here.
The sentence "the relative positions of most perturbed embryos" was changed into "the relative positions of ABplpappa in most perturbed embryos". Sorry for the mistake!

6) P. 15: Why does a cell with an extra nucleus imply defective fate specification? Usually this signifies a failure of cytokinesis (with indirect downstream effects, such as failure in the asymmetric segregation of cell determinants pursuant).
This is inferred based on the following observation. Development of excretory cell from ABplpappa requires a programmed cell death of its posterior daughter. Cell death is also treated a cell fate. Survival of the posterior daughter cell could be responsible for the observed two nuclei. The observed two nuclei are widely separated from each other (Fig S9 O), which does not look they belong to a single cell.

7) P. 15: What are "thorn-like" canals?
"produced "thorn" like canals" was changed into "produced canals with "thorn" like protrudes" (Fig S9 K & MK), which means abnormal protrudes extending from canals versus the relative smooth canal in wild-type (Fig S9 J).

8) P. 15 and Figure S10: I really cannot see how a STRING representation of a bunch of genes that are involved in specification of a particular lineage results in "a putative gene network controlling [insert cell type here] specification" - particularly one that can be interpreted as being "initiated by maternal factors followed by Wnt and Notch pathways, and further relayed by transcription factors and chromatin modifiers". While a plausible scenario, this really seems like over-interpretation of the data.

In the supplementary figure, the composition of the STRING network needs to be more clearly described. The STRING database gets updated regularly, and the most current version is proprietary (and released to the public when a new proprietary version is available). Thus the version of STRING needs to be cited.

Moreover, the kinds of evidence that went into the inference of those edges should be indicated somewhere. They include co-expression, co-citation, and a variety of other functional associations. It simply cannot be inferred that all of these global associations are relevant for a particular cell type. A key to the data sources from which the displayed links derive is needed here.

If the authors wish to retain this diagram, it is certainly a reasonable place to start, but they need to avoid over-interpretation based on this diagram. For example, it's fine to indicate that they drew arbitrary dashed lines around a group of genes that they think might represent functional modules, but they should refrain from implying that the network provides more than a hypothesis (i.e. a starting point not a full interpretive framework). This is particularly relevant to the inference of cell-type specific functional links, since the STRING data tend to represent more of a global compendium. Thus, certain functional links may not be relevant in any individual cell type of interest, a point that is particularly salient here because the authors do find cell-type specific differences in phenotypic manifestations for individual genes.
Overall my sense is that the discussion on this topic, and the figure as well, would be better suited as part of the Discussion than the results, with a stronger emphasis on the exploratory and hypothesis-generating nature of these data.
Thanks very much for pointing out the caveat of the network! We agree that the network is exploratory at this stage and an over interpretation of the data is still obvious. As such, we moved the paragraph into Discussion and integrated it into the second last paragraph of the revised section. We rephrased the relevant sentences to emphasize the exploratory and hypothesis-generating nature of these data and include one sentence discussing about the caveat of the data from STRING for which the version number is included. We also removed the part on the functional relationship between the putative modules to minimize over interpretation of our data. However, we did cite all the data sources that establish the functional link between genes by STRING because we believe citing of STRING would suffice for the purpose. Inclusion of all the data sources would add another 10-20 references into our already crowded bibliography.

9) P. 16: Second sentence of second paragraph needs to be rewritten.
It was rewritten accordingly.

10) P. 16: Sentence beginning "Regulation of division asynchrony during tissue growth ..." needs to be rewritten.
It was rewritten into the sentence listed below and moved into the first paragraph of Discussion as mentioned in the beginning of our response.

“Regulation of division asynchrony during tissue growth may partially serve to determine the total rounds of cell division to control tissue size, whereas the similar regulation of division asynchrony during fate specification may mostly serve to coordinate cell migrations.”

11) P. 17: "despite a coupled or separate regulation ..." Again, the authors cannot have it both ways. I suspect this is a language problem and that they do not mean "despite", but perhaps "whether".
This sentence was deleted and the relevant parts were written as elaborated in the beginning of our responses.

12) P. 17: Last two sentences of first paragraph ("It should be noted ...") need to be rewritten. These two points do not go together, but the writing suggests that they are conceptually coupled. This whole section is rambling and should be streamlined.
These two sentences are conceptually coupled but are not detailed enough to convey the conceptual link. We modified the sentences and included more details to clarify our points here. The entire section was also re-organized and partially re-written to streamline the logical flow.

13) P. 17: First sentence of second paragraph should be rewritten. The point is that "cell fate determinants appear to play an active role in cell-specific regulation of division timing, whereas cell cycle components appear to play a passive role." However I don't understand how the authors arrive at their "active" vs. "passive" conclusion here.
The sentence was modified. The logic behind the sentence is that few of the cell cycle components are responsible for the ADS though their inactivation is commonly associated with global defects in division pace. In contrast, cell fate determinants tend to regulate division asynchrony, but they may exert their effect through differentially regulating the cell cycle components.

14) P. 18: First full sentence on this page ("Notably, inactivation ...") needs to be rewritten.

The sentence was rewritten and attached below.
"Inactivation of several other cell cycle components, for example *cdk-1*, *chk-1* (a DNA check-point kinase), *wee-1.3* (a kinase of Wee 1 family), and *air-2* (a gene encoding an Aurora-like protein), led to an early embryonic arrest (Supplementary Tables S1 and S7), which prevents a thorough interpretation of their roles in establishing cell fate asymmetry and division asynchrony."

15) P. 18: I might suggest that the last section on the database be separated as its own paragraph. Instead of referring to the Materials and Methods, perhaps the authors could refer to their website instead? After all they are providing a beautiful dataset and a valuable resource for the community. For example, "To facilitate public access ... we have developed a web-accessible database available at http://mywebsite.org." Then discuss the kinds of data available in the database, followed by a short summary of the ways in which users can explore the data. This does not need to be one super long sentence; several short sentences that each describe a different essential aspect of the resource could serve to highlight the large amount of work the authors have done to make the dataset available in as extensive and useful way as they can. The dataset itself is very impressive, and it deserves to be highlighted as a resource (in my humble opinion).
Thanks very much for the suggestion! We separated the paragraph on its own accordingly and included a few more sentences to describe the key contents of the database and it access method.

Reviewer #2:

This revised manuscript has been edited and is substantially improved. While it was now easier to follow the main flow of the paper, the writing is still overly complicated in places (some examples below), and there are a number of additional issues that should be resolved or addressed, detailed below.

1)
cbp-1, cdk-8 are likely to be required for (full) zygotic transcription, and previous studies showed that transcription is required for cell cycle asymmetry between the E and MS lineages. This suggests that transcription could be required for ADS as well. Is this true? Is ADS lost after ama-1 RNAi? Does zygotic expression occur after tads-1 RNAi or other treatments that cause widespread ADS defects? This wouldn't be a surprising result but if true would

be the most interesting result of the paper and should be addressed and emphasized more if true.

This is a very nice point. In theory, transcription should be directly or indirectly required for ADS eventually. We included a few sentences in the revised Discussion, which describe the possible role of differential modification of chromatin between the two daughters in establishing the asynchrony. This is based on the observation that a relatively higher occurrence of signaling pathways, TFs and chromatin modifiers was found in defining the ADS than other genes. Differential chromatin status can be treated as a proxy of expressivity of relevant genes in the affected regions. However, zygotic expression does occur in tads-1 RNAi embryo though the RNAi may produce a reduced level of expression. ADS was not calculated for ama-1 RNAi because most of the treated embryos arrested before 150 cells. It is likely that some of the ADS in the ama-1 RNAi embryo would be lost or reduced. Since our data did not provide direct evidence that transcription is required for ADS, to avoid over-interpretation of our data, we did not include this in our conclusion.

Is it possible that the "buffering effect" suggested or cbp-1 is due to variability of the RNAi and thus the level of leaky transcription in these experiments? A more interesting criteria for such "buffers" would be genes that don't cause E->MS changes but do increase variability (check Figure S2)
The "buffering effect" is suggested as one likely explanation, but we cannot rule out the possibility of leaky transcription that is responsible for the “buffering” effect and if true uncertain to what extent that would be.

2)
Table S7 - It is still unclear to me why genes with overall slowdown can't be analyzed for ADS. Especially given comment 1.
We are sorry about the potential caveat in our experimental design! The screen is designed to comb out genes that are responsible for cell-specific ADS that are reproducibly observed during the proliferation stage of *C. elegans* embryogenesis, and perturbation of these genes was expected to produce relatively “normal” development except the defects in ADS. Thus genes whose perturbation produces severe and overall slowdown of development were excluded for data analysis using an arbitrary cutoff. We admit that there are some genes that are required for the ADS but are missed in our screen due to the cutoff. However, we included all the ADS and absolute

cell cycle length for all the cells of each perturbation in our online database. One can easily pull out the genes of their interest and calculate the ADS or other timing events using the cutoff of their choice.

3)

Abstract and throughout the text - "Cell fate determinants" - In most cases it seems this is used to mean "regulatory genes." Why not just use that term?

As we dedicated an entire table (Table S8) to demonstrate that nearly all of the genes listed in the first five categories of Fig 5 encode a cell fate determinant because their inactivation is commonly associated with a fate change. We therefore decide to retain the term but include its definition at the end of first paragraph of Introduction, which reads “We define the cell fate determinant as any regulatory gene whose perturbation produces a defective fate specification.”.

4)

p4 "Recent work on the cell cycle regulator, WEE-1.1, has demonstrated that the extended cell division timings in Ea and Ep are dependent on the high level expression of the protein induced by P2" - should be "extended cell cycle lengths"

Changes are made accordingly.

5)

"reduction of division timings" - should be " reduction of cell cycle lengths?"

Changes are made throughout the manuscript as appropriate. Sentence of division pace is also re-written (page 3, 1st paragraph).

6)

"but the relative timings between cells were well maintained (Nair et al. 2013)" Also reference Schnabel 1997

Sorry for missing of the relevant citation. The reference is added.

7)

"division paces" (Various places) - should be "division pace"

Changes are made throughout the manuscript.

8)

"develops into not more than a handful of cells" should be "contains only a handful of cells."

Changes are made accordingly (page 5, 1st paragraph).

9)

p7 "cell division timings in cbp-1 RNAi embryos were not only greater in magnitude" - should be "cell cycle lengths in cbp-1 RNAi embryos were not only greater in magnitude"

Changes are made accordingly.

10)

Fig. S4B - increase in SD of division timing with time. This is expected because of compounding errors from early divisions (for division times) and longer CC in later time points. Previous studies found that CC variation as a proportion of CC length was constant over time (Bao et al 2008, Richards et al 2013). Please clarify in the text.

Bao et al showed that cell cycle length increases linearly over accumulative developmental time rather than the variation of cell cycle length changes constantly over time.

To address whether the variation of cell cycle length changes constantly over cell cycle length, we took the suggestion made by the reviewer 3 (see bottom of this file), i.e., plotting the ratio between the SD of cell cycle length and the corresponding cell cycle length over generations. We plotted the data from AB daughters as shown below. The data still show an increasing ratio in the later generations, i.e., from 5th to 7th generation. We believe this will also hold for the remaining lineages. It is surprising that the ratios are also high in the early generations. We don't understand the reasons at the moment. We therefore modified the relevant sentence into "Intriguingly, the standard deviations of cell cycle lengths increased with developing time during later generations" (page 8, 2nd paragraph).

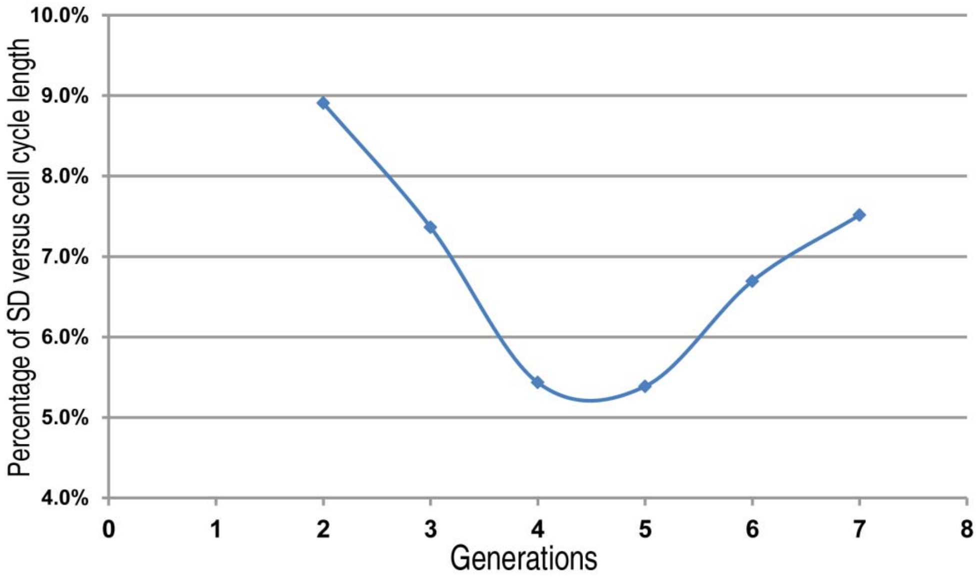

11)

Fig S6 needs percentages on plot

The percentages are added in the revised version.

12)

How were genes that "mediate cell fate specification directly or indirectly" defined? What about "maternal factors?"

They are defined as those whose perturbation produces defective fate specification as exemplified in Table S8. The maternal factors are also treated as a fate determinant. In practice, we refer to the genes from Early maternal to Chromatin modification as fate determinant, which was clarified in the main text.

13)

"C01A2.5 (renamed as tads-1 and approved by Wormbase)" - just call it tads-1 - don't need to justify here or state that wormbase has approved. Same for cbp-2.

Changes are made accordingly at page 10 (last paragraph) and page 11 (2nd paragraph) respectively.

14)

"Interestingly, our data demonstrated the role of Notch signaling in controlling

ADS of ABplaaap (to develop into the part of left head) but not that of ABarpapp (to develop into the part of right head) though the two cells develop into the symmetric cell types (Fig 4A, Fig 5)." - this is not surprising given the known role of notch in inducing the fate of ABplaaap but not ABarpapp. Text should be rephrased to make this clear.

The following sentences are added in page 11, 2nd paragraph.

"Consistent with this, Notch signaling has previously been found to be required for inducing the fate of ABplaaap but not ABarpapp (Hutter and Schnabel 1995; Moskowitz and Rothman 1996), suggesting a coupled regulation of division asynchrony and fate asymmetry in the cell by the signaling pathway."

15)

p12 "they are found to function primarily on temporal regulation in the 16 cells" - 1) The authors can't say whether anything observed in this study is cell autonomous as many of the defects could be a result of earlier fate transformations or other non-autonomous effects 2) Few of the selected divisions are truly "fate symmetric." Rather they often produce different types of neurons, different cell types within the pharynx, etc. Even in cases where the fates were annotated the "same" by Sulston, the cell fates (as defined by patterns of morphology, migration, gene expression) may be different.

We agree with the limitation of cell fate definition by the marker expression only which was adopted in this study.

16)

"two Hox genes, ceh-13 and nob-1 are only involved in regulating of ADS during fate specification but not that during tissue growth" - the data don't show this, first due to the comment above, and due to the threshold used and possible redundancy, these factors could be important for ADS in cells not identified in this study.

See the response above. It is true only with current cutoff and ignorance of the redundancy between different factors (page 13, 1st paragraph).

17)

"Tissue growth" seems like an inappropriate term since there is no growth (just reductive cleavage divisions) occurring at these stages. Maybe "clonal development" would be better? Although see comment 15.

We keep the term, but include a sentence which defines the term as "clonal development", which reads "We refer to tissue growth as clonal development

based on the expression of a tissue-specific marker”.

18)

"The result suggests that the factor may play a role in defining the hypodermis fate identity by timing the asymmetric division of its ancestor" - thisis speculation better suited to the discussion

Given the lengthy contents in Discussion section, we keep the sentence as it is.

19)

p13 "the involvement of transcription factors in regulating the ADS within body-wall muscle seems to be minimal," "control of overall ADS within the same cell fate seems more likely to share regulators than the control over ADS between fates." - Can the authors provide statistical validation of these claims and others in this paragraph? Such as fold enrichments and p value?

There is an apparent enrichment of regulatory factors for one fate versus others. For example, transcription factors are enriched for neuron as opposed to the remaining fates. We have thought about the statistics on this this claim, but it seems the available data in Fig 5 seems not enough to warrant a robust statistical analysis. Therefore, such an analysis has not been made.

20)

"First they are the possible cell fate determinants that are expected to produce defects in fate specification upon perturbation" - unclear in the text here and throughout whether these "cell fate determinants" were defined solely based on their biochemical function (TF, chromatin modifier etc) or prior evidence supporting a role in fate specification. Please clarify throughout.

The broad definition of cell fate determinant is added at the end of 1st paragraph of page 3. A more specific definition of the terms is given at line 13-15 in the 2nd paragraph of page 10.

21)

"indicating a coupled role of the pathway in temporal coordination and cell fate specification of the excretory cell" - would be better to say "indicating a role of the pathway in both temporal coordination and cell fate specification..."

Changes are made accordingly (1st paragraph of page 14)

22)

"The results demonstrated that control over asynchrony might serve at least partially to coordinate cell migrations for proper tissue growth."

arx-1 likely acts directly in the leading cells to facilitate ventral enclosure. The data don't show causality between the ADS and migration defects. Have the authors considered that the ADS defect could be secondary to a ventral enclosure defect, which could be as or more interesting than their interpretation, or that the RNAi might independently cause defects in both ADS and migration? Do other ventral enclosure mutants or RNAis also cause ADS defects in the excretory cell lineage?
Yes, we found arx-1 is only required for controlling ADS and cell migration but not for excretory cell fate specification. However the causality between the ADS and migration defects could not be established with our data. We speculate that in many cases the timing of cell division may serve as a cause for coordinated cell migration. Nevertheless, we cannot rule out the possibility of an opposite relationship. Extra data will be required to establish the causality relationship between the two cellular events. We are not aware of the effect of other ventral enclosure mutants or RNAi on the ADS defects in the excretory cell lineage.

23)
Fig. 6J please label units on the axes.
The embryos were normalized to 1.0 for each dimension. That is to say the position for each axis is relative and no unit applies.

24)
p15 "It should be noted that" - remove these words. Variations of this phrase are used throughout the manuscript and in most cases the text would be easier to read without it.
They are removed as appropriate throughout the manuscript.

25)
Fig. S10 - how was "potential crosstalk" (double headed arrows) defined? What is the meaning of the different colors (why is wrm-1 represented by a red ball, etc)
A "potential crosstalk" was arbitrarily defined based on the observation that ADS for some cells are subjected to regulation by both Wnt and Notch signaling pathways, which suggests a potential crosstalk of regulation in these

cells. The ball color for each gene is automatically determined by STRING, presumably due to its “hub” status.

26)
"Part of these genes may simultaneously regulate..." Should read "Some of these genes..."
“Part of these genes” into “Some of these genes identified in our screen”
Change is made accordingly (page 14, 2nd paragraph)

27)
p16 "tissue growth" - see concerns above for the use of this term.
See response above.

28)
"development demands not only fate asymmetry, but also division asynchrony" - the results presented don't show that ADS is actually important for development, they only provide some insight into how they are regulated.
Agree.

Reviewer #3:

March 2015

General Remarks

In enjoyed reading the revised version of the manuscript. My concerns raised in the first round of revision were addressed adequately and the analysis and conclusions are coherent. I cannot comment on whether the concerns of the other reviewers have been addressed to their satisfaction but I personally recommend the revised manuscript for publication!

Please see below for minor suggestions. There is one change in the manuscript on page 8 that I do not find conclusive and would like the authors to comment on. (referring to the increased variation in cell division timing with developmental progression)
The reviewer suggests that the variation observed in a later generation may be genetically associated with the variation in the earlier generation. Please see our response at item 10 at page 11.

Minor Suggestions

Stylistic: I suggest a restructuring of the supplemental material: The 12 supplemental figures would be much easier to understand, if the figure legends would be placed below the figures.
We agree with the point. However, the journal editor will determine the final arrangement of the figures and its legends.

Page 6, last line: refer to the phonics database.
Reference is made accordingly.

Page 8, second paragraph: Is the reference to Table S1 in the first sentence of 'A framework for screening genes regulating ADS' necessary?
Sorry for the mistake! Only Table S3 is referred to now.

Page 10, last paragraph: The abbreviation for the gene tads-1 could be given since now the manuscript refers to ADS instead of TADS.
This might cause confusion because "ADS" used now has different meaning from that in "TADS".

Figure 3, figure legend: (A) Shading for E lineage looks red, not brown. And 'MS' is in red, therefore the sentence: 'Note that 'E' (shaded in brown) became 'MS' (shaded in green)...' Does not make sense to me. (C, D) Images of space-filling models do not show cell migration, they show cell position.
Brown is changed into red. The relevant parts were re-written to minimize the confusion.

Figure 6J: Why are there two dots per RNAi condition? Are the positions for two replicate embryos for each RNAi condition given? Do the two green dots represent tbx-33 and ceh-43 RNAi conditions?
Yes, the positions for two replicate embryos for each RNAi were given for each gene. The two green dots represent tbx-33 and ceh-43 RNAi respectively in which only a single replicate shows significant deviation from wild-type.

Supplemental Table 2: What does the % stand for?
It represents the percentage of the genes in current GO category out of the total number of input genes, which is indicated in the Table legend.

Supplemental Table 7: This table could be moved to the supplemental materials instead of providing a separate excel sheet.
Change is made accordingly.

Page 8, second paragraph:

I am aware that the last sentence of this paragraph was added to a reviewers comment, but I don't understand why. Maybe I misunderstood the way the standard deviations you give in Figs 4A and S4A,B. Aren't these calculated for the length of each cell division? How would these standard deviations be dependent on variation in earlier divisions? You give in Table S4 the variation for the length of each cell division and also the cumulative variation with respect to one early time registry point, so why do you speculate that the 'increased variation in division timing during later generations could be a product of cumulative variations...'?

I think the speculation you make here is very interesting, as I had noticed a similar increase in variability in earlier publications. I think it would add to your paper to clarify this point. If I understand correctly, you should have the data to analyze this properly! Your Figure S4B is a start but it did not convince the other reviewer.

I have just quickly plotted the cell names against the SD or accumulative SD columns. Of course the cumulative effect is greater, but it seems like there still is a trend to greater variation in timing between early and late cell divisions. (This could be due to a lengthening of cell division. To rule that out, the length of SD could even be taken relative to the length of that cell division.)

Please see our responses at page 11 (item 10).

3rd Editorial Decision 13 May 2015

Thank you again for submitting your work to Molecular Systems Biology. We are now satisfied with the modifications made and we think that the study is suitable for publication, pending some minor modifications listed below.

- First of all, I would like to ask you to include one or two sentences in the main manuscript text, providing clear answers to comments 15 and 16 of reviewer #2 (there are answers in the point by point response but they are not included in the manuscript).

- Moreover, I have edited the manuscript text in order to correct a few remaining language issues and to provide a precise answer to comment 19 of reviewer #2. I would be grateful if you could go through the text and let me know if you agree with these text changes or if you would like to suggest further modifications.

Thank you for submitting this paper to Molecular Systems Biology.

3rd Revision - authors' response 18 May 2015

Here we revised our manuscript based on your comments. The details of the revised parts are attached in the bottom, with our responses highlighted in blue. Please do not hesitate to let us know if you have any other questions and appreciate your efforts again in handling our manuscript.

- First of all, I would like to ask you to include one or two sentences in the main manuscript text, providing clear answers to comments 15 and 16 of reviewer #2 (there are answers in the point by point response but they are not included in the manuscript).

The following two sentences were added at the end of first paragraph in page 13.

"One should be aware of the limitation of the thresholds or the tissue markers used in the screening. The genes identified for the differential control of ADS may vary when different thresholds or tissue markers are used."

- Moreover, I have edited the manuscript text in order to correct a few remaining language issues and to provide a precise answer to comment 19 of reviewer #2. I would be grateful if you could go through the text and let me know if you agree with these text changes or if you would like to suggest further modifications.
We have gone through the text and the detailed changes or solutions are listed below.

In the sentence "which is defined as the duration of a given cell division throughout the development", the word "division" was deleted.
The sentence "WEE-1.1, has demonstrated that the extended cell cycle lengths in Ea and Ep are dependent on the high expression levels of the protein induced by P2." was changed into the following one.
"WEE-1.1, has demonstrated that the extended cell cycle lengths in Ea and Ep are dependent on P2-EMS signal."
"Nevertheless, conclusive statistical support for this finding cannot be provided by the data available, and therefore the differential involvement of transcription factors in the different processes remains to be further examined."
The above sentences you added were adopted as it is.
